# Supplementary material for: Membrane-bound mRNA immunogens lower the threshold to activate HIV Env V2 apex-directed broadly neutralizing B cell precursors in humanized mice
Source: Immunity. 2022 Nov 8;55(11):2168–2186.e6. doi: 10.1016/j.immuni.2022.09.003 (PMC9671093; doi:10.1016/j.immuni.2022.09.003)
Supplement: Document S1. Figures S1–S7 [file mmc1.pdf]

Immunity, Volume *55*

Supplemental information

Membrane-bound mRNA immunogens lower the threshold to activate HIV Env V2 apex-directed broadly neutralizing B cell precursors in humanized mice

Eleonora Melzi, Jordan R. Willis, Krystal M. Ma, Ying-Cing Lin, Sven Kratochvil, Zachary T. Berndsen, Elise A. Landais, Oleksandr Kalyuzhniy, Usha Nair, John Warner, Jon M. Steichen, Anton Kalyuzhniy, Amber Le, Simone Pecetta, Manfredo Perez, Kathrin Kirsch, Stephanie R. Weldon, Samantha Falcone, Sunny Himansu, Andrea Carfi, Devin Sok, Andrew B. Ward, William R. Schief, and Facundo D. Batista

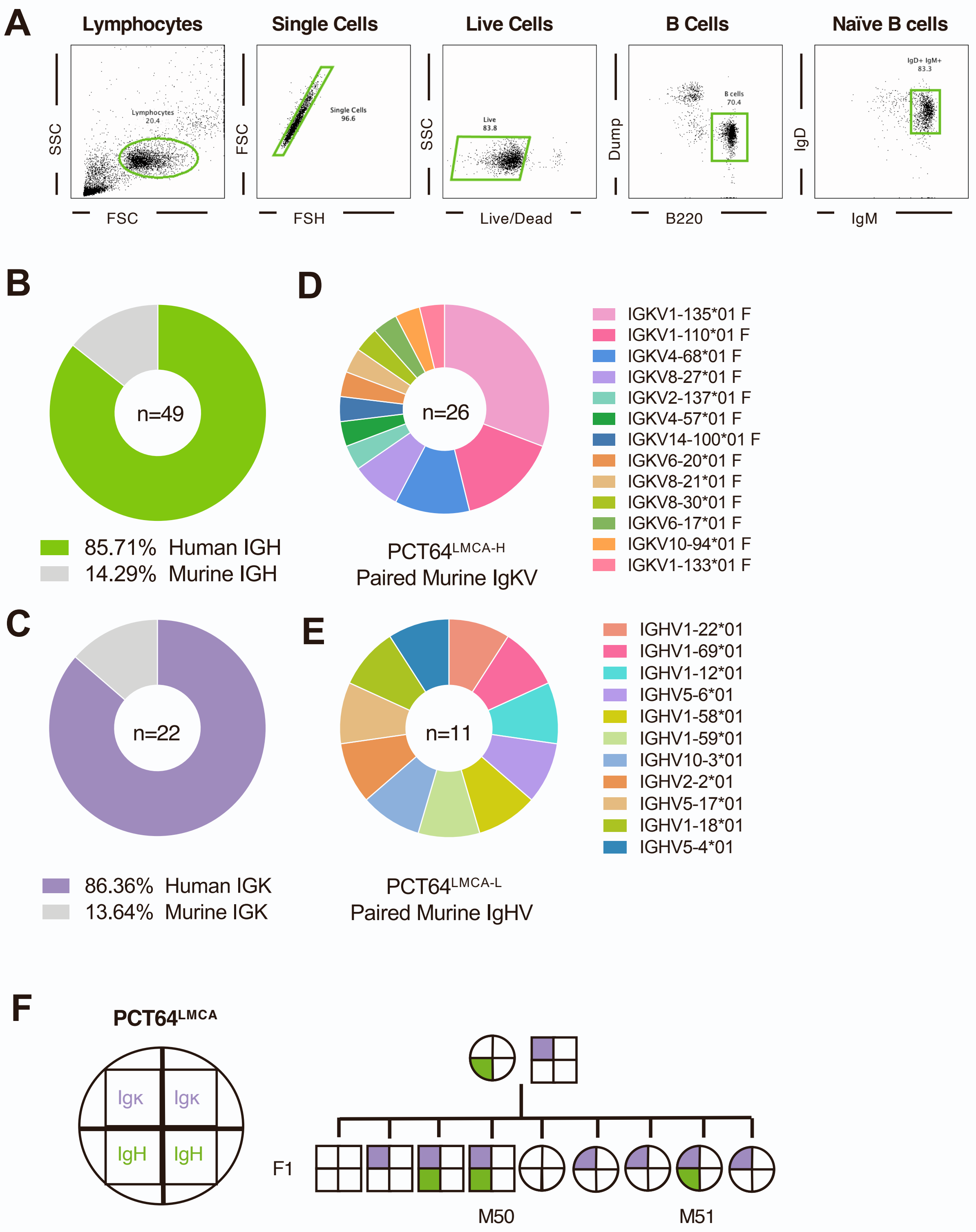

G

Line	# litters	# pups	# knock-in (KI) pups			KI pup frequency (%)		
			H	*κ*	*H, κ*	*H*	*κ*	*H, κ*
**LMCA**	6	49	22	22	11	44.8	44.8	22.4

Figure S1. Generation of a human Ig least mutated common ancestor (LMCA) PCT64 knock-in mouse, related to Figure 1.

A. Flow cytometry gating strategy for sorting and sequencing single naïve B cell from knock-in mice generated with CRISPR-Cas9.

B. Pie chart showing frequency of amplified human PCT64 LMCA heavy chain (green) and murine heavy chain (grey), from single cell BCR sequencing of naïve B cells in PCT64^{LMCA} IGH mice (PCT64$^{LMCA\text{-}H}$) (n=49).

C. Pie chart shows frequency of amplified human PCT64 LMCA light chain (purple) and murine heavy chain (grey), from single cell BCR sequencing of naïve B cells in PCT64 LMCA IGK mice (PCT64$^{LMCA\text{-}L}$) (n=22).

D. PCT64$^{LMCA\text{-}H}$ paired murine light chains V genes (n=26).

E. PCT64$^{LMCA\text{-}L}$ paired murine heavy chains V genes (n=11).

F. Breeding schematic for PCT64$^{LMCA\text{-}H}$ and PCT64$^{LMCA\text{-}L}$ mouse line. Squares represent males and circles represent females. Upper halves indicate IGK and lower halves IGH, as per key at left.

G. Transmission frequency of IGH (H) and IGK (K) to the progeny.

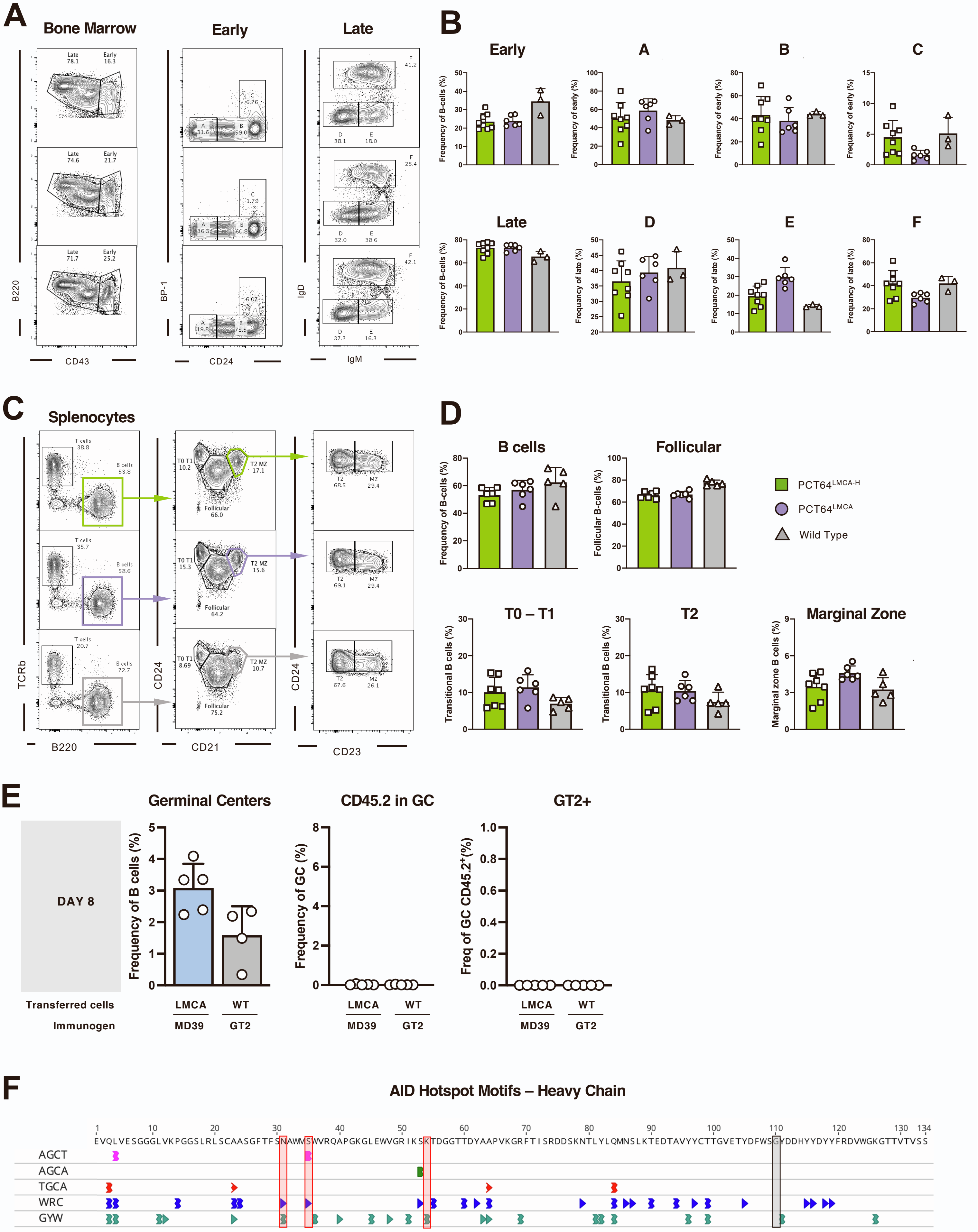
A
Bone Marrow
Early
Late
B220
CD43
BP-1
CD24
IgD
IgM
B
Early
A
B
C
Late
D
E
F
Frequency of B-cells (%)
Frequancy of early (%)
Frequancy of late (%)
C
Splenocytes
TCRb
B220
CD24
CD21
CD23
T cells
B cells
T0 T1
T2 MZ
Follicular
D
B cells
Follicular
Frequency of B-cells (%)
Follicular B-cells (%)
PCT64LMCA-H
PCT64LMCA
Wild Type
T0 – T1
T2
Marginal Zone
Transitional B cells (%)
Marginal zone B cells (%)
E
DAY 8
Germinal Centers
CD45.2 in GC
GT2+
Frequency of B cells (%)
Frequency of GC (%)
Freq of GC CD45.2+(%)
Transferred cells
Immunogen
LMCA
WT
MD39
GT2
F
AID Hotspot Motifs – Heavy Chain
EVQLVESGGGLVKPGGSLRLSCAASGFTFSNAWMSWVRQAPGKGLEWVGRIKSKTDGGTTDYAAPVKGRFTISRDDSKNTLYLQMNSLKTEDTAVYYCTTGVETYDFWSGYDDHYYDYYFRDVWGKGTTVTVSS
AGCT
AGCA
TGCA
WRC
GYW

Figure S2. B lymphocyte development in PCT64^{LMCA-H} and PCT64^{LMCA} mice, related to Figures 1 and 2.

A. Representative FACS plots of bone marrow progenitor cells isolated from PCT64^{LMCA-H}, PCT64^{LMCA} and WT mice and gating strategy applied for the quantification of early (A, B and C) and late (D, E and F) subfraction of B cell developmental stages accordingly to (Hardy et al. 1991)

B. Quantification of early (A, B and C) and late (D, E and F) subfraction of B cell developmental stages in bone marrow from PCT64^{LMCA-H} (green), PCT64^{LMCA} (purple) and wild type (WT) (grey) mice (as in A). LMCA-H n=8, LMCA n=6, WT controls n=3. Data are presented as mean ± SD.

C. Representative FACS plots of splenocytes isolated from PCT64^{LMCA-H}, PCT64^{LMCA} and WT mice and gating strategy applied for the quantification of splenic B cells and their differentiation into Follicular, Transitional (T0, T1, T2) and Marginal Zone B cells.

D. Quantification of splenic B cells and their differentiation into Follicular, Transitional (T0, T1, T2) and Marginal Zone B cells in spleens from PCT64^{LMCA-H} (green), PCT64^{LMCA} (purple) and Wild Type (grey) mice (as in C). LMCA and LMCA-H n=6, WT controls n=5. Data are presented as mean ± SD.

E. Quantification of B cell responses in spleen at 8 days after immunization. CD45.1 WT mice received either 500,000 PCT64^{LMCA} B cells and were immunized with MD39-SOSIP trimers or received 500,000 wildtype CD45.2 B cells and were immunized with GT2 trimers. Experiments were performed in duplicate, one is shown (n=5). Data are presented as mean ± SD.

F. Analysis of the activation-induced deaminase (AID) enzyme binding sites in PCT64 LMCA heavy chain. AA position of enriched on-track mutations are highlighted (31, 35, 52b, 100d).

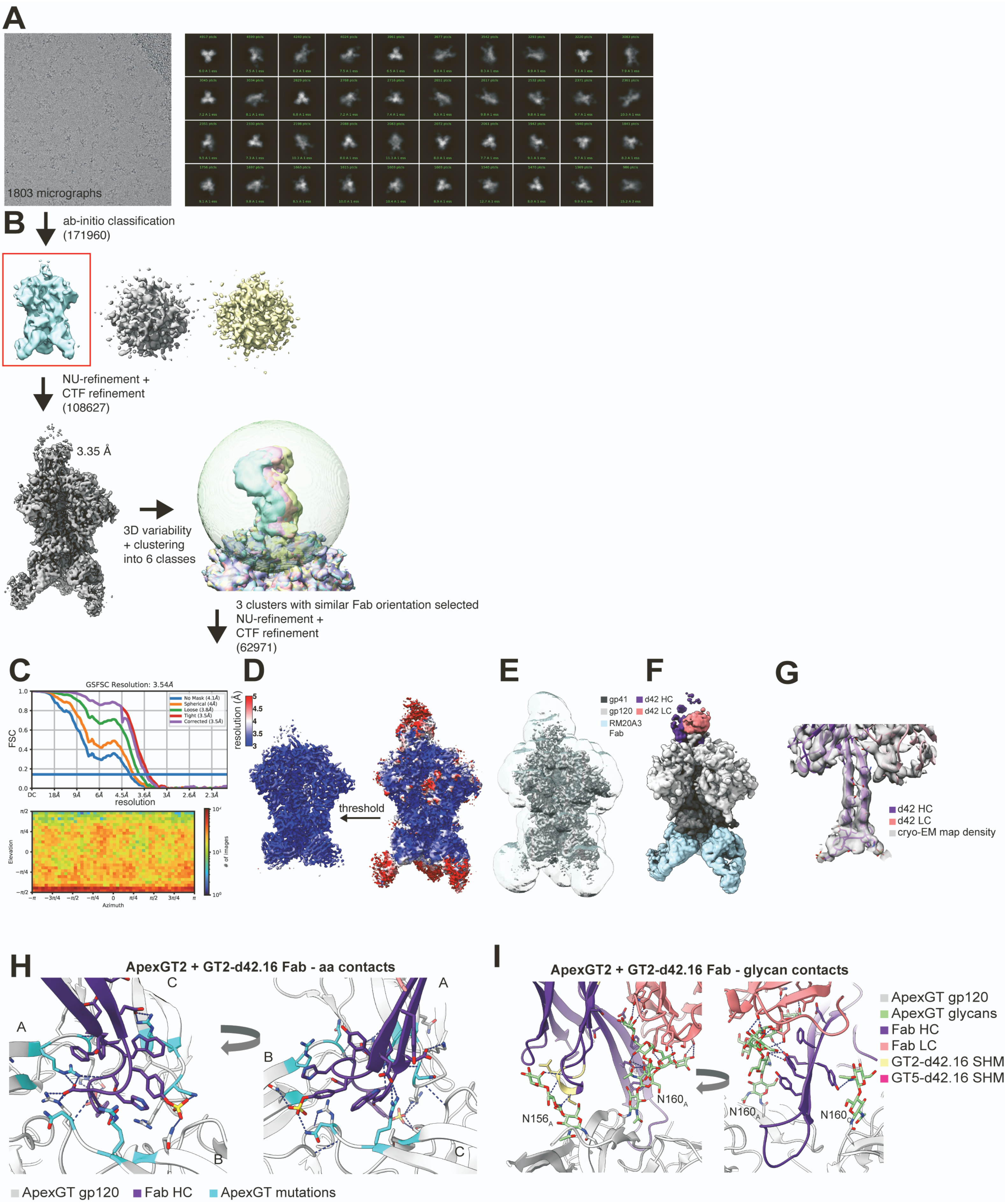
A
1803 micrographs
B
ab-initio classification
(171960)
NU-refinement +
CTF refinement
(108627)
3.35 Å
3D variability
+ clustering
into 6 classes
3 clusters with similar Fab orientation selected
NU-refinement +
CTF refinement
(62971)
C
GSFSC Resolution: 3.54Å
No Mask (4.1Å)
Spherical (4Å)
Loose (3.8Å)
Tight (3.5Å)
Corrected (3.5Å)
FSC
resolution
Elevation
Azimuth
# of images
D
resolution (Å)
threshold
E
gp41
gp120
RM20A3
Fab
d42 HC
d42 LC
F
G
d42 HC
d42 LC
cryo-EM map density
H
ApexGT2 + GT2-d42.16 Fab - aa contacts
ApexGT gp120
Fab HC
ApexGT mutations
I
ApexGT2 + GT2-d42.16 Fab - glycan contacts
N156A
N160A
N160A
N160
ApexGT gp120
ApexGT glycans
Fab HC
Fab LC
GT2-d42.16 SHM
GT5-d42.16 SHM

Figure S3. Cryo-EM data processing workflow for ApexGT2 in complex with GT2-d42.16 Fab, related to Figure 2.

A. Raw EM micrograph and 2-D class averages of selected particles.

B. 3-D data processing workflow.

C. Fourier shell correlation and angular distribution plots.

D. Local resolution estimates.

E. Mask used for refinement and sharpening.

F. Map segmentation.

G. Isolated cryo-EM map density within a 3Å radius around Fab residues.

H. Close up of the epitope/paratope region of ApexGT2 + G2-d42.16 showing hydrogen-bonding interactions with gp120 amino acid residues.

I. Close up of the epitope/paratope region of ApexGT2 + GT2-d42.16 showing hydrogen-bonding interactions with ApexGT2 glycans.

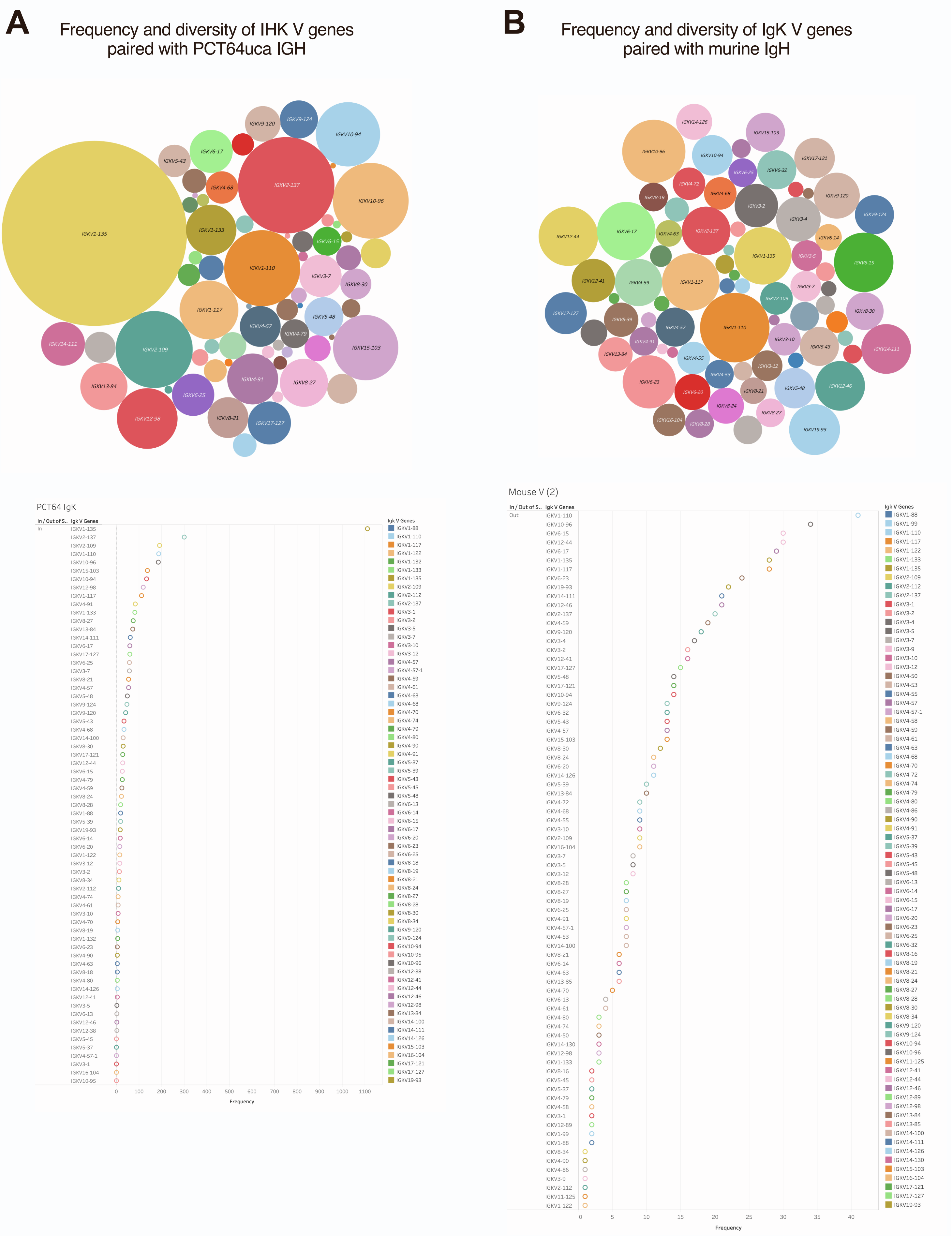
A
Frequency and diversity of IHK V genes paired with PCT64uca IGH
B
Frequency and diversity of IgK V genes paired with murine IgH
PCT64 IgK
In / Out of S..
Igk V Genes
Frequency
Mouse V (2)
In / Out of S..
Igk V Genes
Frequency

Figure S4. Light chain repertoire characterization in PCT64^{LMCA-H} mice, related to Figure 3.

A. Bubble graph representing frequency and diversity of murine IGK V genes paired with PCT64 IGH in a naïve PCT64^{LMCA-H} mouse (n=3888). Bubble size represent relative frequency, colors indicate different V gene. Below, detailed legend of all the isolated IGK V genes and relative frequency.

B. Bubble graph representing frequency and diversity of murine IGK V genes paired with murine IGH in a naïve PCT64^{LMCA-H} mouse (n=815). Bubble size represent relative frequency, colors indicate different V gene. Below, detailed legend of all the isolated IGK V genes and relative frequency.

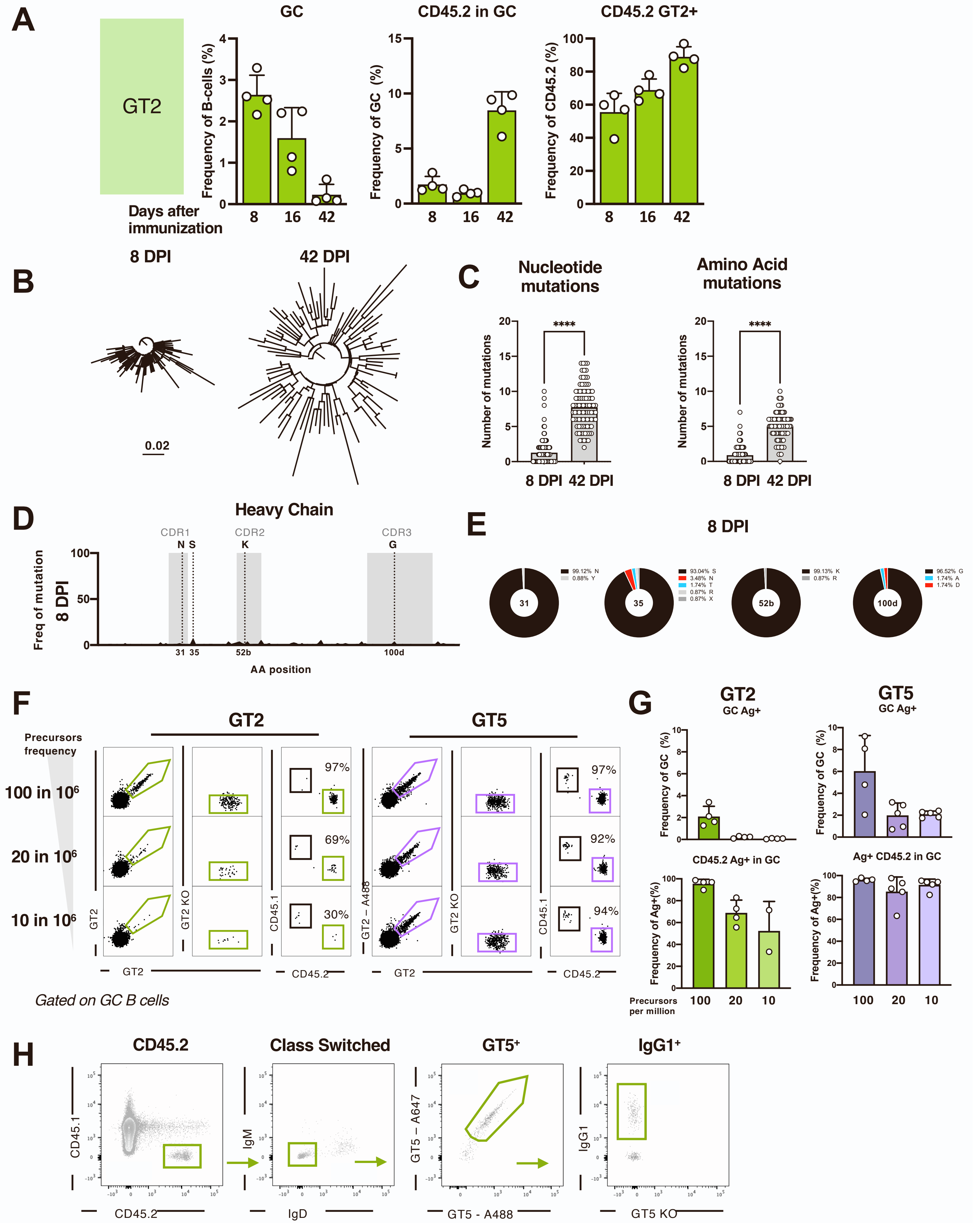

A
GT2
GC
CD45.2 in GC
CD45.2 GT2+
Frequency of B-cells (%)
Frequency of GC (%)
Frequency of CD45.2 (%)
Days after immunization
8
16
42
B
8 DPI
42 DPI
0.02
C
Nucleotide mutations
Amino Acid mutations
Number of mutations
****
8 DPI
42 DPI
D
Heavy Chain
CDR1
CDR2
CDR3
N S
K
G
Freq of mutation
8 DPI
31 35
52b
100d
AA position
E
8 DPI
99.12% N
0.88% Y
93.04% S
3.48% N
1.74% T
0.87% R
0.87% X
99.13% K
0.87% R
96.52% G
1.74% A
1.74% D
31
35
52b
100d
F
Precursors frequency
100 in 10⁶
20 in 10⁶
10 in 10⁶
GT2
GT5
GT2
GT2 KO
CD45.1
GT2 – A488
CD45.2
97%
69%
30%
97%
92%
94%
Gated on GC B cells
G
GT2
GC Ag+
GT5
GC Ag+
Frequency of GC (%)
CD45.2 Ag+ in GC
Ag+ CD45.2 in GC
Frequency of Ag+(%)
Precursors per million
100
20
10
H
CD45.2
Class Switched
GT5⁺
IgG1⁺
CD45.1
IgM
GT5 – A647
IgG1
CD45.2
IgD
GT5 - A488
GT5 KO

Figure S5. GT2 immunization activates PCT64 heavy chain with different light chains and GC competition after GT2 and GT5 immunization, related to Figures 3, 4 and 5.

A. FACS quantification of germinal center (GC) responses, CD45.2 cells inside GC, and GT2 binding B cells at 8, 16 and 42 days after immunization with GT2. Recipient mice received 500,000 $CD45.2^{+}$ $PCT64^{LMCA\text{-}H}$ B cells and responses were analyzed in the spleen. Experiments were performed in triplicate, one is shown (n=4/group). Data are presented as mean ± SD.

B. Phylogenetic clonal lineage trees showing diversification of the $PCT64^{LMCA}$ IGH from day 8 to day 42 after immunization. Branch length is representative of sequence distance.

C. Number of total nucleotide and amino acid mutations acquired in $PCT64^{LMCA\text{-}H}$ heavy chain V genes (IGHV) at 8 (n=114) and 42 (n=106) DPI. P values calculated by Mann-Whitney test, ****P < 0.0001.

D. Frequencies of heavy chain AA mutations observed per residue at day 8 post-immunization in $PCT64^{LMCA}$ heavy chain. HCDRs are highlighted in gray.

E. Distribution of selected PCT64 LMCA B cell heavy chain aa mutations in positions 31, 35, 52b and 100d at 8 dpi. Mutations present in mature PCT64 are in red, mutations present in PCT64-lineage early isolates are in blue, original LMCA amino acids are in black, other mutations are in grey.

F. Representative FACS plots of antigen-specific germinal center responses after immunization with GT2 (green) or GT5 (purple) (10 ug, adjuvanted with Sigma) in mice with 100, 20 and 10 per 10^6 $PCT64^{LMCA}$ precursor B cells.

G. Quantification of responses in F. Experiments were performed in triplicate, one is shown (n=4, for GT5 10,20:10^6 n=5). Data are presented as mean ± SD.

H. FACS gating strategy for sorting of class switched antigen specific cells at 42 dpi.

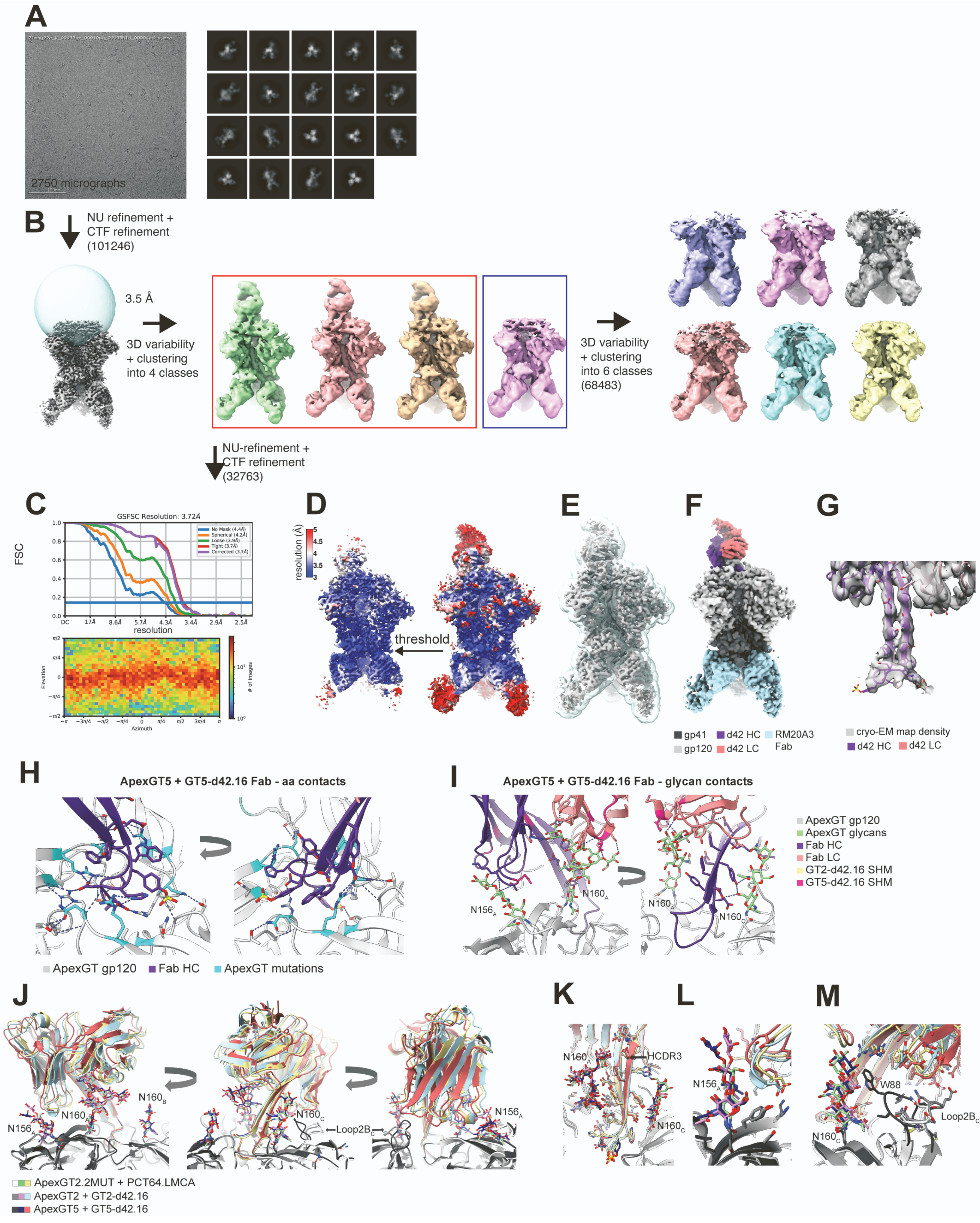
A
2750 micrographs
B
NU refinement + CTF refinement (101246)
3.5 Å
3D variability + clustering into 4 classes
3D variability + clustering into 6 classes (68483)
NU-refinement + CTF refinement (32763)
C
GSFSC Resolution: 3.72Å
FSC
resolution
Elevation
Azimuth
# of images
D
resolution (Å)
threshold
E
F
G
gp41
gp120
d42 HC
d42 LC
RM20A3 Fab
cryo-EM map density
d42 HC
d42 LC
H
ApexGT5 + GT5-d42.16 Fab - aa contacts
ApexGT gp120
Fab HC
ApexGT mutations
I
ApexGT5 + GT5-d42.16 Fab - glycan contacts
ApexGT gp120
ApexGT glycans
Fab HC
Fab LC
GT2-d42.16 SHM
GT5-d42.16 SHM
J
K
L
M
HCDR3
W88
Loop2B
ApexGT2.2MUT + PCT64.LMCA
ApexGT2 + GT2-d42.16
ApexGT5 + GT5-d42.16

Figure S6. Cryo-EM data processing workflow for ApexGT5 in complex with GT5-d42.16 Fab, related to Figure 5.

A. Raw EM micrograph and 2-D class averages of selected particles.

B. 3-D data processing workflow.

C. Global Fourier shell correlation and angular distribution plots.

D. Local resolution estimates.

E. Mask used for refinement and sharpening.

F. Map segmentation.

G. Isolated cryo-EM map density within a 3Å radius around Fab residues.

H. Close up of the epitope/paratope region of ApexGT5 + GT5-d42.16 showing hydrogen bonding interactions with gp120 amino acid residues.

I. Close up of the epitope/paratope region of ApexGT5 + GT5-d42.16 showing hydrogen bonding interactions with ApexGT2 glycans

J. Structures of ApexGT2.2MUT + PCT64.LMCA, ApexGT2 + GT2-d42.16, and ApexGT5 + GT5-d42.16 aligned and overlayed viewed from 3 different angles.

K. Close up of the HCDR3 domains and N160gp120A and B glycans,

L. N156gp120A glycan,

M. And loop2B of protomer C along with N160gp120A glycan.

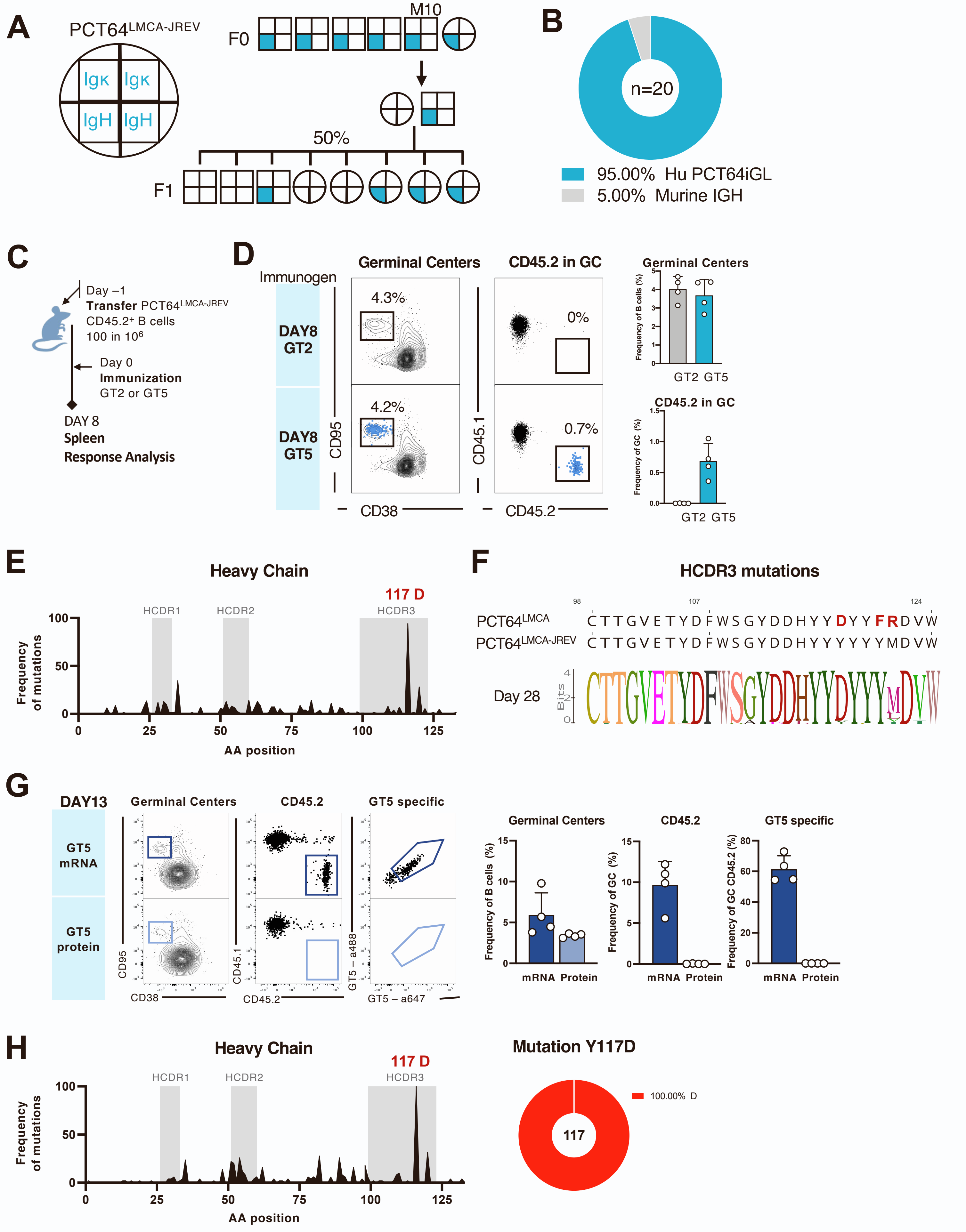
A
PCT64LMCA-JREV
Igκ
Igκ
IgH
IgH
F0
M10
50%
F1
B
n=20
95.00% Hu PCT64iGL
5.00% Murine IGH
C
Day –1
Transfer PCT64LMCA-JREV
CD45.2+ B cells
100 in 10^6
Day 0
Immunization
GT2 or GT5
DAY 8
Spleen
Response Analysis
D
Immunogen
Germinal Centers
CD45.2 in GC
DAY8 GT2
DAY8 GT5
4.3%
4.2%
0%
0.7%
CD95
CD38
CD45.1
CD45.2
Germinal Centers
Frequency of B cells (%)
GT2 GT5
CD45.2 in GC
Frequency of GC (%)
GT2 GT5
E
Heavy Chain
117 D
HCDR1
HCDR2
HCDR3
Frequency of mutations
AA position
F
HCDR3 mutations
PCT64LMCA C T T G V E T Y D F W S G Y D D H Y Y D Y Y F R D V W
PCT64LMCA-JREV C T T G V E T Y D F W S G Y D D H Y Y Y Y Y Y M D V W
98
107
124
Day 28
Bits
G
DAY13
Germinal Centers
CD45.2
GT5 specific
GT5 mRNA
GT5 protein
CD95
CD38
CD45.1
CD45.2
GT5 – a488
GT5 – a647
Germinal Centers
Frequency of B cells (%)
mRNA Protein
CD45.2
Frequency of GC (%)
mRNA Protein
GT5 specific
Frequency of GC CD45.2 (%)
mRNA Protein
H
Heavy Chain
117 D
HCDR1
HCDR2
HCDR3
Frequency of mutations
AA position
Mutation Y117D
117
100.00% D

Figure S7. Generation of a PCT64 LMCA.JREV mouse and immunization strategies, related to Figure 7.

A. Breeding and transmission of the knock-in IGH PCT64$^{LMCA.JREV}$ to the progeny.

B. Pie chart showing frequency of amplified human PCT64$^{LMCA.JREV}$ heavy chain (teal) and murine heavy chain (grey), from single cell BCR sequencing of naïve B cells in a PCT64$^{LMCA.JREV}$ IGH (PCT64$^{LMCA.JREV-H}$) (n=20) mouse (M10).

C. Schematic of immunization study design. Recipient mice received 100 PCT64$^{LMCA.JREV}$ per10^{6} B cells and were immunized IP with either GT2 or GT5 trimers, responses were analyzed 8 dpi.

D. FACS plots of germinal centers (GC) responses at 8 dpi after immunization with GT2 or GT5 (left). Quantification of CD45.2 responses in GC (right) (n=4). Experiments were performed in duplicate, one is shown. Data are presented as mean ± SD.

E. Localization of sites of mutation enrichment in the IGH of CD45.2 B cell isolated 42 days after GT5 protein immunization.

F. HCDR3 alignment of PCT64 LMCA, LMCA.JREV and consensus of mutations acquired 42 days after GT5 immunization.

G. FACS analysis of GC responses 13 days after IM immunization with either GT5 mRNA or GT5 soluble protein in PCT64$^{LMCA.JREV}$ recipient mice, quantification is on the right (n=4). Experiments were performed in triplicate, one is shown. Data are presented as mean ± SD.

H. Localization of sites of mutation enrichment in the IGH of CD45.2 B cell isolated 42 days after GT5 mRNA immunization (left). Pie chart with frequency of mutation Y117D (100% of 50 isolated IGH) (right).
