# Supplementary material for: Membrane-bound mRNA immunogens lower the threshold to activate HIV Env V2 apex-directed broadly neutralizing B cell precursors in humanized mice
Source: Immunity. 2022 Nov 8;55(11):2168–2186.e6. doi: 10.1016/j.immuni.2022.09.003 (PMC9671093; doi:10.1016/j.immuni.2022.09.003)
Supplement: Document S2. Article plus supplemental information [file mmc3.pdf]

Immunity

Membrane-bound mRNA immunogens lower the threshold to activate HIV Env V2 apex-directed broadly neutralizing B cell precursors in humanized mice

Graphical abstract

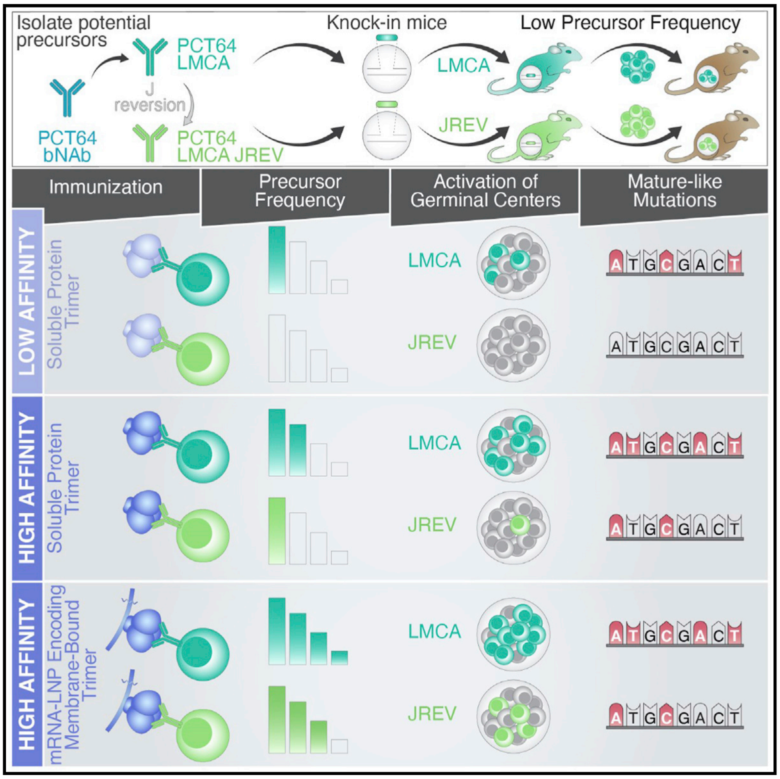

Highlights

- Generated knockin mice with B cells expressing PCT64 germline precursors
- A high-affinity GT immunogen is required to activate rare PCT64 precursors
- GT immunization induces mature PCT64-like mutations in the heavy chain
- Membrane-bound mRNA-LNP immunization lowers the activation affinity threshold

Authors

Eleonora Melzi, Jordan R. Willis, Krystal M. Ma, ..., Andrew B. Ward, William R. Schief, Facundo D. Batista

Correspondence

schief@scripps.edu (W.R.S.), fbatista1@mgh.harvard.edu (F.D.B.)

In brief

Eliciting broadly neutralizing antibodies (bnAbs) to HIV, such as the V2-Apex-targeted bnAb PCT64, is the goal of germline-targeting (GT) vaccines. Using humanized Ig knockin mouse models, Melzi et al. demonstrate the activation of rare PCT64 precursors with a high-affinity immunogen, ApexGT5. Furthermore, they find that mRNA-LNP-encoding membrane-bound ApexGT5 trimers lowers the affinity threshold for activation relative to protein immunogens.

Melzi et al., 2022, Immunity 55, 2168–2186
November 8, 2022 © 2022 The Author(s). Published by Elsevier Inc.
https://doi.org/10.1016/j.immuni.2022.09.003

CellPress

Immunity

Article

Membrane-bound mRNA immunogens lower the threshold to activate HIV Env V2 apex-directed broadly neutralizing B cell precursors in humanized mice

Eleonora Melzi,[1,10] Jordan R. Willis,[2,3,10] Krystal M. Ma,[2,3,10] Ying-Cing Lin,[1,11] Sven Kratochvil,[1,11] Zachary T. Berndsen,[4,8,11] Elise A. Landais,[2,3] Oleksandr Kalyuzhniy,[2,3] Usha Nair,[1] John Warner,[1] Jon M. Steichen,[2,3] Anton Kalyuzhniy,[2,3] Amber Le,[2,3] Simone Pecetta,[1,9] Manfredo Perez,[1] Kathrin Kirsch,[1] Stephanie R. Weldon,[1] Samantha Falcone,[5] Sunny Himansu,[5] Andrea Carfi,[5] Devin Sok,[2,3] Andrew B. Ward,[3,4] William R. Schief,[1,2,3,*] and Facundo D. Batista[1,6,7,12,*]

[1]The Ragon Institute of MGH, MIT and Harvard, Cambridge, MA 02139, USA
[2]Department of Immunology and Microbiology, The Scripps Research Institute, La Jolla, CA 92037, USA
[3]International AIDS Vaccine Initiative Neutralizing Antibody Center, the Collaboration for AIDS Vaccine Discovery (CAVD) and Scripps Consortium for HIV/AIDS Vaccine Development (CHAVD), The Scripps Research Institute, La Jolla, CA 92037, USA
[4]Department of Integrative, Structural and Computational Biology, The Scripps Research Institute, La Jolla, CA 92037, USA
[5]Moderna Inc., Cambridge, MA 02139, USA
[6]Department of Immunology, Harvard Medical School, Boston, MA, USA
[7]Department of Microbiology, Harvard Medical School, Boston, MA, USA
[8]Present Address: Biochemistry, University of Missouri, Columbia, MO 65211, USA
[9]Present address: Research and Development Centre, GlaxoSmithKline, 53100 Siena, Italy
[10]These authors contributed equally
[11]These authors contributed equally
[12]Lead contact
*Correspondence: schief@scripps.edu (W.R.S.), fbatista1@mgh.harvard.edu (F.D.B.)
https://doi.org/10.1016/j.immuni.2022.09.003

SUMMARY

Eliciting broadly neutralizing antibodies (bnAbs) is the core of HIV vaccine design. bnAbs specific to the V2-apex region of the HIV envelope acquire breadth and potency with modest somatic hypermutation, making them attractive vaccination targets. To evaluate Apex germline-targeting (ApexGT) vaccine candidates, we engineered knockin (KI) mouse models expressing the germline B cell receptor (BCR) of the bnAb PCT64. We found that high affinity of the ApexGT immunogen for PCT64-germline BCRs was necessary to specifically activate KI B cells at human physiological frequencies, recruit them to germinal centers, and select for mature bnAb mutations. Relative to protein, mRNA-encoded membrane-bound ApexGT immunization significantly increased activation and recruitment of PCT64 precursors to germinal centers and lowered their affinity threshold. We have thus developed additional models for HIV vaccine research, validated ApexGT immunogens for priming V2-apex bnAb precursors, and identified mRNA-LNP as a suitable approach to substantially improve the B cell response.

INTRODUCTION

Despite decades of research, an HIV vaccine remains elusive (Ng'uni et al., 2020), but the discovery that some HIV-infected individuals can develop broadly neutralizing antibodies (bnAbs) capable of potently neutralizing a high proportion of HIV-1 isolates has revolutionized the field (Klein et al., 2013; Flemming, 2018; Sok and Burton, 2018). These bnAbs target highly conserved regions of HIV envelope glycoprotein (Env), including the V2-apex region (Burton and Hangartner, 2016; Sok and Burton, 2018).

HIV bnAbs generally exhibit one or more traits that render elicitation by vaccination challenging: long heavy-chain third complementarity-determining regions (HCDR3s), high rates of somatic hypermutation (SHM), insertions and deletions (indels) and poly- or autoreactivity (Klein et al., 2013; Burton and Hangartner, 2016; Kelsoe and Haynes, 2017). In HIV-infected individuals, these features result from B cells co-evolving with the virus, undergoing multiple rounds of SHM and selection inside the germinal centers (GCs) in response to viral escape mutations (Doria-Rose and Landais, 2019). Although the inferred precursors of some bnAbs have affinity for Env from particular HIV isolates (Pancera et al., 2010; Liao et al., 2013; Doria-Rose et al., 2014; Andrabi et al., 2015; Gorman et al., 2016), many have no detectable affinity for Env (Xiao et al., 2009; Zhou et al., 2010; Mouquet et al., 2012; Hoot et al., 2013; Jardine et al., 2013;

 © 2022 The Author(s). Published by Elsevier Inc.
This is an open access article under the CC BY license (http://creativecommons.org/licenses/by/4.0/).

CellPress
OPEN ACCESS

Sok et al., 2013; Steichen et al., 2019), potentially explaining why immunization strategies using native Env trimers have failed (Sanders and Moore, 2017; Ng'uni et al., 2020). A germline-targeting (GT) approach using rationally designed immunogens to prime B cells encoding germline antibodies with the potential to develop into bnAbs might overcome this bottleneck (Jardine et al., 2013; Rappuoli et al., 2016; Steichen et al., 2019). After activation, B cells could be shepherded toward breadth and potency by booster immunogens increasingly resembling native Env (Jardine et al., 2013; McGuire et al., 2013; Briney et al., 2016; Escolano et al., 2016; Steichen et al., 2016; Tian et al., 2016; Stamatatos et al., 2017; Chen et al., 2021).

Pre-clinical *in vivo* models to characterize the activation and maturation of specific human B cell clones are required to reproducibly study antibody evolution induced by GT immunogens (Dosenovic et al., 2015; Jardine et al., 2015; Escolano et al., 2016; Tian et al., 2016). Mouse models expressing human Ig have proven effective in evaluating HIV immunization regimens (Ota et al., 2013; Dosenovic et al., 2015; Tian et al., 2016; Lin et al., 2018). They have been used to validate GT immunogens designed to activate precursors for CD4bs- (VRC01-class bnAbs) (Dosenovic et al., 2015; Jardine et al., 2015, 2016; Tian et al., 2016) and N332-supersite-binding bnAbs (PGT121- and BG18-class bnAbs) (Escolano et al., 2016; Steichen et al., 2016, 2019).

Previously, we reported an approach to design GT immunogens to bind precursors of HCDR3-dominant bnAbs, which triggered robust responses from BG18 precursors (Steichen et al., 2019). V2-apex directed antibodies, such as PG9, PG16, CAP256, and PCT64, are also heavily reliant on HCDR3 for neutralization and thus ideal targets to determine whether this strategy is generalizable to another Env epitope. Most V2-apex bnAbs have long ($\geq$25 residues), protruding, anionic, and often tyrosine-sulphated HCDR3 loops to penetrate the glycan shield and reach a positively charged glycopeptide epitope on the Apex of Env (Walker et al., 2009; Pancera et al., 2013; Doria-Rose et al., 2014; Andrabi et al., 2015). Notably, V2-apex bnAbs are among the most commonly identified bnAb families in patient serum samples, arise early post-infection (Walker et al., 2010; Georgiev et al., 2013; Landais et al., 2016), and require only moderate SHM, making them highly desirable targets for immunization (Moore et al., 2017). Of particular interest is PCT64; isolated from an HIV-1 subtype A-infected donor (Landais et al., 2016, 2017), PCT64 neutralizes 29% of all HIV isolates with moderate potency and up to 56% and 48% of subtype A and C viruses, respectively (Landais et al., 2017). It has an HCDR3 length of 25 amino acids and 10%–12% SHM; compared to other V2-Apex bnAbs, its HC precursors are relatively common in the human repertoire (upper limit of ~20 in 1 × 10^6 B cells) (Willis et al., 2022). Furthermore, the maturation trajectory of the PCT64 antibody line has been described in a three-year-long coevolutionary study that offers a blueprint for recapitulation (Haynes et al., 2016; Landais et al., 2017; Rantalainen et al., 2018).

Here, we developed two preclinical mouse models with B cells expressing two distinct early ancestors of PCT64 to assess protein- and mRNA-based Apex germline-targeting (ApexGT) immunogens (Willis et al., 2022). High affinity ApexGT immunogens activated rare PCT64 precursors and induced *on-track* mature PCT64-like mutations, with evolution driven primarily by the HC. Furthermore, we found that mRNA-LNP mediated *in vivo* expression of a membrane-anchored ApexGT trimer immunogen was a promising avenue for priming PCT64-like responses.

RESULTS

Generation of a PCT64 precursor knockin mouse

To study immune responses against the Apex of HIV-1 Env *in vivo*, we generated a knockin (KI) mouse with B cells bearing the least mutated common ancestor (LMCA) heavy chain (IGH) and light chain (IGK) of bnAb PCT64, which was isolated from a human donor (Landais et al., 2017). $PCT64^{LMCA}$ IGH has 99.4% germline sequence identity with a fully reverted germline V gene (VH3-15*01) and a J gene (JH6*03) containing three amino acid mutations, while the $PCT64^{LMCA}$ IGK is encoded by a fully germline V gene (Vκ3-20*01) and J gene (Jκ3*01) (Landais et al., 2017).

Using our CRISPR/Cas9 protocols (Lin et al., 2018; Wang et al., 2021), we inserted the $PCT64^{LMCA}$ IGH and IGK variable regions into their respective native murine loci and confirmed insertion by genotyping. Next, we established the frequency of cells expressing the PCT64 KI sequences by sorting and sequencing $B220^{+}$ naive peripheral B cells (Figure S1A). $PCT64^{LMCA}$ IGH was expressed by 85.7% of naive B cells (Figure S1B) and $PCT64^{LMCA}$ IGK by 86.3% (Figure S1C). Paired sequences showed that both human $PCT64^{LMCA}$ IGH and IGK could form hybrid BCRs by pairing with a variety of murine heavy chains (HCs) and light chains (LCs) (Figures S1D and S1E). To generate mice carrying the full $PCT64^{LMCA}$ antibody, we crossed PCT64 IGH ($PCT64^{LMCA-H}$) and PCT64 IGK ($PCT64^{LMCA-K}$) mice and obtained offspring where both human IGH (96%-100% of expression) and IGK (86%-90%) paired with each other (~81%) (Figures 1A, S1F, and S1G). Screening differentiation stages in the bone marrow (Figures S2A and S2B) and in the spleen (Figures S2C and S2D) confirmed that B cells underwent normal development in both the $PCT64^{LMCA-H}$ and $PCT64^{LMCA}$ mice (Hardy et al., 1991). To assess whether $PCT64^{LMCA}$ B cells express functional BCRs, we measured antigen specific binding to an ApexGT2 trimer probe (GT2 below) engineered to bind $PCT64^{LMCA}$ with moderate affinity (monovalent K_D = 167 nM) (Willis et al., 2022). Approximately 58% of peripheral blood B cells bound to GT2 in a double fluorophore staining assay (~0.04% of B cells in C57BL/6J mice were GT2 reactive); 99% of binders were found to be epitope-specific using an epitope knockout (KO) probe (GT2-KO) (Figures 1B and 1C). B cell receptor (BCR) sequencing found GT2-reactive B cells to be positive for both the human $PCT64^{LMCA}$ IGH (100%) and corresponding human $PCT64^{LMCA}$ IGK (95%) (Figure 1D). In summary, $PCT64^{LMCA}$ B cells exhibited Ig gene allelic exclusion, underwent normal development, and expressed functional paired human KI BCRs, which bound the ApexGT2 probe.

GT2 immunization activates $PCT64^{LMCA}$ B cells and generates durable GCs

Next, we tested the capacity of GT2 to activate $PCT64^{LMCA}$ B cells *in vivo*. Given the high frequency of $PCT64^{LMCA}$ B cells in our KI mouse line, we used an adoptive transfer system where 5x10^5 $CD45.2^{+}$ $PCT64^{LMCA}$ or $CD45.2^{+}$ wild-type (WT) control

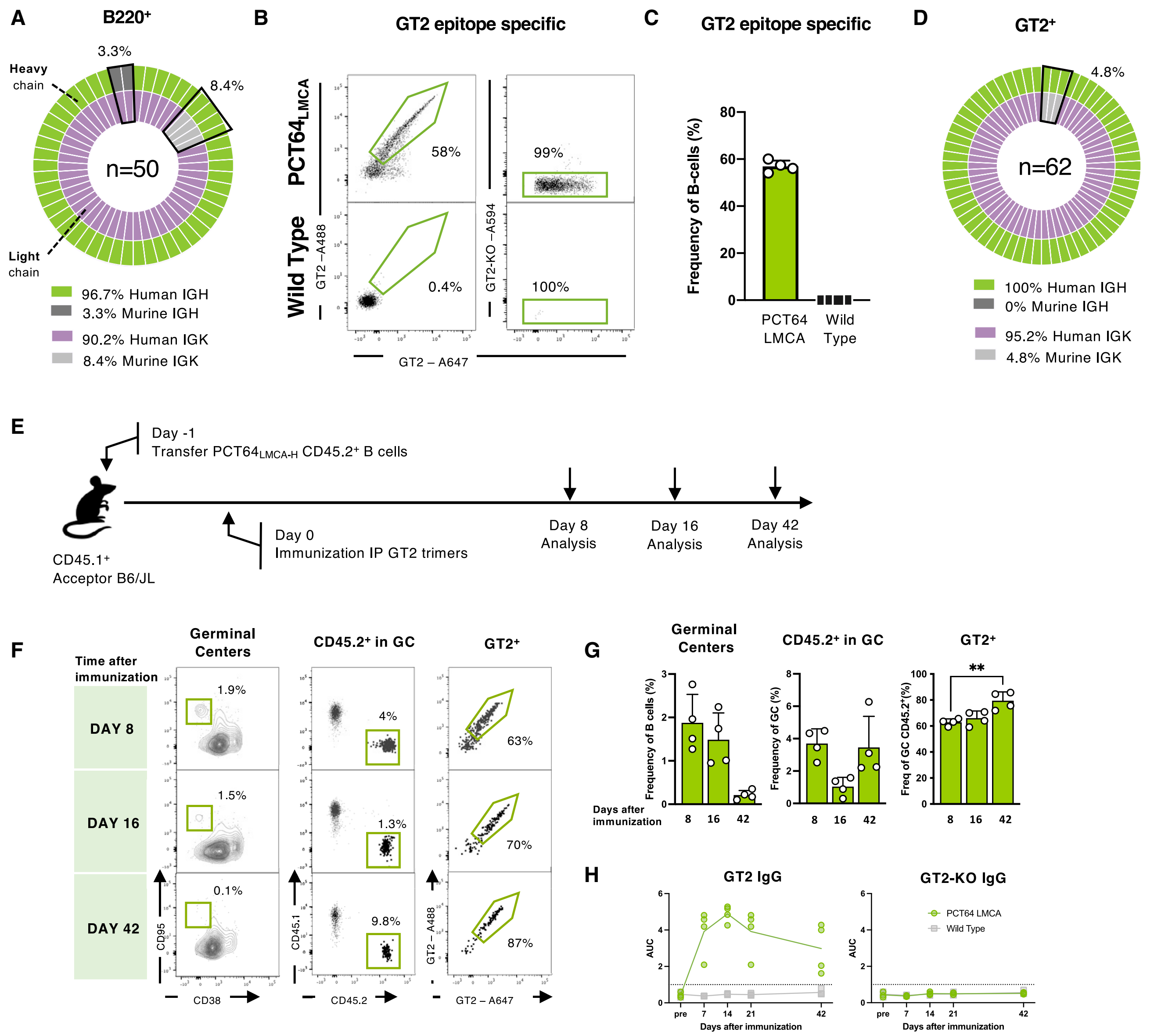

Figure 1. GT2 immunization activates PCT64 precursor B cells

(A) Human PCT64 LMCA IGH (green), murine IGH (dark gray), human PCT64 LMCA IGK (purple) and murine IGK (light gray) sequences amplified from single-cell sorted B220$^+$ naive B cells from two PCT64^{LMCA} mice. n = pairs amplified. See also Figures S1 and S2A–S2D.

(B) Representative FACS plots of epitope-specific GT2-positive and GT2-KO-negative peripheral B cells in naive PCT64^{LMCA} or C57BL/6J mice. Events were pre-gated on lymphocytes/singlets/CD4$^-$CD8$^-$F4/80$^-$Gr1$^-$/B220$^+$ B cells.

(C) GT2-specific blood peripheral B cells from PCT64^{LMCA} and WT C57BL/6J mice (n = 4). Bars are mean ± SD.

(D) Human PCT64 LMCA IGH (green), human PCT64 LMCA IGK (purple) and murine IGK (light gray) sequences from single-cell sorted GT2-specific naive B cells in PCT64^{LMCA} mice (n = 2). n = pairs amplified.

(E) Schematic of PCT64^{LMCA} B cell adoptive transfer and immunization. Experiments performed in triplicate; n = 4. A representative experiment is shown in (F–H).

(F) Representative plots of splenic B cells obtained at 8, 16, and 42 dpi with GT2 trimers. Events pre-gated on lymphocytes/singlets/live/CD4$^-$CD8$^-$F4/80$^-$Gr1$^-$/B220$^+$ B cells and represent GC, CD45.1, and CD45.2 cells in GC, and frequency of GT2$^+$ CD45.2 cells present in GC. For control groups see Figure S2E.

(G) B cell subsets responsive to GT2-immunization at 7, 16, and 42 dpi (n = 4). Left to right: total GCs, CD45.2$^+$ B cells in GCs, and GT2-binding CD45.2$^+$ B cells. Bars are mean ± SD. p values calculated by Mann-Whitney test, **$p < 0.01$. See also Figure S2E.

(H) ELISA quantification of GT2-binding (left) and GT2-KO-binding (right) serum IgG from PCT64$^{LMCA-HL}$ recipient mice versus WT C57BL/6J mice (n = 4). Line represents mean values. Area under the curve (AUC) was assessed prior to immunization with GT2 trimers and at 7, 14, 21, and 42 dpi.

CellPress
OPEN ACCESS

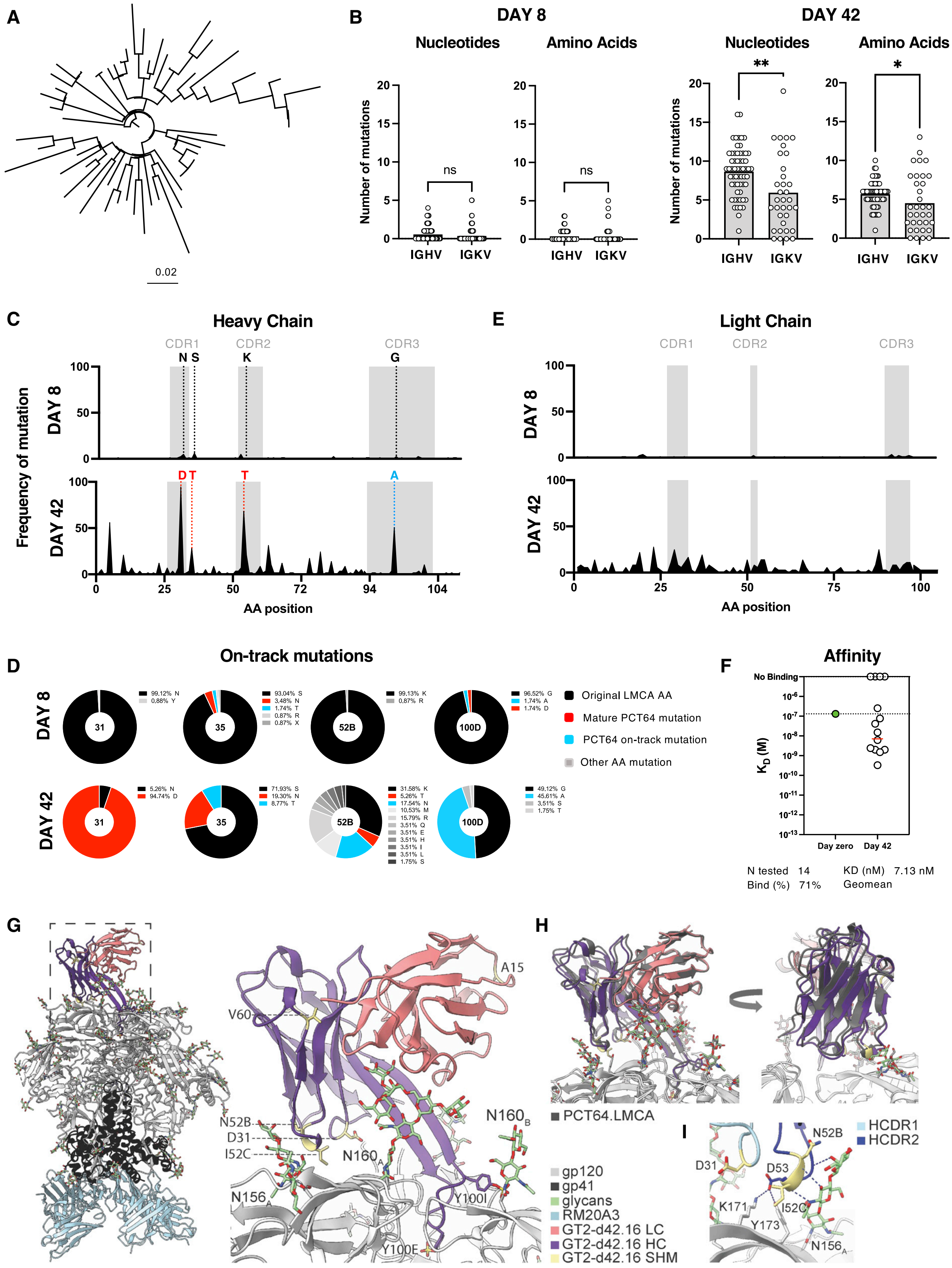

(legend on next page)

B cells were transferred into congenic CD45.1$^+$ C57BL/6J recipient mice (Figure 1E) to decrease precursor frequency and facilitate the tracking of PCT64^{LMCA} responses. We immunized mice 24 h after adoptive transfer with either GT2 or BG505-MD39 SOSIP trimers formulated in Sigma adjuvant system (Sigma) intraperitoneally (i.p.)—an established route for SOSIP immunogens (Escolano et al., 2016; Steichen et al., 2016)—and measured responses in the spleen (Figures 1E–1G and S2E). GC responses (GC; CD95^{hi}CD38^{lo}) were detected 8 days post immunization (dpi) in all groups; CD45.2$^+$ PCT64^{LMCA} B cells represented on average 3.7% of the activated GC B cells in GT2 immunized mice at this timepoint, and 63% were GT2 specific (Figure 1F). CD45.2$^+$ cells were not detected in GCs in the BG505-MD39 SOSIP trimer cohort or in mice that received WT cells (Figure S2E). At 16 and 42 dpi, CD45.2$^+$ GC responses were still ongoing. The GT2 specificity of CD45.2$^+$ GC cells increased from 8 to 42 dpi (from 62.8% to 79.4%, $p = 0.0038$), suggesting ongoing selection expanding clones with higher affinity for GT2 (Figure 1G). Furthermore, epitope-specific IgG titers were detectable at 7 dpi in PCT64^{LMCA} recipient mice and gradually declined after a peak at 14 dpi (Figure 1H). WT mice immunized with GT2 trimers failed to develop epitope-specific IgG antibodies. No off-target antibody responses against epitope-deficient GT2 trimers (GT2-KO) were detected in either group (Figure 1H), indicating that serum IgG antibody responses were due to the transferred PCT64^{LMCA} B cells.

Overall, PCT64 precursors were successfully activated by GT2 immunization, formed sustained GC reactions, and generated epitope-specific IgG responses.

GT2-primed PCT64^{LMCA} BCR heavy chains acquire bnAb-like mutations

To determine whether PCT64^{LMCA} B cells underwent SHM and accumulated PCT64-like mutations after GT2-trimer immunization, we sorted class-switched GT2$^+$ PCT64^{LMCA} B cells at 8 and 42 dpi for single-cell BCR sequencing. Tracing lineage evolution of the PCT64^{LMCA} IGH highlighted broad diversification at 42 dpi (Figure 2A). SHM was minimal in the PCT64^{LMCA} IGH and IGK V-region at 8 dpi but increased over time (Figure 2B). At 42 dpi, there was significantly higher SHM in the IGH V region (8.6 nucleotides [nt]/5.6 amino acids [aa]) than in the corresponding IGK V region (5.9 nt/4.5 aa) whether comparing nt ($p = 0.0013$) or aa ($p = 0.037$) (Figure 2B). Mutations accumulated over time in recurring positions in the HCDR1, HCDR2, and HCDR3 (Figure 2C). Substitutions at these sites were enriched for aa present in the mature PCT64 bnAb (V-region position 31, 35, and 52B) and for aa present in early PCT64-line isolates (in HCDR3 position 100D) (Figure 2D) (Landais et al., 2017). N31D, which is also present in the mature PCT64 bnAb (Landais et al., 2017), was acquired in 98% of the isolated HC sequences, indicating positive selection. While enriched mutations in the HC V region were likely facilitated by activation-induced deaminase (AID) binding sites, no underlying AID hotspot was identified for the HCDR3 mutation (100D) (Figure S2F).

In contrast, no mutations were enriched in the PCT64^{LMCA} IGK (Figure 2E), suggesting an HC-driven immunogen interaction. To confirm this SHM drove antibody maturation, we expressed 14 representative 42 dpi Fabs and quantified their affinity for GT2. Of the 10 Fabs with detectable affinity for GT2, 9 showed increased affinity over the PCT64^{LMCA} Fab (K_D, 130 nM). The geomean K_D for all 10 Fabs (4.3 nM) indicated a 30-fold improvement; one Fab exhibited a 394-fold affinity gain over the native PCT64^{LMCA} Fab. (Figure 2F).

To interrogate the interaction between the acquired mutations and GT2, we determined the cryo-EM structure of GT2 in complex with a high affinity day 42 Fab (GT2-d42.16) (Figures S3A–S3C). The Env base-binding Fab RM20A3 was included to improve particle angular distribution. Our ~3.5 Å-resolution reconstruction (Figures S3D–S3G) allowed us to build atomic models of the complex (Figure 2G), which confirmed that GT2-d42.16 adopts a nearly identical structure and angle of approach to PCT64^{LMCA} (Figure 2H) with the characteristic elongated and anionic HCDR3 beta hairpin loop that extends inward toward the 3-fold axis of the trimer apex and extensive engagement with positively charged residues in the V1/V2 loop and the glycan at N160gp120$_A$ (Figures 2G, S3H, and S3I). Although the map resolution is lower due to inherent flexibility in the distal regions of the Fab outside of the paratope (Figure S3C), docking of the high-resolution PCT64^{LMCA} crystal structure allowed us to reliably identify the sites of SHM acquired during affinity maturation (Figure 2H). The only positions of SHM within the paratope are in the HCDR1 and 2 domains, which are responsible for engaging/accommodating the glycan at N156gp120$_A$ as well as the C strand of V2 (Figure 2I) (Willis et al., 2022). Although the N156gp120$_A$ glycan does not engage in any specific sidechain contacts, it forms several backbone hydrogen-bonds (H-bonds) (Figure 2I) that could be enhanced by the HCDR1 and/or HCDR2 mutations by stabilizing the small helical turn in the HCDR2. Both the K52$_b$N and T52$_c$I mutations are present in early PCT64 lineage members such as PCT64.13B, although both sites were

Figure 2. A single GT2 prime induces antibodies with mature PCT64-like mutations

Antigen-specific splenic CD95$^+$CD38^{low} CD45.2$^+$ PCT64^{LMCA} B cells sorted at 8 and 42 dpi for single-cell BCR sequencing.

(A) Phylogenetic trees of PCT64^{LMCA} IGH aa at 42 dpi.

(B) Total nt (left) and aa (right) mutations in PCT64^{LMCA} IGHV and IGKV at 8 and 42 dpi. p values calculated by Mann-Whitney test: **$p < 0.01$, *$p < 0.05$.

(C) HC mutation frequencies per residue observed at 8 (n = 93) and 42 (n = 57) dpi. HCDRs highlighted in gray. Red = present in mature PCT64; blue = present in early PCT64 isolates. aa positions 31, 35, 52B, and 100D analyzed in D.

(D) Distribution of selected PCT64^{LMCA} B cell HC aa mutations in positions 31, 35, 52B, and 1 at 8 and 42 dpi. Red = present in mature PCT64; blue = present in early PCT64 isolates; black = original LMCA aa; gray = all other mutations. See also Figure S2F.

(E) Frequencies of LC mutations at each residue 42 dpi (n = 39). LCDRs are highlighted in gray.

(F) SPR affinity measurement against GT2 for 14 antibodies isolated at 42 dpi (white, right) compared with PCT64^{LMCA} (green, left).

(G) Full cryo-EM structure of ApexGT2 in complex with GT2-d42.16 and RM20A3 Fabs and close-up of the epitope/paratope region with sites of SHM in yellow. See also Figure S3.

(H) Structures of GT2-d42.16 and PCT64^{LMCA} (dark gray) overlayed showing an identical angle of approach relative to their respective ApexGT trimers.

(I) Close up of the HCDR1 (light blue) and 2 domains (dark blue) with H-bonds between the N156gp120A glycan and gp120 residues shown as dashed lines.

CellPress
OPEN ACCESS

further mutated in the mature PCT64 bnAbs (Landais et al., 2017). The N31D mutation, located near the interface with the C strand, is present in the majority of mature PCT64 lineage members and could contribute to a more favorable electrostatic interaction with the positively charged apex. In sum, GT2 immunization could successfully initiate PCT64^{LMCA} maturation toward a higher affinity and mature-PCT64-like antibody.

PCT64 precursor responses to GT2 are driven by the heavy chain

In Apex-directed bnAbs, the HCDR3 is a major binding determinant (Pancera et al., 2010; Pejchal et al., 2010; McLellan et al., 2011; Julien et al., 2013; Andrabi et al., 2015). Mammalian display directed evolution was used to engineer the GT2 trimer to target the PCT64^{LMCA} HC, similar to the strategy used for targeting N332-dependent bnAbs (Steichen et al., 2019). To validate the specificity of the GT2 immunogen for PCT64 IGH, we used a PCT64^{LMCA} HC-only mouse model (PCT64^{LMCA-H}) in which the human PCT64^{LMCA} IGH pairs with native murine LCs.

10x Genomics single-cell BCR sequencing revealed that 82.6% of naive splenic B cells in PCT64^{LMCA-H} heterozygous mice expressed the PCT64^{LMCA} IGH sequence (Figure 3A). PCT64^{LMCA} IGH paired with a wide variety of murine IGK V genes, but there was evidence of selectivity: the most frequent pairings were with IGKV1-135 (28.8% of paired IGKV genes), V2-137 (7.74%), V2-109 (4.94%), and V1-110 (4.81%) (Figure S4A). In contrast, IGK V genes were more evenly distributed in the fully murine BCRs, with the most frequent IGK V gene (V1-110) at only 5.03% (Figure S4B).

Next, we quantified antigen-specific binding to GT2 trimer probes in naive PCT64^{LMCA-H} mice. Approximately 32% of peripheral blood B cells bound the GT2 probe in a double fluorophore staining assay (Figure 3B); binders consisted of 100% human PCT64^{LMCA} IGH paired with various murine LCs (Figure 3C); hybrid BCRs were thus capable of GT2 binding.

Immunization with GT2 trimers as above activated CD45.2^{+} PCT64^{LMCA-H} B cells and generated long-lasting GCs (Figure S5A). To determine the contributions of IGK to affinity maturation, we single-cell sorted class-switched, GT2-specific, CD45.2^{+} B cells at 8 and 42 dpi for BCR sequencing. All isolated HC were derived from the KI PCT64^{LMCA} but paired with a variety of murine LCs at both timepoints, indicating ongoing multiclonal selection (Figures 3D and 3E). Some IGK V genes were enriched during GC selection: ~28% of the isolated LCs in the GC at 8 dpi expressed IGK V2-109 (which represented 4.9% of the IGK V genes paired with PCT64^{LMCA-H} in the naive repertoire) (Figures 3D and 3E). Similarly, other initially low-frequency V genes, such as V4-74 (0.23% in the naive repertoire), V14-111 (1.59%), and V12-44 (0.75%), were enriched over time (Figures 3D and 3E).

Due to this enrichment, we measured the affinities of PCT64^{LMCA} IGH paired with different naive murine IGK (Figure 3F). Hybrid antibodies isolated from naive PCT64^{LMCA-H} mice, which constituted a polyclonal population generated through LC variability, had affinities (K_Ds) from $1.6x10^{-6}$ to $2.5x10^{-8}$ M. Some IGK V genes, such as IGKV2-109 and IGKV12-44 (both enriched in the GC), had up to 2.6-fold higher affinity for GT2 than PCT64^{LMCA} paired with its natural human LC. In contrast, lower affinity was found with IGKV1-135 and IGKV2-137, which were far less frequent in the GC than in the naive repertoire (Figure 3F).

We then investigated whether IGH could acquire PCT64-like mutations in the absence of the human LC. SHM and diversification increased over time (Figure S5B); by 8 dpi, human VH3-15 acquired an average of n = 1.2 ± 1.6 nt mutations (0.89 ± 1.1 aa) (Figure S5C) and reached an average of n = 7.7 ± 2.4 nt (4.9 ± 1.9 aa) by day 42 (Figure S5C), similar to the rate observed in the full PCT64^{LMCA} KI model. Enriched residues in PCT64^{LMCA-H} IGH matched those identified for PCT64^{LMCA} (Figures 3G, 3H, S5D, and S5E), and the aa mutations at these sites included previously identified PCT64-like mutations in positions 31, 35, 52_B, and 100_D (Figures 3G and 3H), suggesting that IGH evolution in response to GT2 trimers is both consistent and independent of the LC. This demonstrated that B cells bearing PCT64^{LMCA} HC in conjunction with diverse LCs could respond with great specificity to GT2 immunization.

A high affinity immunogen is required to activate rare PCT64 precursors

Human repertoire data suggest that a suitable PCT64-immunogen needs to reproducibly trigger B cells at frequencies lower than ~20 precursors per 10^6 (Willis et al., 2022). To evaluate the capacity of GT2 to activate PCT64 precursors at human physiological frequencies, we calculated the frequencies of GT2^{+} PCT64^{LMCA-H} B cells in the spleens of recipient mice at the time of immunization, 24 h after the adoptive transfer of $5x10^5$, $1x10^5$, or $5x10^4$ CD45.2^{+} B cells (Figures 4A and 4B). The resulting GT2-specific CD45.2^{+} B cell frequencies were 100, 20, or 10 per 10^6 splenic B cells, respectively (Figures 4C and 4D). Responses in immunized recipient mice with defined numbers of PCT64^{LMCA} B cells were analyzed 8 dpi (Figure 4E). While PCT64^{LMCA} frequency did not affect total GC size, it did affect the proportion of CD45.2^{+} cells in GCs, from 1.2% CD45.2^{+} at 100:10^6 to barely 0.2% at 10:10^6 (Figures 4F and 4G).

Immunogen affinity is key to rare B cell activation (Dosenovic et al., 2015; Sok et al., 2016; Tian et al., 2016; Abbott et al., 2018). To assess the effect of affinity on activation, we immunized mice with the range of PCT64 precursors defined above with ApexGT5 (GT5 below), an ApexGT trimer with higher affinity for PCT64^{LMCA} (K_D, 66 nM compared to 167 nM for GT2) (Figure 4H) (Willis et al., 2022), and compared responses at 8 dpi to GT2 (Figure 4E). We observed a relative increase in CD45.2^{+} B cell recruitment to GCs in all GT5-immunized groups. The gap was most pronounced at the lowest precursor frequency (10:10^6) in which GT5 immunization activated 20 times more PCT64^{LMCA} B cells than GT2 (4% CD45.2 versus 0.2%) (Figures 4I and 4J). GT5-specific responses were dominated by CD45.2^{+} B cells (>90%) at all tested precursor frequencies; in contrast, GT2-specific responses were directly proportional to the initial precursor number and were outnumbered in the GC by competitor CD45.1^{+} murine B cells (Figures S5F and S5G).

To assess GT5 efficacy at even more stringent frequencies, we established cohorts with 100, 10, or 1 precursor(s) per 10^6. While GC responses decreased over time, CD45.2^{+} cells persisted in the GC from 8 until 42 dpi at both 100 and 10 per 10^6 (Figures 4K and 4L). However, at 1 per 10^6, only a weak CD45.2^{+} response was generated at 8 dpi, and none was

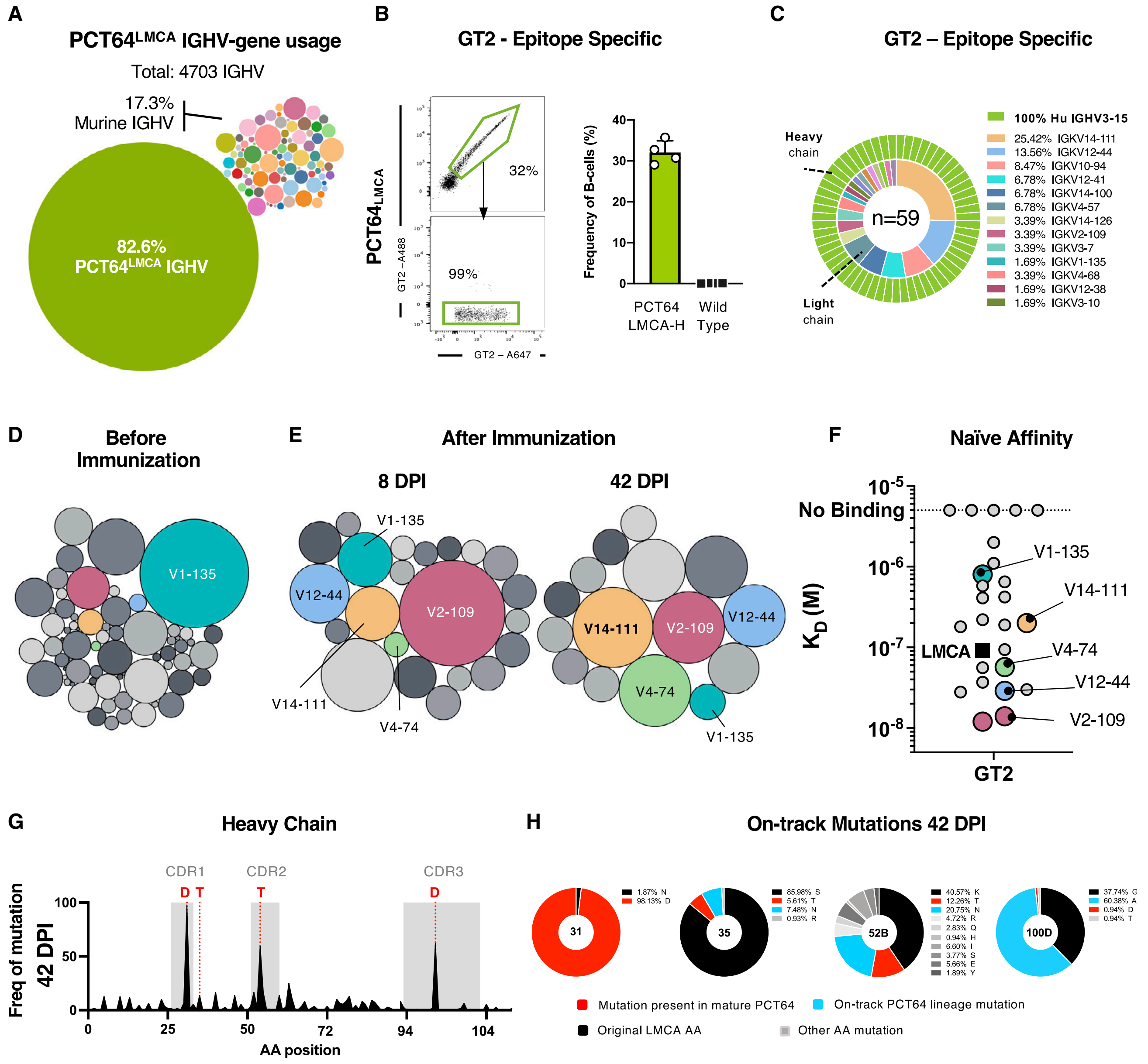

Figure 3. PCT64 precursor responses to GT2 are driven by the heavy chain

(A) 10x Genomics single-cell BCR sequences from 4703 splenic B cells from a naive PCT64^{LMCA-H} mouse. Relative bubble size indicates IGHV gene frequency. Human PCT64 IGHV gene frequency in green (82.6%). Murine IGHV genes in various colors.

(B) Left: representative fluorescence-activated cell sorting (FACS) plot of GT2-binding and GT2-KO-negative peripheral B cells in naive PCT64^{LMCA-H} (n = 4). Events were pre-gated on lymphocytes/singlets/CD4$^-$CD8$^-$F4/80$^-$Gr1$^-$/B220$^+$ B cells. Right: quantification of GT2-specific blood peripheral B cells from PCT64^{LMCA-H} and C57BL/6J WT mice. Bars are mean ± SD.

(C) Paired human PCT64^{LMCA} IGH (green) and murine IGK (variable) sequences amplified from single-cell sorted GT2-specific naive B cells from PCT64^{LMCA-H} mice. n = pairs amplified.

(D) Murine IGK V genes paired with human PCT64 IGH in a naive PCT64^{LMCA-H} mouse (n = 3,888). Relative bubble size indicates frequency of IGK V gene usage. Relevant IGK V genes marked in color and analyzed in (E) and (F). See also Figure S4.

(E) Murine IGK V genes paired with human PCT64 IGH isolated from GCs at 8 and 42 dpi. Some frequently enriched IGK V genes are highlighted: V2-109 (red), V12-44 (blue), V14-111 (yellow), V4-74 (light green), and V1-135 (teal). See also Figure S5A.

(F) SPR affinity against GT2 for 23 antibodies with various murine IGKV pairings isolated from naive PCT64^{LMCA-H} mice at 42 dpi, compared to human PCT64^{LMCA} (black, square).

(G) Frequencies of IGH aa mutations per residue 42 dpi. HCDRs are boxed in gray. Red = key mutations present in mature PCT64. aa positions 31, 35, 52B, and 100D analyzed in (J). See also Figures S5B–S5E.

(H) Distribution of select PCT64^{LMCA} B cell IGH aa mutations in positions 31, 35, 52B, and 100D at 8 and 42 dpi. Red = present in mature PCT64; blue = present in early PCT64 isolates; black = original LMCA aa; gray = all others.

CellPress
OPEN ACCESS

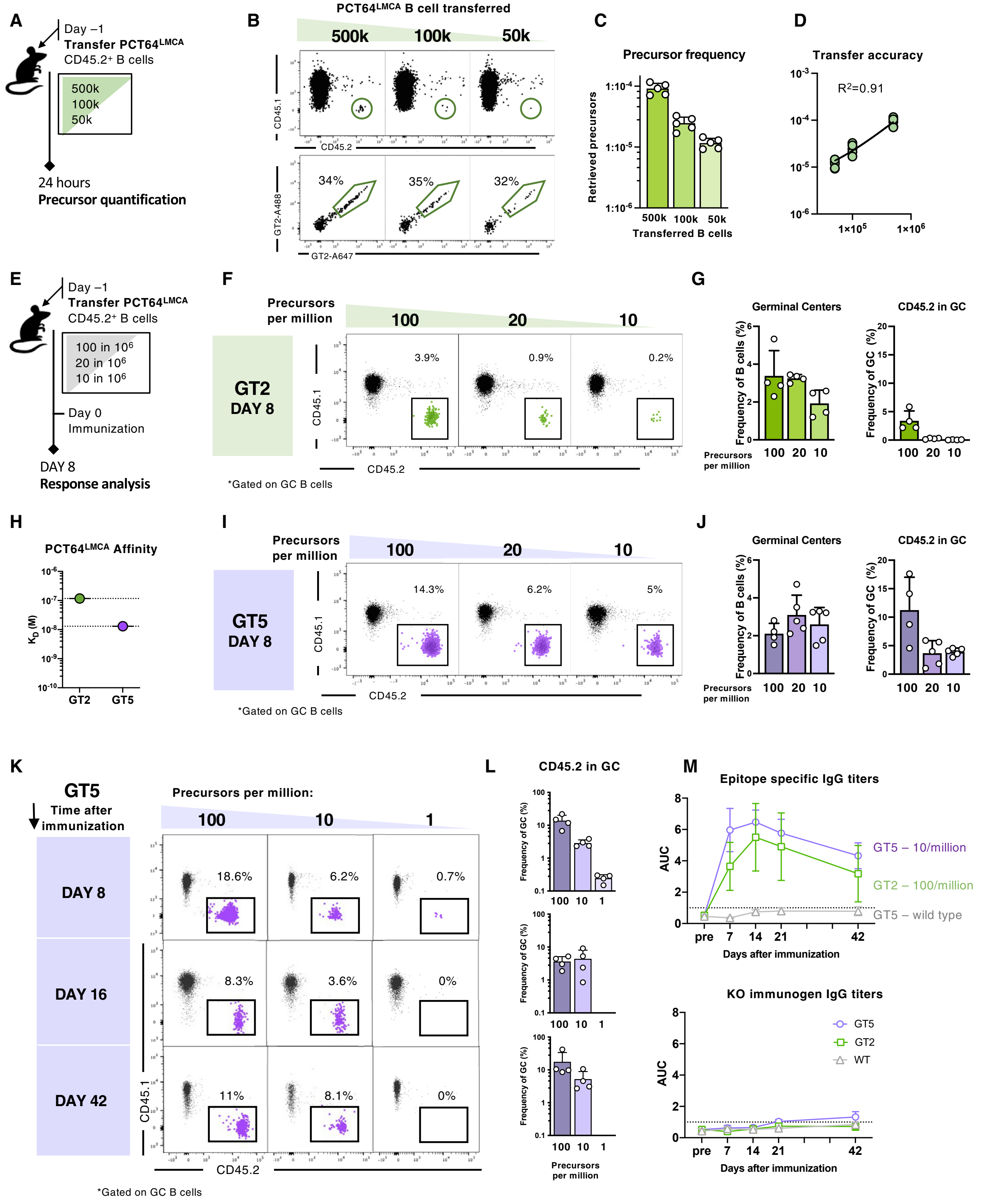

(legend on next page)

detected by 42 dpi. Epitope-specific IgG responses were detected by ELISA from 7 to 42 dpi in recipients of 10 PCT64^{LMCA} B cells per 10^6; GT2-immunization produced comparable titers in recipients of 100 PCT64^{LMCA} B cells per 10^6. WT control mice immunized with GT5 did not develop detectable epitope-specific IgG responses, and no off-target response was detected in any group (Figure 4M). Thus, the higher affinity GT5 protein trimer could specifically activate PCT64 precursors at frequencies approaching the estimated human physiological range.

GT5 immunization induces mature PCT64-like mutations in IGH

To determine whether GT5 could induce on-track SHM in PCT64 precursors, we sorted CD45.2$^+$ GT5$^+$IgG1$^+$ B cells at 42 dpi for single-cell BCR sequencing (Figure S5H). Diversification occurred in both PCT64^{LMCA} IGH and IGK sequences (Figure 5A); the average number of mutations acquired in the V-region was 6.6 nt/4.4 aa for IGH and 6.2 nt/4.6 aa for IGK (Figure 5B). From this antibody library, we expressed 15 representative Fabs; 13 of 15 (87%) showed detectable binding to GT5 (K_D<10 μM), with a geomean affinity of 0.59 nM among binders, representing an ~110-fold increase over the affinity of the PCT64^{LMCA} (66 nM). We also produced 13 Fabs with PCT64^{LMCA} IGH paired with murine IGK chains; 9 of 13 (69%) had detectable affinity, and the geomean affinity among binders was 0.29 nM, an even larger increase over PCT64^{LMCA} (Figure 5C).

We compared mutations acquired by IGH in our model with those from human PCT64 precursors isolated 8–35 months post-infection to determine whether the trajectory of antibody evolution was similar (Landais et al., 2017). IGH sequences isolated at 42 dpi carried an average of 3–4 PCT64-like mutations and a peak of 7 PCT64-like mutations (Figure 5D). Total mutations and PCT64-donor-like mutations were positively correlated (R^2 = 0.4869). GT5 immunization generated a broader, more diverse repertoire than GT2; aa mutations in IGH were distributed across more numerous sites, though relatively less enriched (Figures 5E and 5F). This plasticity promoted PCT64-like mutations at 3 sites in HCDR1 (positions 28, 31, and 35), 3 sites in HCDR2 (positions 52, 52B, and 52C), and 4 sites in HCDR3 (positions 92, 97, 100C, and 100D) (Figure 5F). As with GT2, no enrichment site was identified in the LC.

To assess whether SHM acquired from priming conferred a degree of neutralization, five GT2- and five GT5-induced day 42 mAbs were tested against a series of WT and modified (with ApexGT mutations) HIV pseudoviruses (PSVs) based on isolates neutralized by PCT64 (Figure 5G) (Landais et al., 2017). None of the day 42 mAbs could neutralize the WT PSVs, but some showed partial neutralization of PSVs with ApexGT mutations, especially PSVs with the K169R mutation (PSVs GT5-V2B and GT5-N167) (Figure 5G). This is consistent with the binding mode of PCT64^{LMCA} (Willis et al., 2022) and day 42 Fabs (Figures 2G–2K and 5H–5L), which includes strong electrostatic interactions between their acidic residues and R169 of ApexGTs. To clarify the molecular details of GT5-induced SHM, we determined the cryo-EM structure of GT5 in complex with a high-affinity day 42 Fab (GT5-d42.16) (Figures 5H and S6A–S6G). Despite positional differences in the location of SHM sites (magenta), no structural differences were detected between GT5-d42.16 and either PCT64^{LMCA} or GT2-d42.16 (Figures 5H and S6H–S6M). GT5-d42.16 has five more sites of SHM than GT2-d42.16 while both Fabs present SHM in the HCDR2 domain, which is responsible for engaging/accommodating the N156gp120A glycan and the C strand of V2 (Figures 5H and 5I). The glycan at N156 engages in multiple backbone H-bonds with GT5-d42.16, while E53 forms an H-bond with GT5 residue K171, as in GT2-d42.16. Notably, the D53E mutation is present in the vast majority of PCT64 lineage members. Unlike GT2-d42.16, GT5-d42.16 has one site of SHM in the HCDR3 domain, T93 N, which is located at the very beginning of the loop near the N160gp120$_A$ glycan binding pocket and is also found in the PCT64.35M bnAb lineage. All remaining mutations in the HC are located outside the paratope and thus not directly involved in affinity maturation. GT5-d42.16 also has several LC mutations, with one, S31I, located near the N160gp120$_A$ glycan binding interface in the LCDR1 domain (Figure 5H). However, this residue converges on either an Asn or Asp in all the mature PCT64 antibodies.

At the immunogen level, the only difference between GT2 and GT5 is the identity of loop2B (Figure 5H). The GT5 loop2B has the N187 glycan knocked out and includes a mutation to a bulky tryptophan residue at position 188 at the tip of the loop, among other mutations. Using difference mapping, we found that the removal of the N187 glycan creates a hole in the density

Figure 4. High affinity GT5 immunogen activates PCT64 precursors at physiological frequencies

(A) Schematic of adoptive transfer model to calibrate PCT64^{LMCA} B cell frequencies. Experiments were performed in duplicate with one presented; n = 5.
(B) Gating strategy for titration of cell transfer model.
(C) Precursor frequencies (y axis) corresponding to number of B cells transferred (x axis). Bars are mean ± SD.
(D) Analysis of linearity of CD45.2 PCT64^{LMCA} B cells recovered 24 h post transfer.
(E) Schematic of immunizations performed at precursor frequencies of 100, 20, and 10 per 10^6. Experiments were performed in triplicate with one presented; n = 4.
(F) Representative FACS plots at 8 dpi with GT, showing CD45.2 GC B cell responses at precursor frequency of 100, 20, and 10 per 10^6 of B cells.
(G) Quantification of GC B cells and CD45.2$^+$ PCT64^{LMCA} cells present in GC at 8 dpi with GT2. Bars are mean ± SD.
(H) SPR of PCT64^{LMCA} for GT2 and GT5 trimers.
(I) Representative FACS plots 8 dpi with GT5, showing CD45.2 GC B cell responses at precursor frequency of 100, 20, and 10 per 10^6 of B cells.
(J) Quantification of GC cells and CD45.2$^+$ PCT64^{LMCA} cells present in GC at 8 dpi with GT5 at decreasing precursor frequencies (n = 4 and n = 5 for 10:10^6). Bars are mean ± SD. See also Figures S5F and S5G.
(K) Representative FACS plots at 8, 16, and 42 dpi with GT5, showing CD45.2 GC B cell responses at precursor frequency of 100, 10, and 1 per 10^6 B cells (n = 4).
(L) Quantification of K. Data are represented as mean ± SD.
(M) ELISA quantification of GT2-/GT5-binding (top) and GT2-/GT5-KO-binding (bottom) serum IgG from PCT64^{LMCA} recipient mice compared to WT C57BL/6J mice (n = 4). AUC was assessed prior to immunization by GT2 or GT5 and at 7, 14, 21, and 42 dpi. Points are mean ± SD.

CellPress
OPEN ACCESS

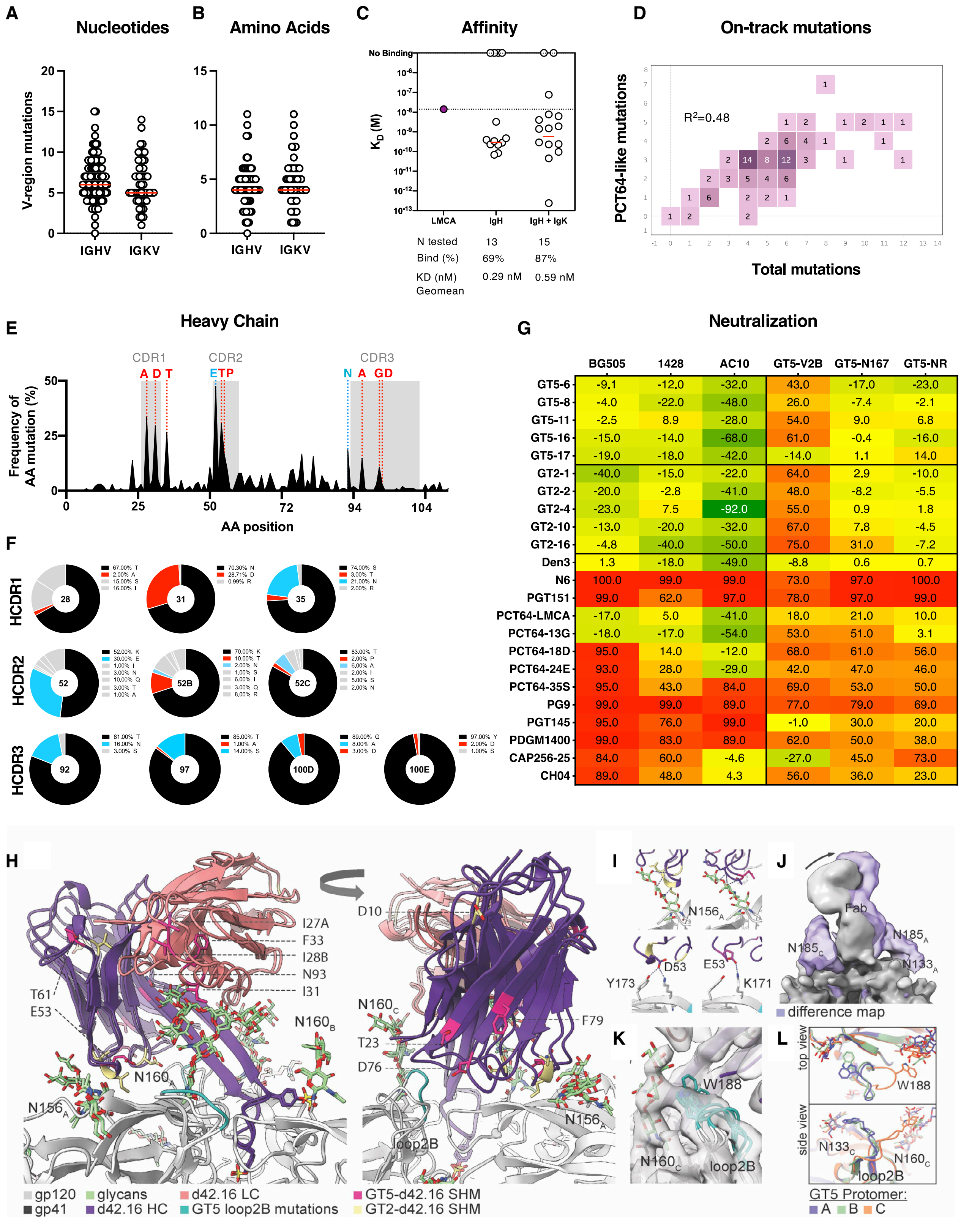

(legend on next page)

surrounding the PCT64 binding site that results in a slight change in the average binding angle of the Fab, presumably by relieving steric restrictions from the glycan (Figure 5J), which is in line with the surface plasmon resonance (SPR) results showing a slightly faster on and off rate of PCT64^{LMCA} for GT5 (Willis et al., 2022). The conformation of the loop is also affected, especially on protomer C, where it is folded inward toward the Fab and N160gp120$_C$ glycan (Figures 5K and 5L). Although W188 does not appear to interact with the GT5-d42.16 HCDR3, there is clear electron microscopy (EM) map density extending from the tip of the loop to the N160 glycan resulting from the W188 residue, suggesting it could be stabilizing the glycan, and, in turn, its interactions with the Fab (Figure 5K). Thus, immunization with GT5 can induce affinity maturation in rare PCT64 precursors and support the acquisition of PCT64-like mutations that neutralize autologous virus.

mRNA-LNP membrane-bound GT5 trimers potently activate PCT64 precursors

Nucleoside-modified mRNA vaccines for SARS-CoV-2 have proven safe and highly effective in humans (Baden et al., 2021; Thomas et al., 2021). Thus, GT5 was further developed as a membrane-bound trimer with appropriate antigenic profile expressed from DNA or mRNA (Willis et al., 2022). As human vaccines are frequently administered intramuscularly (IM) (Zhang et al., 2015), we first assessed IM GT5 protein trimer delivery. We established PCT64^{LMCA} at 10 per 10^6 B cells in recipient mice, immunized IM with GT5 in Sigma adjuvant, and measured the response in the inguinal lymph nodes (LN) at 13, 28, and 42 dpi (Figure 6A). GT5-specific CD45.2$^+$ B cells were undetectable in 13 dpi GCs and very infrequent at later timepoints (Figures 6B and 6C). To assess whether B cell responses could have occurred at other sites, we quantified GT5-specific IgG serum by ELISA but retrieved no detectable titers (Figure 6D). GT5 protein trimers may, therefore, not be suited for IM delivery in mice.

We then evaluated immune responses to mRNA-mediated expression of membrane-bound GT5 trimers. Recipient mice (10:10^6), which were immunized IM with a single dose of mRNA-LNP encoding GT5, had large GCs and high recruitment of CD45.2 PCT64^{LMCA} B cells at 13 dpi (Figures 6E and 6F). PCT64^{LMCA} B cells were maintained in GCs up to 28 and 42 dpi, and GT5-specificity increased from 60% to 90% of CD45.2 GC B cells over time (Figures 6E and 6F). ELISA of serum IgG titers found that IM mRNA-LNP immunization induced long-lasting, GT5-specific antibodies (Figure 6G).

Given the magnitude of the response to mRNA immunization, we tested whether GT5-mRNA could generate consistent responses after a 10-fold precursor reduction to 1 per 10^6 B cells (Figures 6H and 6I). Even at this extremely rare starting frequency, PCT64^{LMCA} averaged 4% of GC B cells at 13 dpi (in comparison to 9.9% at 10:10^6) and expanded to 26.6% at 42 dpi (Figure 6I). To confirm that mRNA-LNP GT5 maintained the capacity to induce PCT64-like mutations, we performed BCR sequencing of class switched CD45.2$^+$GT5$^+$ B cells at 42 dpi. Isolated IGHV genes acquired an average of 7 nt/5 aa mutations (Figure 6J). Enriched sites occurred in similar positions previously identified with protein trimer immunization (Figure 6K).

Thus, mRNA-LNP-encoded GT5 trimers activated ultra-rare PCT64 precursors more potently than soluble protein trimers and induced the acquisition of similar bnAb-like mutations.

mRNA-LNP-encoded GT5 trimers lower the precursor activation affinity threshold

GT5 was engineered using our mammalian display bootstrapping approach, with GT mutations identified first against more mature-like antibodies and then modified to increase the immunogen's affinity for progressively more germline-like antibodies (Steichen et al., 2016). The PCT64^{LMCA} sequence, isolated from the human donor early after infection, has some SHM in IGH—three aa mutations in the J gene and two aa from the full-length D gene (Figure 7A). To test the capacity of the GT immunogens to activate more reverted germline IGHs, we generated a second mouse model where the J gene is fully reverted, PCT64$^{LMCA.JREV-H}$ (Figures S7A and S7B) (Willis et al., 2022). Genotyping and single-cell BCR sequencing confirmed that the PCT64$^{LMCA.JREV-H}$ sequence was expressed by ~95% of the B cell repertoire (Figure S7B). Through crossing with

Figure 5. GT5 immunization induces key PCT64-like mutations in IGH

(A) Quantification of total nt mutations in PCT64^{LMCA} IGH and IGK V genes at 42 dpi. Red line = mean. See also Figure S5H.

(B) Quantification of total aa mutations in PCT64^{LMCA} IGH and IGK V genes at 42 dpi. Red line = mean.

(C) SPR affinity measurement of antibodies isolated at 42 dpi with GT5 from mice presenting a PCT64 IGH (left) or IGH + IGK (right) (10 precursors per 10^6).

(D) PCT64-like aa mutations in PCT64^{LMCA} IGHs isolated at 42 dpi. Numbers in each square indicate sequences sharing the total aa mutations (x axis) and the PCT64-like aa mutations (y axis).

(E) Frequencies of HC aa mutations per residue at 42 dpi. HCDRs boxed in gray. Red = present in mature PCT64; blue = present in early PCT64 isolates.

(F) Frequency of selected PCT64^{LMCA} B cell HC aa mutations at 42 dpi in HCDR1 positions 28, 31, and 35; in HCDR2 positions 52, 52B, and 52C; in HCDR3 positions 92, 97, 100D, and 100E. Red = present in mature PCT64; blue = present in early PCT64 isolates; black = original PCT64^{LMCA} aa; gray = all others.

(G) Neutralization assay of selected PCT64^{LMCA} 42 dpi mAbs. The five highest affinity mAbs elicited by ApexGT2 or GT5 were evaluated in addition to PCT64 lineage members and other V2 apex bnAbs. GT5-V2B, BG505.ApexGT5 PSV with loopV2B reverted to BG505 WT; GT5-N167, BG505.ApexGT5 PSV with N167D (BG505 WT has D167); GT5-NR, BG505.ApexGT5 PSV with N167D and R169K (BG505 WT has D167 and K169). Numbers indicate the percentage of neutralization at 10 ug/mL.

(H) Cryo-electron microscopy (cryo-EM) structure of ApexGT5 in complex with GT5-d42.16 Fab overlayed with the structure of ApexGT2 in complex with GT2-d42.16 Fab. Sites of SHM are designated in magenta and yellow, respectively. See also Figure S6.

(I) Close up of the HCDR1 and 2 domains of both Fabs and their interactions with the N156gp120A glycan and gp120 residues.

(J) Low pass filtered ApexGT5 + GT5-d42.16 cryo-EM map (gray) and (ApexGT2 + GT2-d42.16) – (ApexGT5 + GT5-d42.16) difference map (purple) showing the density associated with the N187 glycan and the slight shift in angle of approach of the GT2-d42.16 Fab (arrow).

(K) Close up of ApexGT5 loop2B on protomer C showing the cryo-EM map density (transparent gray) that bridges W188 at the tip of the loop and the N160gp120C glycan along with multiple loop models generated from multi-model refinement.

(L) All three gp120 protomers of ApexGT5 aligned to one another showing the conformation adopted by the protomer C loop.

CellPress
OPEN ACCESS

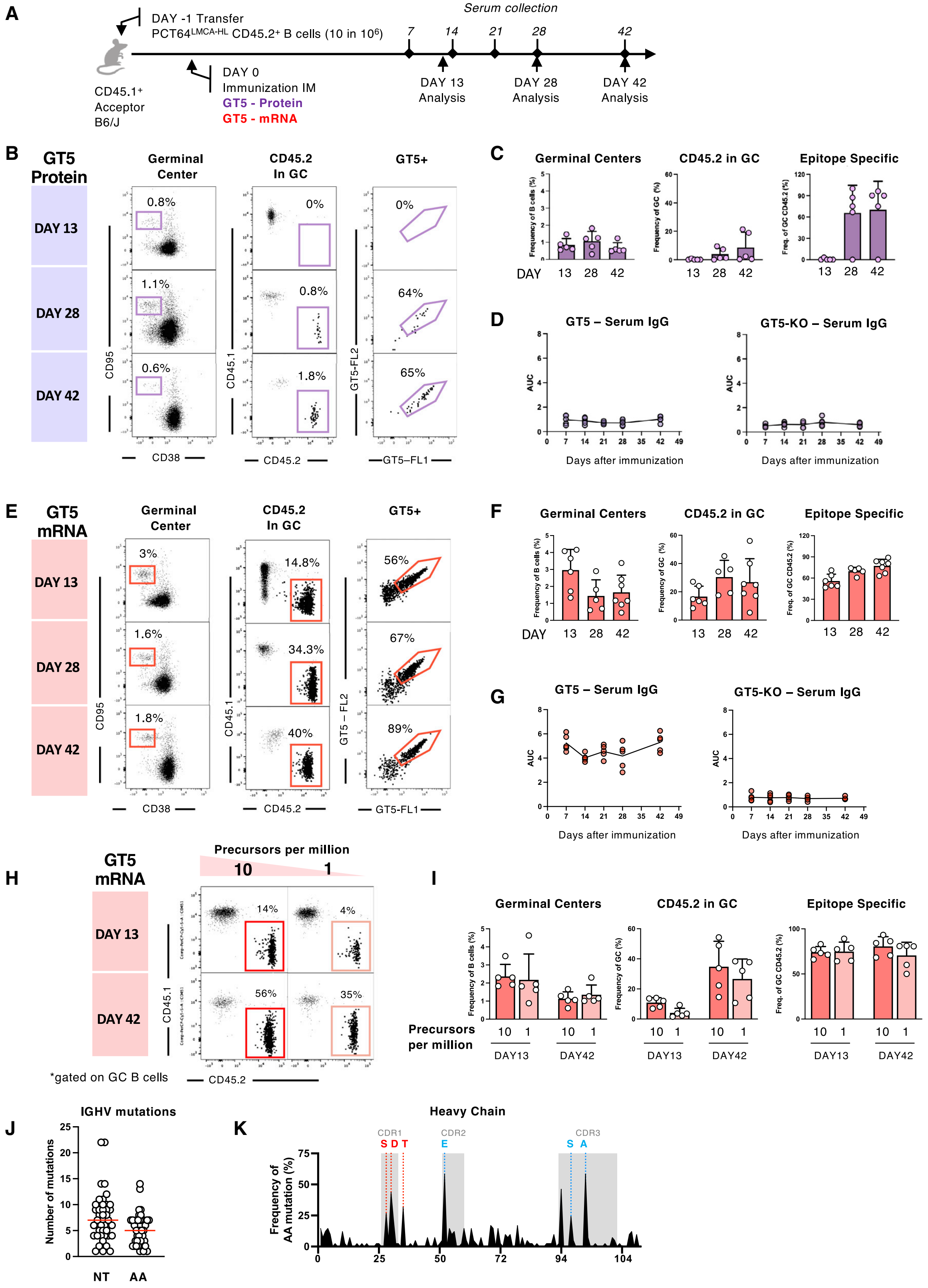

(legend on next page)

PCT64^{LMCA-L}, we obtained mice where PCT64$^{LMCA.JREV}$ IGH and IGK were paired in ~84% of the repertoire (Figure 7B).

Reverting the PCT64^{LMCA} J-gene mutations decreased the affinity of PCT64$^{LMCA.JREV}$ for GT2 (KD = 6.4 μM) and GT5 (KD = 347 nM). Antibodies with PCT64$^{LMCA.JREV}$ IGH paired with murine IGK had a geomean affinity of 48.1 nM among binders, lower than the 16.7 nM geomean affinity of binders for PCT64^{LMCA} IGH paired with the same murine IGK (Figure 7C). The lower affinity for PCT64$^{LMCA.JREV}$ was reflected in the low frequencies of naive B cells binding GT2 (5%) and GT5 (55%) probes (Figure 7D). To determine whether precursors in these affinity ranges could be triggered by immunization, PCT64$^{LMCA.JREV-H}$ B cells were transferred into CD45.1 WT mice (frequency: 100:10^{6}) prior to i.p.-immunization with either GT2 or GT5 protein trimers (Figure S7C). GCs developed in response to both immunogens, but no PCT64$^{LMCA.JREV-H}$ B cell activation was detected after GT2 immunization at 8 dpi. However, PCT64$^{LMCA.JREV}$ B cells were present in GCs after GT5 immunization (Figure S7D), demonstrating that the improved GT5 immunogen can activate PCT64 precursors with LMCA.JREV BCRs. At 28 dpi, mutation frequency analysis identified enriched sites distinct from those induced in PCT64^{LMCA} B cells by GT5 (Figure S7E). In particular, an aspartic acid (D) was acquired in position 117 in the HCDR3 in 98% of the GT5^{+} class-switched B cells (Figure S7E); this mutation is present in the original PCT64^{LMCA} sequence (Figure S7F), indicating a converging HCDR3-region maturation pathway.

The precursor frequency required for activation in PCT64$^{LMCA.JREV}$ was far from the estimated human range. Therefore, we tested whether GT5-mRNA trimers could elicit stronger responses. We transferred PCT64$^{LMCA.JREV}$ B cells (frequencies: 100, 20, and 10 per 10^{6}) and immunized recipient mice IM with 10 μg of mRNA-LNP encoding GT5 or 10 μg of GT5 soluble protein. At 13 dpi, GC responses with strong PCT64$^{LMCA.JREV-H}$ B cell recruitment were present in mRNA-immunized mice but no PCT64$^{LMCA.JREV-H}$ B cell activation was detected in protein-immunized mice (Figure S7G). We then compared responses to mRNA-GT5 IM and GT5 protein i.p. immunization 13 and 42 dpi in inguinal LNs or spleen (Figure 7E). At 13 dpi CD45.2 PCT64$^{LMCA.JREV}$ B cell responses to mRNA were 10–15 times higher than responses to protein (Figures 7F and 7G). Furthermore, strong CD45.2 responses were present in the low frequency groups (20 and 10 per 10^{6}) (Figures 7F and 7G). PCT64$^{LMCA.JREV}$ GC responses to mRNA were maintained and expanded (reaching ~20% of GC B cells) at 42 dpi in mice with a starting precursor frequency of 100 and 20 per 10^{6}. Responses faded in mice with lower precursor numbers and in mice immunized with protein GT5 (Figure 7H). Analysis of GT5^{+} class-switched B cells at 28 dpi revealed a mutation pattern overlapping with that induced by protein in JREV, characterized by an enrichment of Asp in position 117 in the HCDR3 (Figure S7H).

These results suggest that mRNA-encoded GT5 may lower the affinity threshold for precursor activation (Batista and Neuberger, 1998, 2000; Fleire et al., 2006). Thus, mRNA-LNP-encoded membrane-bound GT5 trimers may be promising candidates for the development of priming immunogens for PCT64-like precursors with diverse junctions and affinities.

DISCUSSION

Inducing bnAbs is a key objective in the quest for an HIV vaccine (Haynes and Burton, 2017). Central to these efforts is the structure-based design and validation of GT immunogens, which must activate rare precursor B cells, trigger durable GC responses, and induce desired mutations (Jardine et al., 2013). Here, we engineered two BCR KI mice that express two different precursors of the V2-apex bnAb PCT64 to evaluate GT immunogens (Landais et al., 2017). In our preclinical models, high-affinity ApexGT5 was superior to ApexGT2 at activating rare PCT64 precursors. Furthermore, nucleoside-modified mRNA immunogens, at the relative doses administered, improved activation over protein.

BCR-immunogen affinity is a major determinant of rare B cell activation (Abbott et al., 2018; Dosenovic et al., 2018), and V2-apex precursors are notably rare. Most HIV-1 bnAbs specific to V2-apex rely on HCDR3 loops with a 24–39 aa range to interact with their epitopes (Andrabi et al., 2015), and B cells bearing long HCDR3s (≥24 aa) represent only 3.5% of the human repertoire (Briney et al. 2012). However, PCT64 precursors, with 25 aa HCDR3s, are estimated to have an upper frequency limit of 20 per 10^{6} B cells. In contrast, 3.3 per 10^{6} of B cells are eOD-GT8-specific VRC01 precursors (Jardine et al., 2016;

Figure 6. GT5 mRNA effectively activates rare precursors

(A) Schematic of IM immunization. Mice received either GT5 trimers adjuvanted with SIGMA or GT5 mRNA. Experiments performed in duplicate with one presented; n = 5.

(B) Representative FACS plots of LN B cells at 13, 28, and 42 dpi IM with GT5 trimers, and SIGMA adjuvant showing GCs, CD45.2^{+} B cells in GC, and GT5-binding CD45.2 B cells.

(C) Quantification of GCs, CD45.2^{+} B cells in GC, and GT5-binding CD45.2 B cells at 13, 28, and 42 dpi IM with GT5 trimers and SIGMA adjuvant (n = 5). Data are represented as mean ± SD.

(D) ELISA quantification of GT5-binding (left) and GT5-KO-binding (right) serum IgG from PCT64^{LMCA} recipient mice immunized with GT5 trimers and SIGMA adjuvant (as in B and C). Line represents mean value.

(E) Representative plots of LN B cells at 13, 28, and 42 dpi IM with GT5 mRNA.

(F) Quantification of LN GCs, CD45.2^{+} B cells in GC, and GT5-binding CD45.2 B cells at 13, 28, and 42 dpi IM with GT5 mRNA (n = 5). Bars are mean ± SD.

(G) ELISA quantification of GT5-binding (left) and GT5-KO-binding (right) serum IgG from PCT64^{LMCA} recipient mice immunized with GT5 mRNA (as in E and F). Line represents mean value.

(H) Representative plots of LN B cells at 13 and 42 dpi IM with GT5 trimers in mice with a starting PCT64 precursor frequency of 10 and 1 per 10^{6} B cells. Plots show the frequency of CD45.2^{+} B cells in GC. Experiments were performed in duplicate with one presented; n = 5.

(I) Quantification of GCs, CD45.2^{+} B cells in GC, and GT5-binding CD45.2 B cells at 13 and 42 dpi IM with GT5 trimers in mice with a starting PCT64 precursor frequency of 10 or 1 per 10^{6} B cells. Bars are mean ± SD.

(J) Total nt and aa mutations acquired in PCT64^{LMCA} IGH and IGK V genes at 42 dpi with GT5 mRNA in 2 different mice. Red line indicates mean.

(K) Frequencies of IGH aa mutations per residue at 42 dpi. HCDRs boxed in gray. Red = present in mature PCT64; blue = present in early PCT64-lineage isolates.

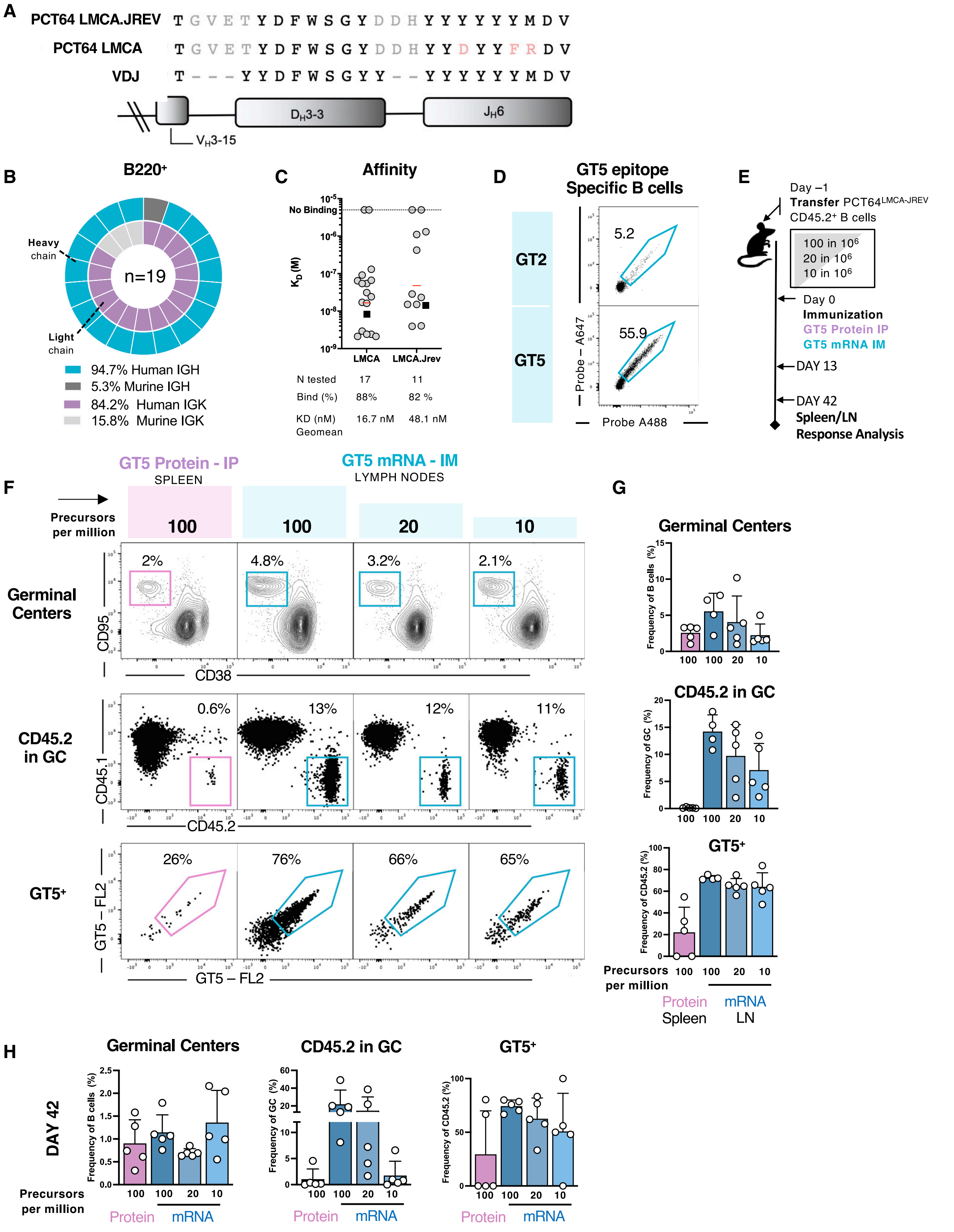

(legend on next page)

Havenar-Daughton et al., 2018; Lee et al., 2021). At the lowest precursor frequency investigated, the higher-affinity GT5 initiated strong, sustained activation with a 10-fold increase over GT2 in GC recruitment.

Both GT2 and GT5 elicited on-track SHM, induced reproducible HC mutation fingerprints, and did not display stringent LC restriction in our HC model, where various murine LCs pair with germline PCT64 IGHs. This suggests a predominantly IGH-dependent selection process and validates the applicability of trimer design targeting HCDR3-dominant bnAb precursors previously tested only for BG18 (Steichen et al., 2019). There was, however, a marginal role for LCs: certain murine IGK pairings were so detrimental as to completely abrogate immunogen binding. In contrast, some advantageous IGK pairings increased the affinity of the original germline antibody. The capacity of a single immunogen to engage multiple B cell clones independently from the paired LC expands the range of precursors that can be elicited through immunization, raising the odds of developing bnAbs.

We also found that immunization with mRNA-LNP coding for membrane-bound GT5 was superior to soluble GT5 protein at triggering low-frequency PCT64-precursor B cells. This may be because V2-apex bnAbs tightly bind to quaternary epitopes, recognizing the N-linked glycan at residue 160 (N160) and interacting with a protein surface of the V2 domain of gp120 encompassing multiple protomers; they do not bind well to monomeric GP120 (Andrabi et al., 2015, 2017; Gift et al., 2017; Moore et al., 2017). IM immunization may negatively impact the successful presentation of an intact, fully-formed trimer structure; a similar effect has been previously reported in mice (Hu et al., 2015). Nucleoside-modified mRNA-LNP allows antigens to be translated into protein directly into the host's cells, minimizing processing by protease in antigen-presenting cells and increasing the chances that B cells encounter a well-folded trimer. Additionally, the amount of trimer produced after mRNA immunization may be larger than the protein dose, and antigen availability may be increased as a consequence of protein expression kinetics (Pardi et al., 2015, 2018; Lederer et al., 2020). Indeed, similar responses are observed when immunogen administration is extended through an osmotic pump (Tam et al., 2016; Cirelli et al., 2019).

An HIV vaccine conferring broad protection will likely require the elicitation of several lines of antibodies to target multiple sites on Env. In this study, we validated HCDR3-focused GT immunogen design for the V2-apex epitope and demonstrated the utility of mRNA-LNP. As mRNA could simplify the delivery of multiple structurally sound immunogens, this work, alongside the array of GT vaccine candidates in various stages of clinical testing, may contribute to the development of a multi-component HIV vaccine.

Limitations of the study

Our interpretation of SHM emphasized residues in the mature PCT64 bnAb; for some of those mutations, the precise role in binding remains to be clarified. Furthermore, it is not trivial to identify off-track mutations that might instead hinder bnAb development, particularly when they may only be detrimental in the context of other mutations.

While the PCT64-like mutations acquired in this study conferred autologous virus neutralization, booster immunizations with more native-like immunogens would likely be needed to achieve cross-clade neutralization. Specifically, as R169 is rare among WT HIV isolates (<10%), it will be important to determine whether heterologous boost immunization can reduce the neutralization dependence on R169 observed. Finally, our conclusions have been derived from mouse models; further work will be required to see if they apply in non-human primates or humans.

STAR★METHODS

Detailed methods are provided in the online version of this paper and include the following:

- KEY RESOURCES TABLE
- RESOURCE AVAILABILITY
  - Lead contact
  - Data and code availability
- EXPERIMENTAL MODEL AND SUBJECT DETAILS
  - Mice
  - Generation of PCT64^{LMCA} knock-in (KI) mice
- METHOD DETAILS
  - Immunizations
  - Immunogen and FACS probe production
  - ELISA
  - Flow cytometry
  - BCR sequencing
  - 10x Genomics B cell repertoire analysis
  - Phylogenetic analysis
  - Surface plasmon resonance (SPR)
  - Neutralization assay
  - Cryo-EM sample preparation
  - Cryo-EM data collection

Figure 7. GT5-mRNA immunization activates a J-region-reverted PCT64 precursor

(A) Sequence alignment of the HCDR3 of PCT64$^{LMCA.JREV}$, PCT64^{LMCA} and germline VH3-15, DH3-3 and JH6.

(B) Human PCT64 LMCA.JREV HC (teal), murine HC (dark gray), human PCT64$^{LMCA.JREV}$ IGK (purple) and murine LC (light gray) sequences amplified from single cell sorted B220^{+} naive B cells from two PCT64$^{LMCA.JREV}$ mice. n = pairs amplified. See also Figures S7A and S7B.

(C) SPR affinity measurement against GT5 of LMCA and LMCA.JREV IGH paired with various murine IGK.

(D) FACS plots with epitope specific GT2-binding (top) and GT5 binding of peripheral B cells in naive PCT64$^{LMCA.JREV}$ mouse model. Events were pre-gated on lymphocytes/singlets/CD4^{-}CD8^{-}F4/80^{-}Gr1^{-}/B220^{+} B cells. See also Figures S7C–S7F.

(E) Schematic of study design. Recipient mice at 100, 20, or 10 PCT64$^{LMCA.JREV}$ per 10^6 B cells prior to immunization i.p. with GT5 trimers or IM with GT5 mRNA; responses analyzed 13 dpi. Experiments performed in triplicate with one presented; n = 4 or 5. See also Figures S7G and S7H.

(F) Representative FACS plots of GCs, CD45.2 PCT64$^{LMCA.JREV}$ present in GC, and GT5 specific responses at 13 dpi after immunization with GT5 protein i.p. (pink) or GT5 mRNA IM (teal). Spleen were analyzed for i.p. responses and inguinal LN for IM responses.

(G) Quantification of responses in GCs as in (E) and (F) (n = 4 or 5). Bars are mean ± SD.

(H) Quantification of GC responses, frequency of CD45.2 PCT64$^{LMCA.JREV}$ B cells in GC and GT5 specific responses at 42 dpi (n = 5). Bars are mean ± SD.

CellPress
OPEN ACCESS

- ○ Cryo-EM data processing
- ○ Model building and figure preparation
- QUANTIFICATION AND STATISTICAL ANALYSIS

SUPPLEMENTAL INFORMATION

Supplemental information can be found online at https://doi.org/10.1016/j.immuni.2022.09.003.

ACKNOWLEDGMENTS

This work was supported by the IAVI Neutralizing Antibody Center through the Collaboration for AIDS Vaccine Discovery (CAVD) grants OPP1084519, OPP1147787, INV-034657 (to W.R.S.), OPP1196345/INV-008813 (to A.B.W. and W.R.S.), OPP1115782/INV-002916 (to A.B.W.) and INV009585 (to F.D.B.). This work was also funded by the Bill and Melinda Gates Foundation and CAVD, the National Institute of Allergy and Infectious Diseases (NIAID) UM1 AI100663 (Scripps Center for HIV/AIDS Vaccine Immunology and Immunogen Discovery) (to F.D.B. and W.R.S.), and UM1 AI144462 (Scripps Consortium for HIV/AIDS Vaccine Development) (to W.R.S., A.B.W., and F.D.B.); by the Ragon Institute of MGH, MIT, and Harvard (to W.R.S. and F.D.B.); and by EMBO fellowship ALTF 84-2018 (to E.M.). Graphical abstract by Christina Corbaci.

AUTHOR CONTRIBUTIONS

E.M. planned, designed, and performed *in vivo* experiments, serology, FACS, single-cell sorting, and BCR sequencing. J.R.W. and K.M.M. provided immunogens, probes, elicited antibodies, and antibody sequences for BCR knockins and helped plan experiments. Y.C.L. performed adoptive transfer. S.K. performed B cell sorting and animal experiments. Z.T.B. conducted structural studies. E.A.L. and A.L. made pseudoviruses and performed neutralization assays. O.K. and A.K. carried out SPR analyses. U.N. and J.W. performed 10x genomic sequencing. U.N. and Y.C.L. generated murine models. J.M.S. helped plan experiments and contributed immunogens and data interpretation. S.P. helped with murine model characterization. M.P. supported laboratory work. K.K. supervised mouse colony management. S.R.W. contributed to manuscript writing. S.F., S.H., and A.C. provided mRNA-LNPs. D.S. supervised neutralization assays. A.B.W. supervised the structural studies. E.M., J.R.W., K.M.M., Z.T.B., J.M.S., A.B.W., W.R.S., and F.D.B. interpreted data. W.R.S. and F.D.B. conceived and supervised the studies. E.M., Z.T.B., and F.D.B. wrote the paper; J.R.W., K.M.M., J.M.S., S.R.W., A.B.W., S.H., A.C., and W.R.S. edited the paper.

DECLARATION OF INTERESTS

J.R.W., K.M.M., J.M.S., and W.R.S. are named inventors on patent applications filed by Scripps and IAVI regarding ApexGT immunogens in this manuscript.

Received: January 13, 2022
Revised: May 31, 2022
Accepted: September 2, 2022
Published: September 29, 2022

REFERENCES

Abbott, R.K., Lee, J.H., Menis, S., Skog, P., Rossi, M., Ota, T., Kulp, D.W., Bhullar, D., Kalyuzhniy, O., Havenar-Daughton, C., et al. (2018). Precursor frequency and affinity determine B cell competitive fitness in germinal centers, tested with germline-targeting HIV vaccine immunogens. Immunity *48*, 133–146. https://doi.org/10.1016/j.immuni.2017.11.023.

Agirre, J., Iglesias-Fernández, J., Rovira, C., Davies, G.J., Wilson, K.S., and Cowtan, K.D. (2015). Privateer: software for the conformational validation of carbohydrate structures. Nat. Struct. Mol. Biol. *22*, 833–834. https://doi.org/10.1038/nsmb.3115.

Andrabi, R., Voss, J.E., Liang, C.H., Briney, B., McCoy, L.E., Wu, C.Y., Wong, C.H., Poignard, P., and Burton, D.R. (2015). Identification of common features in prototype broadly neutralizing antibodies to HIV envelope V2 apex to facilitate vaccine design. Immunity *43*, 959–973. https://doi.org/10.1016/j.immuni.2015.10.014.

Andrabi, R., Su, C.Y., Liang, C.H., Shivatare, S.S., Briney, B., Voss, J.E., Nawazi, S.K., Wu, C.Y., Wong, C.H., and Burton, D.R. (2017). Glycans function as anchors for antibodies and help drive HIV broadly neutralizing antibody development. Immunity *47*, 524–537.e3. https://doi.org/10.1016/j.immuni.2017.08.006.

Baden, L.R., El Sahly, H.M., Essink, B., Kotloff, K., Frey, S., Novak, R., Diemert, D., Spector, S.A., Rouphael, N., Creech, C.B., et al. (2021). Efficacy and safety of the mRNA-1273 SARS-CoV-2 vaccine. N. Engl. J. Med. Overseas. Ed. *384*, 403–416. https://doi.org/10.1056/NEJMoa2035389.

Barad, B.A., Echols, N., Wang, R.Y.R., Cheng, Y., DiMaio, F., Adams, P.D., and Fraser, J.S. (2015). EMRinger: Side chain-directed model and map validation for 3D cryo-electron microscopy. Nat. Methods *12*, 943–946. https://doi.org/10.1038/nmeth.3541.

Batista, F.D., and Neuberger, M.S. (1998). Affinity dependence of the B cell response to antigen: a threshold, a ceiling, and the importance of off-rate. Immunity *8*, 751–759. https://doi.org/10.1016/S1074-7613(00)80580-4.

Batista, F.D., and Neuberger, M.S. (2000). B cells extract and present immobilized antigen: implications for affinity discrimination. EMBO J. *19*, 513–520. https://doi.org/10.1093/emboj/19.4.513.

von Boehmer, L., Liu, C., Ackerman, S., Gitlin, A.D., Wang, Q., Gazumyan, A., and Nussenzweig, M.C. (2016). Sequencing and cloning of antigen-specific antibodies from mouse memory B cells. Nat. Protoc. *11*, 1908–1923. https://doi.org/10.1038/nprot.2016.102.

Briney, B., Sok, D., Jardine, J.G., Kulp, D.W., Skog, P., Menis, S., Jacak, R., Kalyuzhniy, O., de Val, N., Sesterhenn, F., et al. (2016). Tailored immunogens direct affinity maturation toward HIV neutralizing antibodies. Cell *166*, 1459–1470.e11. https://doi.org/10.1016/j.cell.2016.08.005.

Briney, B., Inderbitzin, A., Joyce, C., and Burton, D.R. (2019). Commonality despite exceptional diversity in the baseline human antibody repertoire. Nature, 1. https://doi.org/10.1038/s41586-019-0879-y.

Briney, B.S., Willis, J.R., and Crowe, J.E. (2012). Human peripheral blood antibodies with long HCDR3s are established primarily at original recombination using a limited subset of germline genes. PLoS One *7*, e36750. https://doi.org/10.1371/journal.pone.0036750.

Brochet, X., Lefranc, M.P., and Giudicelli, V. (2008). IMGT/V-QUEST: the highly customized and integrated system for IG and TR standardized V-J and V-D-J sequence analysis. Nucleic Acids Res. *36*, 503–508. (Web Server issue). https://doi.org/10.1093/nar/gkn316.

Burton, D.R., and Hangartner, L. (2016). Broadly neutralizing antibodies to HIV and their role in vaccine design. Annu. Rev. Immunol. *34*, 635–659. https://doi.org/10.1146/annurev-immunol-041015-055515.

Chen, V.B., Arendall, W.B., 3rd, Headd, J.J., Keedy, D.A., Immormino, R.M., Kapral, G.J., Murray, L.W., Richardson, J.S., and Richardson, D.C. (2010). MolProbity: All-atom structure validation for macromolecular crystallography. Acta Crystallogr. D Biol. Crystallogr. *66*, 12–21. https://doi.org/10.1107/S0907444909042073.

Chen, X., Zhou, T., Schmidt, S.D., Duan, H., Cheng, C., Chuang, G.Y., Gu, Y., Louder, M.K., Lin, B.C., Shen, C.H., et al. (2021). Vaccination induces maturation in a mouse model of diverse unmutated VRC01-class precursors to HIV-neutralizing antibodies with >50% breadth. Immunity *54*, 324–339.e8. https://doi.org/10.1016/j.immuni.2020.12.014.

Cirelli, K.M., Carnathan, D.G., Nogal, B., Martin, J.T., Rodriguez, O.L., Upadhyay, A.A., Enemuo, C.A., Gebru, E.H., Choe, Y., Viviano, F., et al. (2019). Slow delivery immunization enhances HIV neutralizing antibody and germinal center responses via modulation of immunodominance. Cell *177*, 1153–1171.e28. https://doi.org/10.1016/j.cell.2019.04.012.

Doria-Rose, N.A., Schramm, C.A., Gorman, J., Moore, P.L., Bhiman, J.N., DeKosky, B.J., Ernandes, M.J., Georgiev, I.S., Kim, H.J., Pancera, M., et al. (2014). Developmental pathway for potent V1V2-directed HIV-neutralizing antibodies. Nature *509*, 55–62. https://doi.org/10.1038/nature13036.

Doria-Rose, N.A., and Landais, E. (2019). Coevolution of HIV-1 and broadly neutralizing antibodies. Curr. Opin. HIV AIDS *14*, 286–293. https://doi.org/10.1097/COH.0000000000000550.

Dosenovic, P., von Boehmer, L., Escolano, A., Jardine, J., Freund, N.T., Gitlin, A.D., McGuire, A.T., Kulp, D.W., Oliveira, T., Scharf, L., et al. (2015). Immunization for HIV-1 broadly neutralizing antibodies in human Ig knockin mice. Cell *161*, 1505–1515. https://doi.org/10.1016/j.cell.2015.06.003.

Dosenovic, P., Kara, E.E., Pettersson, A.K., McGuire, A.T., Gray, M., Hartweger, H., Thientosapol, E.S., Stamatatos, L., and Nussenzweig, M.C. (2018). Anti–HIV-1 B cell responses are dependent on B cell precursor frequency and antigen-binding affinity. Proc. Natl. Acad. Sci. USA *115*, 4743–4748. https://doi.org/10.1073/pnas.1803457115.

Edgar, R.C. (2004). MUSCLE: multiple sequence alignment with high accuracy and high throughput. Nucleic Acids Res. *32*, 1792–1797. https://doi.org/10.1093/nar/gkh340.

Emsley, P., and Crispin, M. (2018). Structural analysis of glycoproteins: building N-linked glycans with coot. Acta Crystallogr. D Struct. Biol. *74*, 256–263. https://doi.org/10.1107/S2059798318005119.

Escolano, A., Steichen, J.M., Dosenovic, P., Kulp, D.W., Golijanin, J., Sok, D., Freund, N.T., Gitlin, A.D., Oliveira, T., Araki, T., et al. (2016). Sequential immunization elicits broadly neutralizing anti-HIV-1 antibodies in Ig knockin mice. Cell *166*, 1445–1458.e12. https://doi.org/10.1016/j.cell.2016.07.030.

Fleire, S.J., Goldman, J.P., Carrasco, Y.R., Weber, M., Bray, D., and Batista, F.D. (2006). B cell ligand discrimination through a spreading and contraction response. Science *312*, 738–741. https://doi.org/10.1126/science.1123940.

Flemming, A. (2018). Needles in a haystack: the quest for bnAbs. Nature Research *861*, 2018.

Georgiev, I.S., Doria-Rose, N.A., Zhou, T., Kwon, Y.D., Staupe, R.P., Moquin, S., Chuang, G.Y., Louder, M.K., Schmidt, S.D., Altae-Tran, H.R., et al. (2013). Delineating antibody recognition in polyclonal sera from patterns of HIV-1 isolate neutralization. Science *340*, 751–756. https://doi.org/10.1126/science.1233989.

Gift, S.K., Leaman, D.P., Zhang, L., Kim, A.S., and Zwick, M.B. (2017). Functional stability of HIV-1 envelope trimer affects accessibility to broadly neutralizing antibodies at its apex. J. Virol. *91*, 7–10. https://doi.org/10.1128/jvi.01216-17.

Giudicelli, V., Brochet, X., and Lefranc, M.-P. (2011). IMGT/V-QUEST: IMGT standardized analysis of the immunoglobulin (IG) and T cell receptor (TR) nucleotide sequences. Cold Spring Harb. Protoc. *2011*, 695–715. https://doi.org/10.1101/pdb.prot5633.

Gorman, J., Soto, C., Yang, M.M., Davenport, T.M., Guttman, M., Bailer, R.T., Chambers, M., Chuang, G.Y., DeKosky, B.J., Doria-Rose, N.A., et al. (2016). Structures of HIV-1 Env V1V2 with broadly neutralizing antibodies reveal commonalities that enable vaccine design. Nat. Struct. Mol. Biol. *23*, 81–90. https://doi.org/10.1038/nsmb.3144.

Hardy, R.R., Carmack, C.E., Shinton, S.A., Kemp, J.D., and Hayakawa, K. (1991). Resolution and characterization of pro-B and pre-pro-B cell stages in normal mouse bone marrow. J. Exp. Med. *173*, 1213–1225. https://doi.org/10.1084/jem.173.5.1213.

Havenar-Daughton, C., Sarkar, A., Kulp, D.W., Toy, L., Hu, X., Deresa, I., Kalyuzhniy, O., Kaushik, K., Upadhyay, A.A., Menis, S., et al. (2018). The human naive B cell repertoire contains distinct subclasses for a germline-targeting HIV-1 vaccine immunogen. Sci. Transl. Med. *10*, 1–16. https://doi.org/10.1126/scitranslmed.aat0381.

Haynes, B.F., Shaw, G.M., Korber, B., Kelsoe, G., Sodroski, J., Hahn, B.H., Borrow, P., and McMichael, A.J. (2016). HIV-host interactions: implications for vaccine design. Cell Host Microbe *19*, 292–303. https://doi.org/10.1016/j.chom.2016.02.002.

Haynes, B.F., and Burton, D.R. (2017). Developing an HIV vaccine. Science *355*, 1129–1130. https://doi.org/10.1126/science.aan0662.

Hoot, S., McGuire, A.T., Cohen, K.W., Strong, R.K., Hangartner, L., Klein, F., Diskin, R., Scheid, J.F., Sather, D.N., Burton, D.R., and Stamatatos, L. (2013). Recombinant HIV envelope proteins fail to engage germline versions of anti-CD4bs bNAbs. PLoS Pathog. *9*, e1003106. https://doi.org/10.1371/journal.ppat.1003106.

Hu, J.K., Crampton, J.C., Cupo, A., Ketas, T., van Gils, M.J., Sliepen, K., de Taeye, S.W., Sok, D., Ozorowski, G., Deresa, I., et al. (2015). Murine antibody responses to cleaved soluble HIV-1 envelope trimers are highly restricted in specificity. J. Virol. *89*, 10383–10398. https://doi.org/10.1128/jvi.01653-15.

Jardine, J., Julien, J.P., Menis, S., Ota, T., Kalyuzhniy, O., McGuire, A., Sok, D., Huang, P.S., MacPherson, S., Jones, M., et al. (2013). Rational HIV immunogen design to target specific germline B Cell receptors. Science *340*, 711–716. https://doi.org/10.1126/science.1234150.

Jardine, J.G., Ota, T., Sok, D., Pauthner, M., Kulp, D.W., Kalyuzhniy, O., Skog, P.D., Thinnes, T.C., Bhullar, D., Briney, B., et al. (2015). Priming a broadly neutralizing antibody response to HIV-1 using a germline-targeting immunogen. Science *349*, 156–161. https://doi.org/10.1126/science.aac5894.

Jardine, J.G., Sok, D., Julien, J.P., Briney, B., Sarkar, A., Liang, C.H., Scherer, E.A., Henry Dunand, C.J., Adachi, Y., Diwanji, D., and Hsueh, J. (2016). Minimally mutated HIV-1 broadly neutralizing antibodies to guide reductionist vaccine design. PLoS Pathog. 1–33. https://doi.org/10.1371/journal.ppat.1005815.

Jones, D.T., Taylor, W.R., and Thornton, J.M. (1992). The rapid generation of mutation data matrices from protein sequences. Comput. Appl. Biosci. *8*, 275–282. https://doi.org/10.1093/bioinformatics/8.3.275.

Julien, J.P., Lee, J.H., Cupo, A., Murin, C.D., Derking, R., Hoffenberg, S., Caulfield, M.J., King, C.R., Marozsan, A.J., Klasse, P.J., et al. (2013). Asymmetric recognition of the HIV-1 trimer by broadly neutralizing antibody PG9. Proc. Natl. Acad. Sci. USA *110*, 4351–4356. https://doi.org/10.1073/pnas.1217537110.

Kelsoe, G., and Haynes, B.F. (2017). Host controls of HIV broadly neutralizing antibody development. Immunol. Rev. *275*, 79–88. https://doi.org/10.1111/imr.12508.

Kimanius, D., Forsberg, B.O., Scheres, S.H., and Lindahl, E. (2016). Accelerated cryo-EM structure determination with parallelisation using GPUS in RELION-2. Elife *5*, 1–21. https://doi.org/10.7554/eLife.18722.

Klein, F., Mouquet, H., Dosenovic, P., Scheid, J.F., Scharf, L., and Nussenzweig, M.C. (2013). Antibodies in HIV-1 vaccine development and therapy. Science *341*, 1199–1204. https://doi.org/10.1126/science.1241144.

Landais, E., Huang, X., Havenar-Daughton, C., Murrell, B., Price, M.A., Wickramasinghe, L., Ramos, A., Bian, C.B., Simek, M., Allen, S., et al. (2016). Broadly neutralizing antibody responses in a large longitudinal sub-saharan HIV primary infection cohort. PLoS Pathog. *12*, 1–22. https://doi.org/10.1371/journal.ppat.1005369.

Landais, E., Murrell, B., Briney, B., Murrell, S., Rantalainen, K., Berndsen, Z.T., Ramos, A., Wickramasinghe, L., Smith, M.L., Eren, K., et al. (2017). HIV envelope glycoform heterogeneity and localized diversity govern the initiation and maturation of a V2 apex broadly neutralizing antibody lineage. Immunity *47*, 990–1003.e9. https://doi.org/10.1016/j.immuni.2017.11.002.

Lederer, K., Castaño, D., Gómez Atria, D., Oguin, T.H., 3rd, Wang, S., Manzoni, T.B., Muramatsu, H., Hogan, M.J., Amanat, F., Cherubin, P., et al. (2020). SARS-CoV-2 mRNA vaccines foster potent antigen-specific germinal center responses associated with neutralizing antibody generation. Immunity *53*, 1281–1295.e5. https://doi.org/10.1016/j.immuni.2020.11.009.

Lee, J.H., Toy, L., Kos, J.T., Safonova, Y., Schief, W.R., Havenar-Daughton, C., Watson, C.T., and Crotty, S. (2021). Vaccine genetics of IGHV1-2 VRC01-class broadly neutralizing antibody precursor naïve human B cells. npj Vaccines *6*, 1–12. https://doi.org/10.1038/s41541-021-00376-7.

Liao, H.X., Bonsignori, M., Alam, S.M., McLellan, J.S., Tomaras, G.D., Moody, M.A., Kozink, D.M., Hwang, K.K., Chen, X., Tsao, C.Y., et al. (2013). Vaccine induction of antibodies against a structurally heterogeneous site of immune pressure within HIV-1 envelope protein variable regions 1 and 2. Immunity *38*, 176–186. https://doi.org/10.1016/j.immuni.2012.11.011.

Lin, Y.C., Pecetta, S., Steichen, J.M., Kratochvil, S., Melzi, E., Arnold, J., Dougan, S.K., Wu, L., Kirsch, K.H., Nair, U., et al. (2018). One-step CRISPR/Cas9 method for the rapid generation of human antibody heavy chain knock-in mice. EMBO J. *37*, e99243. https://doi.org/10.15252/embj.201899243.

CellPress
OPEN ACCESS

McGuire, A.T., Hoot, S., Dreyer, A.M., Lippy, A., Stuart, A., Cohen, K.W., Jardine, J., Menis, S., Scheid, J.F., West, A.P., et al. (2013). Engineering HIV envelope protein to activate germline B cell receptors of broadly neutralizing anti-CD4 binding site antibodies. J. Exp. Med. *210*, 655–663. https://doi.org/10.1084/jem.20122824.

McLellan, J.S., Pancera, M., Carrico, C., Gorman, J., Julien, J.P., Khayat, R., Louder, R., Pejchal, R., Sastry, M., Dai, K., et al. (2011). Structure of HIV-1 gp120 V1/V2 domain with broadly neutralizing antibody PG9. Nature *480*, 336–343. https://doi.org/10.1038/nature10696.

Moore, P.L., Gorman, J., Doria-Rose, N.A., and Morris, L. (2017). Ontogeny-based immunogens for the induction of V2-directed HIV broadly neutralizing antibodies. Immunol. Rev. *275*, 217–229. https://doi.org/10.1111/imr.12501.

Mouquet, H., Scharf, L., Euler, Z., Liu, Y., Eden, C., Scheid, J.F., Halper-Stromberg, A., Gnanapragasam, P.N.P., Spencer, D.I.R., Seaman, M.S., et al. (2012). Complex-type N-glycan recognition by potent broadly neutralizing HIV antibodies. Proc. Natl. Acad. Sci. USA *109*, E3268–E3277. https://doi.org/10.1073/pnas.1217207109.

Ng'uni, T., Chasara, C., and Ndhlovu, Z.M. (2020). Major Scientific Hurdles in HIV vaccine development: historical perspective and future directions. Front. Immunol. *11*, 590780–590817. https://doi.org/10.3389/fimmu.2020.590780.

Ota, T., Doyle-Cooper, C., Cooper, A.B., Doores, K.J., Aoki-Ota, M., Le, K., Schief, W.R., Wyatt, R.T., Burton, D.R., and Nemazee, D. (2013). B Cells from knock-in mice expressing broadly neutralizing HIV antibody b12 carry an Innocuous B cell receptor responsive to HIV vaccine candidates. J. Immunol. *191*, 3179–3185. https://doi.org/10.4049/jimmunol.1301283.

Pancera, M., McLellan, J.S., Wu, X., Zhu, J., Changela, A., Schmidt, S.D., Yang, Y., Zhou, T., Phogat, S., Mascola, J.R., and Kwong, P.D. (2010). Crystal structure of PG16 and chimeric dissection with somatically related PG9: structure-function analysis of two quaternary-specific antibodies that effectively neutralize HIV-1. J. Virol. *84*, 8098–8110. https://doi.org/10.1128/jvi.00966-10.

Pancera, M., Shahzad-Ul-Hussan, S., Doria-Rose, N.A., McLellan, J.S., Bailer, R.T., Dai, K., Loesgen, S., Louder, M.K., Staupe, R.P., Yang, Y., et al. (2013). Structural basis for diverse N-glycan recognition by HIV-1-neutralizing V1-V2-directed antibody PG16. Nat. Struct. Mol. Biol. *20*, 804–813. https://doi.org/10.1038/nsmb.2600.

Pardi, N., Tuyishime, S., Muramatsu, H., Kariko, K., Mui, B.L., Tam, Y.K., Madden, T.D., Hope, M.J., and Weissman, D. (2015). Expression kinetics of nucleoside-modified mRNA delivered in lipid nanoparticles to mice by various routes. J. Contr. Release *217*, 345–351. https://doi.org/10.1016/j.jconrel.2015.08.007.

Pardi, N., Hogan, M.J., Porter, F.W., and Weissman, D. (2018). mRNA vaccines-a new era in vaccinology. Nat. Rev. Drug Discov. *17*, 261–279. https://doi.org/10.1038/nrd.2017.243.

Pejchal, R., Walker, L.M., Stanfield, R.L., Phogat, S.K., Koff, W.C., Poignard, P., Burton, D.R., and Wilson, I.A. (2010). Structure and function of broadly reactive antibody PG16 reveal an H3 subdomain that mediates potent neutralization of HIV-1. Proc. Natl. Acad. Sci. USA *107*, 11483–11488. https://doi.org/10.1073/pnas.1004600107.

Pettersen, E.F., Goddard, T.D., Huang, C.C., Couch, G.S., Greenblatt, D.M., Meng, E.C., and Ferrin, T.E. (2004). UCSF Chimera - A visualization system for exploratory research and analysis. J. Comput. Chem. *25*, 1605–1612. https://doi.org/10.1002/jcc.20084.

Pettersen, E.F., Goddard, T.D., Huang, C.C., Meng, E.C., Couch, G.S., Croll, T.I., Morris, J.H., and Ferrin, T.E. (2021). UCSF chimeraX: structure visualization for researchers, educators, and developers. Protein Sci. *30*, 70–82. https://doi.org/10.1002/pro.3943.

Pintilie, G.D., Zhang, J., Goddard, T.D., Chiu, W., and Gossard, D.C. (2010). Quantitative analysis of cryo-EM density map segmentation by watershed and scale-space filtering, and fitting of structures by alignment to regions. J. Struct. Biol. *170*, 427–438. https://doi.org/10.1016/j.jsb.2010.03.007.

Price, M.N., Dehal, P.S., and Arkin, A.P. (2010). FastTree 2 – Approximately maximum-likelihood trees for large alignments. A.F.Y. Poon, ed. *5*, e9490. https://doi.org/10.1371/journal.pone.0009490.

Punjani, A., Rubinstein, J.L., Fleet, D.J., and Brubaker, M.A. (2017). CryoSPARC: Algorithms for rapid unsupervised cryo-EM structure determination. Nat. Methods *14*, 290–296. https://doi.org/10.1038/nmeth.4169.

Rantalainen, K., Berndsen, Z.T., Murrell, S., Cao, L., Omorodion, O., Torres, J.L., Wu, M., Umotoy, J., Copps, J., Poignard, P., et al. (2018). Co-evolution of HIV envelope and apex-targeting neutralizing antibody lineage provides benchmarks for vaccine design. Cell Rep. *23*, 3249–3261. https://doi.org/10.1016/j.celrep.2018.05.046.

Rappuoli, R., Bottomley, M.J., D'Oro, U., Finco, O., and De Gregorio, E. (2016). Reverse vaccinology 2.0: human immunology instructs vaccine antigen design. J. Exp. Med. *213*, 469–481. https://doi.org/10.1084/JEM.20151960.

Sanders, R.W., and Moore, J.P. (2017). Native-like Env trimers as a platform for HIV-1 vaccine design. Immunol. Rev. *275*, 161–182. https://doi.org/10.1111/imr.12481.

Sok, D., Laserson, U., Laserson, J., Liu, Y., Vigneault, F., Julien, J.P., Briney, B., Ramos, A., Saye, K.F., Le, K., et al. (2013). The Effects of somatic hypermutation on neutralization and binding in the PGT121 family of broadly neutralizing HIV antibodies. PLoS Pathog. *9*, e1003754. https://doi.org/10.1371/journal.ppat.1003754.

Sok, D., Briney, B., Jardine, J.G., Kulp, D.W., Menis, S., Pauthner, M., Wood, A., Lee, E.C., Le, K.M., Jones, M., et al. (2016). Priming HIV-1 broadly neutralizing antibody precursors in human Ig loci transgenic mice. Science *353*, 1557–1560. https://doi.org/10.1126/science.aah3945.

Sok, D., and Burton, D.R. (2018). Recent progress in broadly neutralizing antibodies to HIV. Nat. Immunol. *19*, 1179–1188. https://doi.org/10.1038/s41590-018-0235-7.

Soto, C., Bombardi, R.G., Branchizio, A., Kose, N., Matta, P., Sevy, A.M., Sinkovits, R.S., Gilchuk, P., Finn, J.A., and Crowe, J.E., Jr. (2019). High frequency of shared clonotypes in human B cell receptor repertoires. Nature *566*, 398–402. https://doi.org/10.1038/s41586-019-0934-8.

Stamatatos, L., Pancera, M., and McGuire, A.T. (2017). Germline-targeting immunogens. Immunol. Rev. *275*, 203–216. https://doi.org/10.1111/imr.12483.

Steichen, J.M., Kulp, D.W., Tokatlian, T., Escolano, A., Dosenovic, P., Stanfield, R.L., McCoy, L.E., Ozorowski, G., Hu, X., Kalyuzhniy, O., et al. (2016). HIV vaccine design to target germline precursors of glycan-dependent broadly neutralizing antibodies. Immunity *45*, 483–496. https://doi.org/10.1016/j.immuni.2016.08.016.

Steichen, J.M., Lin, Y.C., Havenar-Daughton, C., Pecetta, S., Ozorowski, G., Willis, J.R., Toy, L., Sok, D., Liguori, A., Kratochvil, S., et al. (2019). A generalized HIV vaccine design strategy for priming of broadly neutralizing antibody responses. Science *366*, eaax4380. https://doi.org/10.1126/science.aax4380.

Suloway, C., Pulokas, J., Fellmann, D., Cheng, A., Guerra, F., Quispe, J., Stagg, S., Potter, C.S., and Carragher, B. (2005). Automated molecular microscopy: The new Leginon system. J. Struct. Biol. *151*, 41–60. https://doi.org/10.1016/j.jsb.2005.03.010.

Tam, H.H., Melo, M.B., Kang, M., Pelet, J.M., Ruda, V.M., Foley, M.H., Hu, J.K., Kumari, S., Crampton, J., Baldeon, A.D., et al. (2016). Sustained antigen availability during germinal center initiation enhances antibody responses to vaccination. Proc. Natl. Acad. Sci. USA *113*, E6639–E6648. https://doi.org/10.1073/pnas.1606050113.

Thomas, S.J., Moreira, E.D., Jr., Kitchin, N., Absalon, J., Gurtman, A., Lockhart, S., Perez, J.L., Pérez Marc, G., Polack, F.P., Zerbini, C., et al. (2021). Safety and Efficacy of the BNT162b2 mRNA Covid-19 vaccine through 6 months. N. Engl. J. Med. *385*, 1761–1773. https://doi.org/10.1056/nejmoa2110345.

Tian, M., Cheng, C., Chen, X., Duan, H., Cheng, H.L., Dao, M., Sheng, Z., Kimble, M., Wang, L., Lin, S., et al. (2016). Induction of HIV neutralizing antibody lineages in mice with diverse precursor repertoires. Cell *166*, 1471–1484.e18. https://doi.org/10.1016/j.cell.2016.07.029.

Walker, L.M., Phogat, S.K., Chan-Hui, P.Y., Wagner, D., Phung, P., Goss, J.L., Wrin, T., Simek, M.D., Fling, S., Mitcham, J.L., et al. (2009). Broad and potent neutralizing antibodies from an African donor reveal a new HIV-1 vaccine target. Science *326*, 285–289. https://doi.org/10.1126/science.1178746.

Walker, L.M., Simek, M.D., Priddy, F., Gach, J.S., Wagner, D., Zwick, M.B., Phogat, S.K., Poignard, P., and Burton, D.R. (2010). A limited number of antibody specificities mediate broad and potent serum neutralization in selected HIV-1 infected individuals. PLoS Pathog. *6*, 11–12. https://doi.org/10.1371/journal.ppat.1001028.

Wang, R.Y.R., Song, Y., Barad, B.A., Cheng, Y., Fraser, J.S., and DiMaio, F. (2016). Automated structure refinement of macromolecular assemblies from cryo-EM maps using Rosetta. Elife *5*. e17219–22. https://doi.org/10.7554/eLife.17219.

Wang, X., Ray, R., Kratochvil, S., Melzi, E., Lin, Y.C., Giguere, S., Xu, L., Warner, J., Cheon, D., Liguori, A., et al. (2021). Multiplexed CRISPR/CAS9-mediated engineering of pre-clinical mouse models bearing native human B cell receptors. EMBO J. *40*, 1–20. https://doi.org/10.15252/embj.2020105926.

Xiao, X., Chen, W., Feng, Y., Zhu, Z., Prabakaran, P., Wang, Y., Zhang, M.Y., Longo, N.S., and Dimitrov, D.S. (2009). Germline-like predecessors of broadly neutralizing antibodies lack measurable binding to HIV-1 envelope glycoproteins: Implications for evasion of immune responses and design of vaccine immunogens. Biochem. Biophys. Res. Commun. *390*, 404–409. https://doi.org/10.1016/j.bbrc.2009.09.029.

Zhang, K. (2016). Gctf: Real-time CTF determination and correction. J. Struct. Biol. *193*, 1–12. https://doi.org/10.1016/j.jsb.2015.11.003.

Zhang, L., Wang, W., and Wang, S. (2015). Effect of vaccine administration modality on immunogenicity and efficacy. Expert Rev. Vaccines *14*, 1509–1523. https://doi.org/10.1586/14760584.2015.1081067.

Zheng, S.Q., Palovcak, E., Armache, J.-P., Verba, K.A., Cheng, Y., and Agard, D.A. (2017). MotionCor2: Anisotropic correction of beam-induced motion for improved cryo-electron microscopy. Nat. Methods *14*, 331–332. https://doi.org/10.1038/nmeth.4193.

Zhou, T., Georgiev, I., Wu, X., Yang, Z.Y., Dai, K., Finzi, A., Kwon, Y.D., Scheid, J.F., Shi, W., Xu, L., et al. (2010). Structural basis for broad and potent neutralization of HIV-1 by antibody VRC01. Science *329*, 811–817. https://doi.org/10.1126/science.1192819.

Zivanov, J., Nakane, T., Forsberg, B.O., Kimanius, D., Hagen, W.J., Lindahl, E., and Scheres, S.H. (2018). New tools for automated high-resolution cryo-EM structure determination in RELION-3. Elife *7*, 1–22. https://doi.org/10.7554/eLife.42166.

Willis, J.R., Berndsen, Z.T., Ma, K.M., Steichen, J.M., Schiffner, T., Landais, E., Liguori, A., Kalyuzhniy, O., Allen, J.D., Baboo, A., et al., (2022) Human immunoglobulin repertoire-guided design of immunogens targeting HIV V2-apex broadly neutralizing antibody precursors, *In press*.

STAR★METHODS

KEY RESOURCES TABLE

REAGENT or RESOURCE	SOURCE	IDENTIFIER
Antibodies		
Rat monoclonal anti-mouse-CD16/32 purified (clone 2.4G2)	BD Biosciences	CAT# 553142; RRID: AB_394657
Rat monoclonal anti-mouse CD4 APC-eF780 (clone: RM4-5)	Thermo Fisher Scientific	CAT# 47-0042-80; RRID: AB_1272219
Rat monoclonal anti-mouse CD8 APC-eF780 (clone: 53–6.7)	Thermo Fisher Scientific	CAT# 47-0081-80; RRID: AB_1272221
Rat monoclonal anti-mouse F4/80 APC-eF780 (clone: BM8)	Thermo Fisher Scientific	CAT# 47-4801-80; RRID: AB_2637188
Rat monoclonal anti-mouse Ly-6G APC-eF780 (clone: RB6-8C5)	Thermo Fisher Scientific	CAT# 47-5931-80; RRID: AB_1518805
Rat monoclonal anti-mouse B220 BV510 (clone: RA3-6B2)	BD Biosciences	CAT# 563103; RRID: AB_2738007
Hamster monoclonal anti-mouse CD95 PE-Cy7 (clone: Jo2)	BD Biosciences	CAT# 557653; RRID: AB_396768
Rat monoclonal anti-mouse CD38 A700 or A488 (clone: 90)	Thermo Fisher Scientific, Biolegend	CAT# 56-0381-82, 102714; RRID: AB_657740, AB_528796
Mouse monoclonal anti-mouse CD45.2 PE (clone: 104)	Biolegend	CAT# 109808; RRID: AB_313445
Mouse monoclonal anti-mouse CD45.1 PerCP Cy5.5 (clone: A20)	Biolegend	CAT# 110728; RRID: AB_893346
Rat monoclonal anti-mouse GL7 A647 (clone: GL7)	Biolegend	CAT# 144606; RRID: AB_2562185
Rat monoclonal anti-mouse IgM BUV395 or BV421 (clone: II/41)	BD Biosciences	CAT# 743329, 743323; RRID: AB_2741430, AB_2741424
Rat monoclonal anti-mouse IgD BV786 (clone: 11-26 c.2a)	BD Biosciences	CAT# 563618; RRID: AB_2738322
Rat monoclonal anti-mouse IgD PE-Cy7 (clone: 11-26 c.2a)	Biolegend	CAT# 405720; RRID: AB_2561876
Rat monoclonal anti-mouse IgG1 BV421 (clone: A85-1)	BD Biosciences	CAT# 562580; RRID: AB_2737664
Rat monoclonal anti-mouse Ig, κ light chain BUV395 (clone: 187.1)	BD Biosciences	CAT# 742839; RRID: AB_2741090
Goat Anti-Mouse IgG Fcγ ALP	Jackson ImmunoResearch	CAT# 115-055-071; RRID: AB_2338535
Chemicals, peptides, and recombinant proteins		
GT2 trimers	Produced in house	N/A
GT5 trimers	Produced in house	N/A
Biotinylated trimer probes	Produced in house	N/A
LIVE/DEAD™ Fixable Blue Dead Cell Stain Kit, for UV excitation	Thermo Fisher Scientific	CAT# L34962
Streptavidin-A488	Biolegend	CAT# 405235
Streptavidin-647	Biolegend	CAT# 405237
Streptavidin-PE	Biolegend	CAT# 405204
BD HORIZON BRILLIANT STAIN BUFFER	BD Biosciences	CAT# 566349
SIGMAFAST™ p-Nitrophenyl phosphate Tablets	Sigma	CAT# N2770-50SET
SuperScript™ III Reverse Transcriptase	Thermo Fisher Scientific	CAT# 18080085
RNasin® Ribonuclease Inhibitors	Promega	CAT# N2515
dNTP Mix (10 mM each)	Thermo Fisher Scientific	CAT# R0193
HotStarTaq DNA Polymerase	QIAGEN	CAT# 203209

(Continued on next page)

Continued

REAGENT or RESOURCE	SOURCE	IDENTIFIER
Critical commercial assays		
TaqMan	TransnetYX	N/A
CountBright™ Absolute Counting Beads, for flow cytometry	Thermo Fisher Scientific	CAT# C36950
UltraComp eBeads™ Compensation Beads	Thermo Fisher Scientific	CAT# 01-2222-42
Pan B Cell Isolation Kit II, mouse	Miltenyi Biotec	CAT# 130-104-443
BirA biotin-protein ligase bulk reaction kit	Avidity, Inc via Fisher Sci	CAT# NC9204985
Chromium Next GEM Single Cell 5′ Kit v2, 16 rxns	10x Genomics, Pleasanton, CA	CAT# 1000263
Chromium Next GEM Chip K Single Cell Kit, 48 rxns	10x Genomics, Pleasanton, CA	CAT# 1000286
Chromium Single Cell Mouse BCR Amplification Kit, 16 rxns	10x Genomics, Pleasanton, CA	CAT# 1000255
Library Construction Kit, 16 rxns	10x Genomics, Pleasanton, CA	CAT# 1000190
5′ Feature Barcode Kit, 16 rxns	10x Genomics, Pleasanton, CA	CAT# 1000256
Dual Index Kit TT Set A 96 rxns	10x Genomics, Pleasanton, CA	CAT# 1000215
Dual Index Kit TN Set A, 96 rxn	10x Genomics, Pleasanton, CA	CAT# 1000250
D5000 high sensitivity ScreenTape assay	Agilent, Santa Clara, CA	CAT# 5067-5588
Qubit™ dsDNA HS and BR Assay Kits	Invitrogen, Waltham, MA	CAT# Q32854
1x HBS-EP+ pH 7.4 running buffer	Teknova, Hollister, CA	CAT# H8022
Human Antibody Capture Kit	GE	CAT# BR-1008-39
Deposited data		
Cryo-EM map of GT2+GT2-d42.16	This paper; deposited to EMDB and PDB	EMDB: 25754<br>PDB: 7T9A
Cryo-EM map of GT5+GT5-d42.16	This paper	EMDB: 25755<br>PDB: 7T9B
BCR Sequences	This paper; deposited to NCBI GenBank	Heavy chains GenBank: OP287075–OP287644; Light chains GenBank: OP287645–OP287811
Experimental models: Organisms/strains		
Mouse: B6.SJL-Ptprcapepcb/BoyJ	The Jackson Laboratory	JAX: 002014
Mouse: C57BL/6J	The Jackson Laboratory.	JAX: 000664
Mouse: $PCT64^{LMCA}$	This paper	N/A
Mouse: $PCT64^{LMCA.JREV}$	This paper	N/A
Oligonucleotides		
Cocktails of IgG- and IgK-specific primers and thermocycling conditions described previously	von Boehmer et al. (2016)	N/A
sgRNA	Lin et al. (2018); Wang et al., (2021)	N/A
GT5-mRNA LNP	Moderna	N/A
Recombinant DNA		
Plasmids for PCT64 KI mouse lines	Produced in house	N/A
Software and algorithms		
FlowJo 10.7.1	Treestar	https://www.flowjo.com/
Prism 9.0.1	GraphPad	https://www.graphpad.com/
Microsoft Office	Microsoft	https://www.office.com/
IMGT/V-quest	Brochet et al. (2008); Giudicelli et al. (2011)	http://www.imgt.org/IMGTindex/V-QUEST.php
Geneious 2020.2	Biomatters	https://www.geneious.com
Cellranger	10x Genomics	https://support.10xgenomics.com/single-cell-gene-expression/software/pipelines/latest/what-is-cell-ranger

(*Continued on next page*)

Continued

REAGENT or RESOURCE	SOURCE	IDENTIFIER
FastTree	Price et al. (2010)	http://www.microbesonline.org/fasttree/
MUSCLE	Edgar (2004)	https://www.ebi.ac.uk/Tools/msa/muscle/
ProteOn Manager	Bio-Rad	CAT#: 1760200
Leginon	Suloway et al. (2005)	https://emg.nysbc.org/redmine/projects/legionon/wiki/Leginon_Homepage
MotionCor2	Zheng et al., (2017)	https://msg.ucsf.edu/software
Relion-3	Kimanius et al. (2016); Zivanov et al. (2018)	http://www2.mrc-lmb.cam.ac.uk/relion; RRID: SCR_016274
CryoSparc2	Punjani et al. (2017)	N/A
UCSF Chimera	Pettersen et al. (2004)	https://www.cgl.ucsf.edu/chimera/
COOT	Emsley and Crispin (2018)	N/A
Rosetta	Wang et al. (2016)	https://www.rosettacommons.org/
MolProbity	Chen et al. (2010)	https://github.com/rlabduke/MolProbity
EMRinger	Barad et al. (2015)	https://github.com/fraser-lab/EMRinger
Privateer	Agirre et al. (2015)	https://github.com/glycojones/privateer
UCSF ChimeraX	Pettersen et al. (2021)	https://www.cgl.ucsf.edu/chimerax/
Other		
Synergy Neo2 Plate Reader	BioTek	N/A
Armadillo PCR Plate, 96-well, clear, clear wells	Thermo Scientific	CAT# AB2396
Eppendorf twin.tec PCR Plate 96, semi-skirted, yellow	Genesee/Eppendorf	CAT# 951020320
Eppendorf twin.tec® PCR Plate 96, skirted, clear wells, blue	Genesee/Eppendorf	CAT# 951020460
Adhesive Sealing Sheets	Thermo Scientific	CAT# AB-0558
Microseal® ’F’ PCR Plate Seal, foil, pierceable #msf1001	Bio-Rad	CAT# MSF1001
E-Gel 96 2% with SYBR Safe	Thermo Fisher Scientific	CAT# G720802
E-Gel 96 Low Range Quantitative DNA Ladder	Thermo Fisher Scientific	CAT# 12373-031
BD FACSymphony™	BD Biosciences	N/A
BD FACS Aria II cell sorter	BD Biosciences	N/A
BD LSRFortessa	BD Biosciences	N/A
Nucleocounter	Chemometec	N/A
10x Genomics Chromium Controller	10x Genomics	N/A
MiSeq System	Illumina, San Diego, CA	N/A
ProteOn XPR36	Bio-Rad	Cat# 176-0100
GLC Sensor Chip	Bio-Rad	Cat# 76-5011
NanoDrop 2000 c Spectrophotometer	Thermo Fisher Scientific	N/A
Titan Krios	Thermo Fisher Scientific	N/A
Talos Arctica	Thermo Fisher Scientific	N/A
K2 Summit direct electron detector	Gatan	N/A
Superdex 200 10/300 GL	Cytiva/GE	Cat# 17517501
30KD Amicon Ultra	Millipore	Cat# Z717185
1.2/1.3 C-Flat holey carbon grids	Electron Microscopy Sciences	Cat# CDFT823-50
Vitrobot mark IV	Thermo Fisher Scientific	N/A

RESOURCE AVAILABILITY

Lead contact

Further information and requests for resources and reagents should be directed to and will be fulfilled by Facundo D. Batista (fbatista1@mgh.harvard.edu).

Data and code availability

- Cryo-EM maps and refined atomic models of GT2$^+$GT2-d42.16 and GT5$^+$GT5-d42.16 have been deposited in the EMDB and PDB under the accession IDs EMDB: 25754; PDB: 7T9A and EMDB: 25755; PDB: 7T9B, respectively. BCR sequence data have been deposited to GenBank and is available as of the date of publication (https://www.ncbi.nlm.nih.gov/genbank/). Accession numbers are listed in the key resources table.
- This paper does not report original code.
- Any additional information required to reanalyze the data reported in this paper is available from the lead contact upon request.

EXPERIMENTAL MODEL AND SUBJECT DETAILS

Mice

For experiments male B6.SJL-*Ptprc^{a}pepc^b*/BoyJ mice (CD45.1$^{+/+}$) between 7 and 12 weeks of age were purchased from The Jackson Laboratory (Bar Harbor, ME). F0-mice from the PCT64^{LMCA} KI mouse (CD45.2$^{+/+}$) colony were bred at the animal facility of the Gene Modification Facility (Harvard University) and breeding for colony expansion and experimental procedures was subsequently performed at the Ragon Institute of MGH, MIT and Harvard. Ear or tail snips from PCT64^{LMCA} KI mice were used for genotyping by TaqMan assay for a fee for service agreement (TransnetYX). TaqMan probes for the genotyping assay were developed by TransnetYX. CD45.2$^+$ B cells from PCT64^{LMCA} donor KI mice were enriched using the Pan B Cell Isolation Kit II (Miltenyi Biotec), counted, diluted to desired cell numbers in PBS and adoptively transferred into CD45.1$^+$ recipient mice as reported previously (Abbott et al., 2018).

All experiments were performed under the approval by the Institutional Animal Care and Use Committee (IACUC) of Harvard University and the Massachusetts General Hospital (MGH) and conducted in accordance with the regulations of the American Association for the Accreditation of Laboratory Animal Care (AAALAC). All animals were cared for in accordance with AAALAC standards in accredited facilities. All animal procedures were performed according to protocols approved by IACUC, specifically: Animal Study Protocols 2016N000022 and 2016N000286 (MGH).

Generation of PCT64^{LMCA} knock-in (KI) mice

PCT64^{LMCA} KI mice were generated following published protocols (Lin et al., 2018; Wang et al., 2021). In brief, the targeting vector 4E10 (Ota et al., 2013) was modified by the incorporation of human rearranged PCT64 LMCA VDJ (heavy chain construct) or VJ (light chain construct) sequences downstream of the promoter region and by elongation of the 5′ AND-3′ homology regions using the Gibson assembly method (NEB). The targeting vector DNA was confirmed by Sanger sequencing (Eton Bioscience Inc.).

Next, fertilized mouse oocytes were microinjected with a donor plasmid containing either the pre-rearranged PCT64 LMCA IGH with the mouse VHJ558 promoter, or the pre-rearranged PCT64^{LMCA} IGK with the mouse Vκ4-53 promoter (200 ng/μL); two pair of single-guided RNAs (sgRNAs, 25 ng/μL) targeting either the H or the κ locus; and AltR-Cas9 protein (50 ng/μL) and injection buffer (Wang et al., 2021). Following culture, resulting zygotes were implanted into the uteri of pseudopregnant surrogate C57BL/6J mothers.

METHOD DETAILS

Immunizations

Preparations of immunogens (GT2 and GT5 at 10 μg/mouse) were diluted in PBS at a volume of 100 μL/mouse for intraperitoneal (i.p.) injection or 50 μL/mouse for intramuscular (IM) injection and then mixed at a 1:1 ratio with Sigma adjuvant system (Sigma) for at least 25 min. The final formulation was injected i.p. (total volume of 200 μL/mouse) or IM in the thigh muscles of the hindlimb (total volume of 100 μL/mouse). For mRNA-LNP GT5 immunization (10 μg/mouse), preparation was defrosted and immediately diluted in PBS at a volume of 100 μL/mouse, and then injected IM in the thigh muscles of the hind limb.

Immunogen and FACS probe production

For flow cytometric probe binding ApexGT2 or -GT5 was biotinylated by BirA enzymatic reaction (Avidity, Inc) according to the manufacturer's protocol. Biotinylated GT2 or GT5 probes and respective -KOs were pre-reacted in independent tubes for at least 30 min in a 4:1 molar ratio with fluorescently labeled streptavidin (SA-A488 and/or SA-647). Reagents were then combined with fluorescently labeled antibodies for FACS-staining.

ELISA

Antigen specific antibody titers were detected by ELISA, using anti-His Ab (2 μg/ml) to capture GT2, GT5 or -KO antigen (2 μg/ml) on 96-well. Plates were washed 5 times with 0.05% Tween 20 in PBS, blocked with 100 μL of 3% BSA in PBS for 1 h at room temperature (RT), and washed again prior to incubation with 1:2 or 1:5 serially diluted mouse serum samples for 1 h at RT. Wells were washed and incubated with Alkaline Phosphatase AffiniPure Goat Anti-Mouse IgG (Jackson Immuno Research,#115-055-071) at 1:1,000 in PBS with 0.5% BSA for 1 h at RT. p-Nitrophenyl phosphate (Sigma, # N2770) dissolved in ddH_2O (50 μL/well, RT, 25 min) was used for detection. Absorbance at 405 nm was determined with a plate reader (Synergy Neo2, BioTek). ELISA curves were calculated and analyzed using GraphPad Prism 8.4.3 (GraphPad).

Flow cytometry

At selected time points following immunization, whole spleens were mechanically dissociated to generate single-cell suspensions. ACK lysis buffer was used to remove red blood cells and splenocytes were then resuspended in FACS buffer (2% FBS/PBS), Fc-blocked (clone 2.4G2, BD Biosciences) and stained for viability with Live/Dead Blue (Thermo Fisher Scientific) for 20 min at 4°C. For surface staining GT2 or GT5 probes (described above), as well as antibodies against CD4-APC-eF780, CD8-APC-eF780, Gr-1-APC-eF780, F4/80-APC-eF780, B220-B510, CD95-PE-Cy7, CD38-A700, CD45.1-PerCP-Cy5.5, CD45.2-PE, IgD-BV786, IgM-BUV395 and IgG1-BV421, were used. Cells were acquired by a BD LSRFortessa (BD Biosciences) for flow cytometric analysis and sorted using a BD FACS Aria II instrument (BD Biosciences). Data was analyzed using FlowJo software (Tree Star). B cells were single-cell dry-sorted into 96-well PCR plates, rapidly frozen on dry ice and stored at −80°C until processing.

BCR sequencing

Following single-cell sorting of antigen-specific B cells, the genes encoding the variable region of the heavy and light chains of IgG were amplified through RT-PCR. In brief, first strand cDNA synthesis was carried out using SuperScript III Reverse Transcriptase (Invitrogen) according to manufacturer's instructions. Nested PCR reactions consisting of PCR-1 and PCR-2 were performed as 25 μL reactions using HotStarTaq enzyme (QIAGEN), 10 mM dNTPS (Thermo Fisher Scientific) and cocktails of IgG- and IgK-specific primers and thermocycling conditions described previously (von Boehmer et al., 2016). PCR products were run on precast E-Gels 96 2% with SYBR Safe (Thermo Fisher Scientific) and wells with bands of the correct size were submitted to GENEWIZ company for Sanger sequencing. HC products were sequenced using the HC reverse primer from PCR-2 (5′-GCTCAGGGAARTAGCCCTT GAC-3′) and the LC was sequenced using the LC reverse primer (5′-TGGGAAGATGGATACAGTT-3′) from PCR-2.

Reads were quality-checked, trimmed, aligned, and analyzed using the Geneious software (Biomatters Ltd, New Zealand). IMGT/V-QUEST (http://www.imgt.org) (Brochet et al., 2008; Giudicelli et al., 2011) was used for mouse/Human Ig gene assignments. PCT64^{LMCA}-like mutation calculation were done as described previously (Briney et al., 2019; Soto et al., 2019).

10x Genomics B cell repertoire analysis

Naive splenic B cells were isolated from the PCT64H/L knock-in mouse line using the mouse, immunomagnetic negative selection, Pan-B cell Isolation Kit (Miltenyi Biotec, San Diego, CA). The B cell suspension was diluted 1:10 and cell density was manually determined using a hemocytometer. Cell viability was determined to be >90% by automated cell counter (Nucleocounter, Chemometec). Approximately 10,000 were loaded into the 10x Genomics Chromium Controller and encapsulated in gel beads in emulsion. Single-cell gene expression libraries were prepared using the Chromium Single-cell 5′ Library and Gel Bead Kit following the manufacturer's user guide (10x Genomics, Pleasanton, CA). The integrity of the library was determined using the D1000 high sensitivity ScreenTape assay (Agilent, Santa Clara, CA) and quantified using the Qubit fluorometry assay (AAT Bioquest, Sunnyvale, CA). BCR libraries were sequenced on the MiSeq System (Illumina, San Diego, CA) with 2 × 150 paired end reads (Genewiz, South Plainfield, NJ). Sequencing data produced from the Chromium Single Cell 5′ V(D)J library was analyzed using a customized the 10x Genomics Cell-ranger pipeline that includes the LMCA PCT64 heavy chain sequence (Genewiz, South Plainfield, NJ).

Phylogenetic analysis

Single paired amino acid sequences were joined and aligned using MUSCLE (Edgar, 2004). Clonal lineage trees were generated using FastTree (Price et al., 2010) and a Jones Taylor Thornton (Jones et al., 1992) model for AA evolution.

Surface plasmon resonance (SPR)

Kinetics and affinities of antibody-antigen interactions were measured on a ProteOn XPR36 (Bio-Rad) using GLC Sensor Chip (Bio-Rad) and 1x HBS-EP+ pH 7.4 running buffer (20x stock from Teknova, Cat. No H8022) supplemented with BSA at 1 mg/ml. Human Antibody Capture Kit was used according to manufacturer's instructions (Cat. No BR-1008-39 from GE) to immobilize about 6000 RUs of capture mAb onto each flow cell. In a typical experiment, approximately 300–400 RUs of mAbs were captured onto each flow cell and analytes were passed over the flow cell at 50 μL/min for 3 min followed by a 5 min dissociation time. Regeneration was accomplished using 3M Magnesium Chloride with 180 s contact time and injected four times per cycle. Raw sensograms were analyzed using ProteOn Manager software (Bio-Rad), including interspot and column double referencing, and either Equilibrium fits or Kinetic fits with Langmuir model, or both, were employed when applicable. Analyte concentrations were measured on a NanoDrop 2000 c Spectrophotometer using Absorption signal at 280 nm (Jardine et al., 2015).

Neutralization assay

Plasma and monoclonal antibodies neutralizing activity was assessed using single round of replication in TZM-bl target cells, as described previously (Landais et al., 2016) in presence of DEAE-dextran. Briefly, wild-type (WT) and mutant pseudoviruses were generated by co-transfection of 293 T cells with an Env-expressing plasmid and an Env-deficient genomic backbone plasmid (pSG3ΔEnv). Pseudoviruses were harvested 72 h post transfection for use in neutralization assays. Mutant pseudoviruses incorporating sets of amino acid mutations were generated by *de novo* gene synthesis and cloning (GeneScript).

Cryo-EM sample preparation

Purified ApexGT2 or ApexGT5 was incubated overnight at 4° with ~6 molar excess purified GT2-d42.16 or GT5-d42.16 Fab along with RM20A3 Fab then purified via size exclusion chromatography on a Superdex 200 Increase column followed by concentration of pooled fractions with a 30kD molecular weight cut-off Amicon Ultra centrifugal filter to a final concentration of ~3-7 mg/ml. Concentrated sample was mixed with 0.5μL of 0.04 mM lauryl maltose neopentyl glycol (LMNG; Anatrace) to a final concentration of 0.005 mM and 4 μL of this solution was applied to plasma cleaned 1.2/1.3 C-Flat holey carbon grids (Electron Microscopy Sciences) using a Vitrobot mark IV (Thermo Fisher Scientific) with a 7 s blot time, 0 blot force, and wait time of 0 s. Prepared grids were then stored in liquid nitrogen until they were transfer to a microscope for imaging.

Cryo-EM data collection

A table of detailed imaging conditions and data statistics for all the EM datasets is presented in Table S1. All datasets were collected with Leginon automated microscopy software (Suloway et al., 2005) on either an FEI Titan Krios operating at 300keV or an FEI Talos Arctica operating at 200keV (Thermo Fisher Scientific), both equipped with a K2 Summit direct electron detector (Gatan) operated in counting mode.

Cryo-EM data processing

All movie micrographs were aligned and dose-weighted using MotionCor2 (Zheng et al., 2017) and CTF parameters were estimated with GCTF (Zhang, 2016). Single-particle processing was carried out using a combination of Relion-3 (Kimanius et al., 2016; Zivanov et al., 2018) and CryoSparc2 (Punjani et al., 2017). The following general workflow was used for both datasets presented in this study. After frame alignment, dose-weighting, and CTF estimation, a subset of micrographs were selected based on CTF fit parameters and particle picking was performed, first using a gaussian blob, then templates from 2-D class averages. These particles were then subjected to one-two rounds of 2-D classification followed by subset selection, then one round of ab initio classification followed by subset. After subset selection, 3-Dautorefinement was performed with per-particle CTF correction followed by another round of 3-D classification using 3-D variability analysis. A soft spherical mask that surrounds the trimer apex and is large enough to accommodate the entire Fab was used to isolate variability in Fab occupancy followed by clustering into 3-6 classes. Clusters with clear density for Fab were then pooled and refined again together. 3-D variability was then employed again, this time to isolate variability in Fab binding angle followed by clustering and pooling of particles with similar angle of approach. Lastly, a final round of 3-D non-uniform refinement was performed to generate the final reconstruction.

Model building and figure preparation

For the GT2 + GT2-d42.16 Fab complex, the previously refined ApexGT2 (Willis et al., 2022) model was docked into the EM density map using UCSF Chimera (Pettersen et al., 2004) along with the previously refined atomic model of PCT64 LMCA and combined into a single PDB file. Mutations associated with GT5 relative to GT2 and d42.16 relative to the LMCA were then manually generated along with manual adjustment of glycans using COOT (Emsley and Crispin, 2018). This initial model was then relaxed into the EM density map using Rosetta (Wang et al., 2016) asking for ~300 models. All models were validated using MolProbity (Chen et al., 2010) and EMRinger (Barad et al., 2015) and the model with the best combined score was selected. All models were then checked and adjusted manually in COOT and re-refined with Rosetta, if necessary. Final models were then scored again with MolProbity and EMRinger, while glycan structures were further validated with Privateer (Agirre et al., 2015). Figures were prepared with either UCSF Chimera or ChimeraX (Pettersen et al., 2021). Hydrogen bonds were calculated and displayed with UCSF ChimeraX. Volume segmentation was performed with Segger (Pintilie et al., 2010) as implemented in UCSF ChimeraX. Figures were prepared in Adobe Illustrator (Adobe Inc.) and PowerPoint (Microsoft).

QUANTIFICATION AND STATISTICAL ANALYSIS

For immunization studies, statistical analysis was performed in Prism 9.01 (GraphPad) using two-tailed Mann-Whitney tests assuming non-normal distribution. p values less than 0.05 were considered significant (*p < 0.05; **p < 0.01; ***p < 0.001; ****p < 0.0001), as indicated in the figure legends.

For all mAb pseudovirus neutralization assays the IC50 (the concentration of mAb needed to obtain 50% neutralization against a given pseudovirus) was calculated from the non-linear regression of the neutralization curve. All neutralization assays were repeated at least twice, and data shown are from representative experiments.

Immunity, Volume *55*

Supplemental information

Membrane-bound mRNA immunogens lower the threshold to activate HIV Env V2 apex-directed broadly neutralizing B cell precursors in humanized mice

Eleonora Melzi, Jordan R. Willis, Krystal M. Ma, Ying-Cing Lin, Sven Kratochvil, Zachary T. Berndsen, Elise A. Landais, Oleksandr Kalyuzhniy, Usha Nair, John Warner, Jon M. Steichen, Anton Kalyuzhniy, Amber Le, Simone Pecetta, Manfredo Perez, Kathrin Kirsch, Stephanie R. Weldon, Samantha Falcone, Sunny Himansu, Andrea Carfi, Devin Sok, Andrew B. Ward, William R. Schief, and Facundo D. Batista

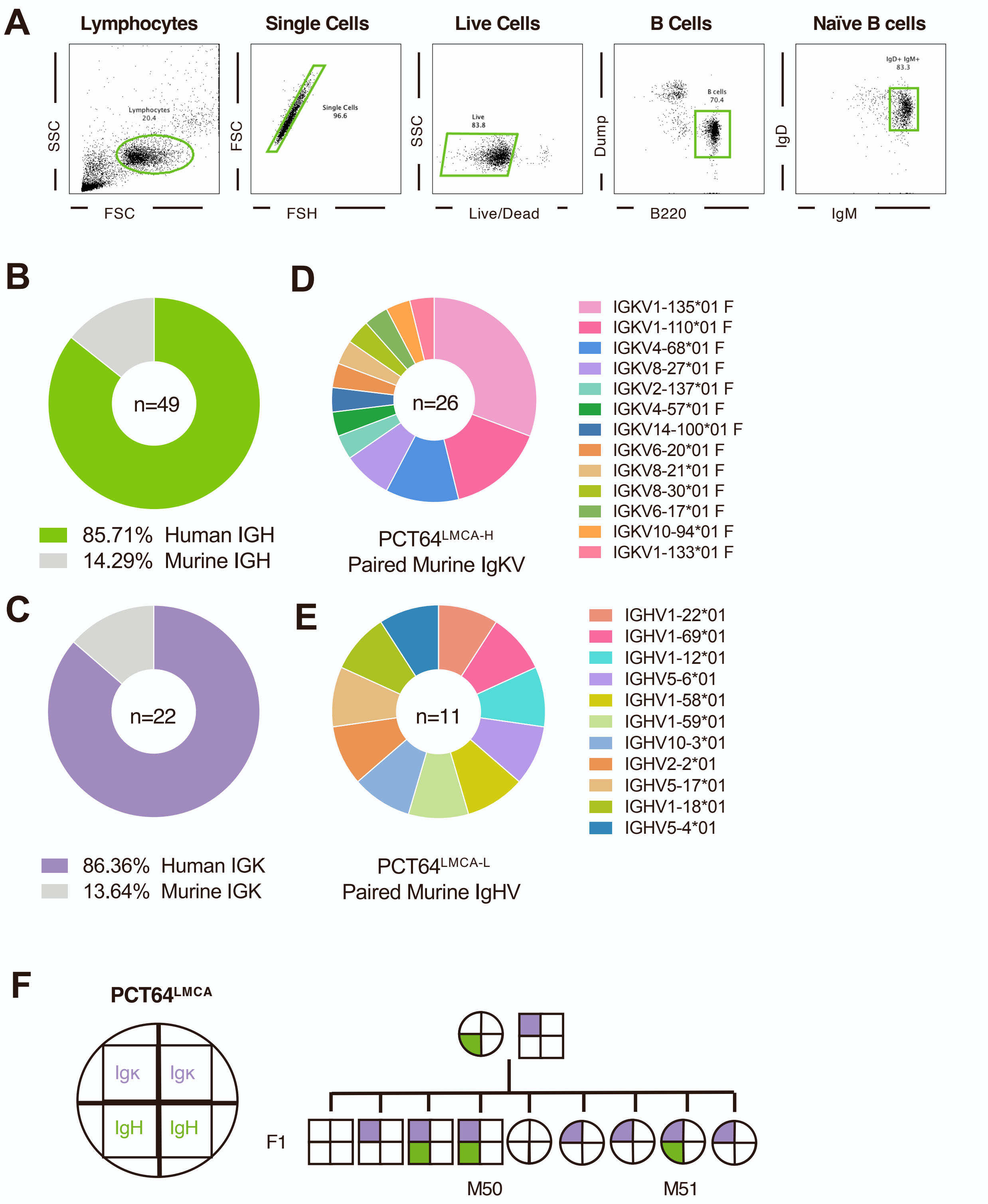

G

Line	# litters	# pups	# knock-in (KI) pups			KI pup frequency (%)		
			H	*κ*	*H, κ*	*H*	*κ*	*H, κ*
**LMCA**	6	49	22	22	11	44.8	44.8	22.4

Figure S1. Generation of a human Ig least mutated common ancestor (LMCA) PCT64 knock-in mouse, related to Figure 1.

A. Flow cytometry gating strategy for sorting and sequencing single naïve B cell from knock-in mice generated with CRISPR-Cas9.

B. Pie chart showing frequency of amplified human PCT64 LMCA heavy chain (green) and murine heavy chain (grey), from single cell BCR sequencing of naïve B cells in PCT64^{LMCA} IGH mice (PCT64$^{LMCA\text{-}H}$) (n=49).

C. Pie chart shows frequency of amplified human PCT64 LMCA light chain (purple) and murine heavy chain (grey), from single cell BCR sequencing of naïve B cells in PCT64 LMCA IGK mice (PCT64$^{LMCA\text{-}L}$) (n=22).

D. PCT64$^{LMCA\text{-}H}$ paired murine light chains V genes (n=26).

E. PCT64$^{LMCA\text{-}L}$ paired murine heavy chains V genes (n=11).

F. Breeding schematic for PCT64$^{LMCA\text{-}H}$ and PCT64$^{LMCA\text{-}L}$ mouse line. Squares represent males and circles represent females. Upper halves indicate IGK and lower halves IGH, as per key at left.

G. Transmission frequency of IGH (H) and IGK (K) to the progeny.

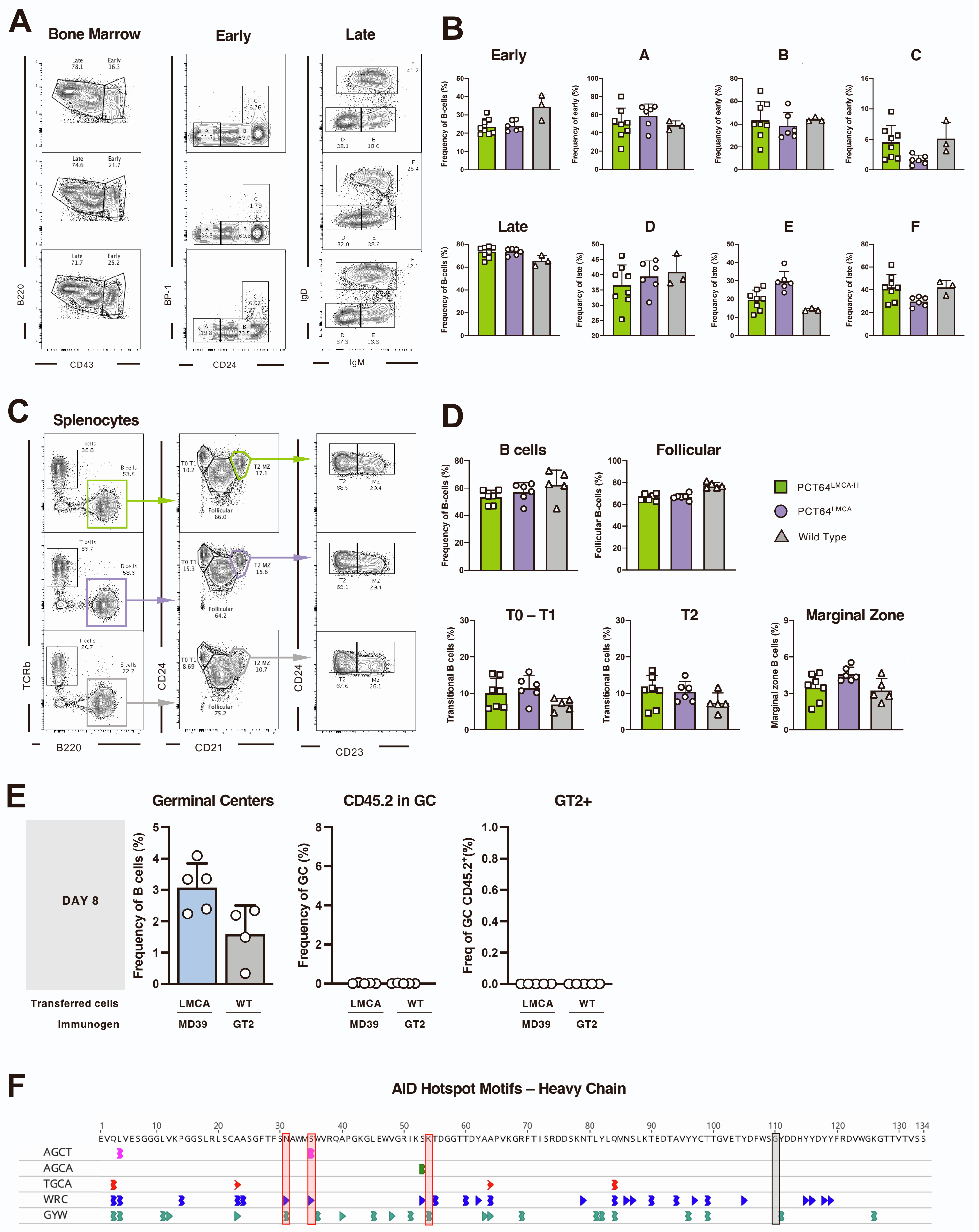
A
Bone Marrow
Early
Late
B220
CD43
BP-1
CD24
IgD
IgM
B
Early
A
B
C
Late
D
E
F
Frequency of B-cells (%)
Frequancy of early (%)
Frequancy of late (%)
C
Splenocytes
TCRb
B220
CD24
CD21
CD23
T cells
B cells
T0 T1
T2 MZ
Follicular
T2
MZ
D
B cells
Follicular
Frequency of B-cells (%)
Follicular B-cells (%)
PCT64LMCA-H
PCT64LMCA
Wild Type
T0 – T1
T2
Marginal Zone
Transitional B cells (%)
Marginal zone B cells (%)
E
DAY 8
Germinal Centers
CD45.2 in GC
GT2+
Frequency of B cells (%)
Frequency of GC (%)
Freq of GC CD45.2+(%)
Transferred cells
Immunogen
LMCA
WT
MD39
GT2
F
AID Hotspot Motifs – Heavy Chain
EVQLVESGGGLVKPGGSLRLSCAASGFTFSNAWMSWVRQAPGKGLEWVGRIKSKTDGGTTDYAAPVKGRFTISRDDSKNTLYLQMNSLKTEDTAVYYCTTGVETYDFWSGYDDHYYDYYFRDVWGKGTTVTVSS
AGCT
AGCA
TGCA
WRC
GYW

Figure S2. B lymphocyte development in PCT64^{LMCA-H} and PCT64^{LMCA} mice, related to Figures 1 and 2.

A. Representative FACS plots of bone marrow progenitor cells isolated from PCT64^{LMCA-H}, PCT64^{LMCA} and WT mice and gating strategy applied for the quantification of early (A, B and C) and late (D, E and F) subfraction of B cell developmental stages accordingly to (Hardy et al. 1991)

B. Quantification of early (A, B and C) and late (D, E and F) subfraction of B cell developmental stages in bone marrow from PCT64^{LMCA-H} (green), PCT64^{LMCA} (purple) and wild type (WT) (grey) mice (as in A). LMCA-H n=8, LMCA n=6, WT controls n=3. Data are presented as mean ± SD.

C. Representative FACS plots of splenocytes isolated from PCT64^{LMCA-H}, PCT64^{LMCA} and WT mice and gating strategy applied for the quantification of splenic B cells and their differentiation into Follicular, Transitional (T0, T1, T2) and Marginal Zone B cells.

D. Quantification of splenic B cells and their differentiation into Follicular, Transitional (T0, T1, T2) and Marginal Zone B cells in spleens from PCT64^{LMCA-H} (green), PCT64^{LMCA} (purple) and Wild Type (grey) mice (as in C). LMCA and LMCA-H n=6, WT controls n=5. Data are presented as mean ± SD.

E. Quantification of B cell responses in spleen at 8 days after immunization. CD45.1 WT mice received either 500,000 PCT64^{LMCA} B cells and were immunized with MD39-SOSIP trimers or received 500,000 wildtype CD45.2 B cells and were immunized with GT2 trimers. Experiments were performed in duplicate, one is shown (n=5). Data are presented as mean ± SD.

F. Analysis of the activation-induced deaminase (AID) enzyme binding sites in PCT64 LMCA heavy chain. AA position of enriched on-track mutations are highlighted (31, 35, 52b, 100d).

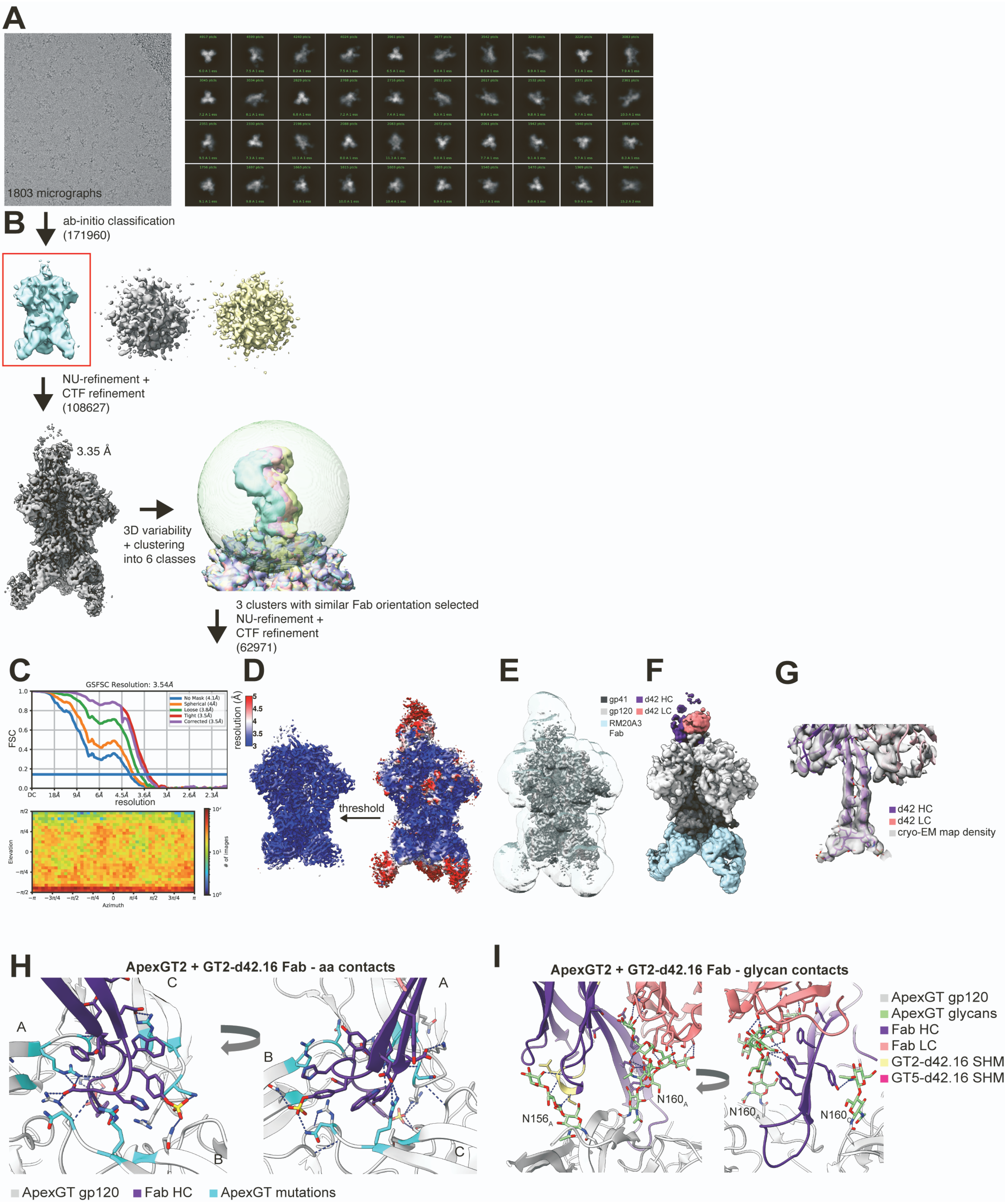
A
1803 micrographs
B
ab-initio classification (171960)
NU-refinement + CTF refinement (108627)
3.35 Å
3D variability + clustering into 6 classes
3 clusters with similar Fab orientation selected
NU-refinement + CTF refinement (62971)
C
GSFSC Resolution: 3.54Å
No Mask (4.1Å)
Spherical (4Å)
Loose (3.8Å)
Tight (3.5Å)
Corrected (3.5Å)
FSC
resolution
Elevation
Azimuth
# of images
D
resolution (Å)
threshold
E
gp41
gp120
RM20A3 Fab
F
d42 HC
d42 LC
G
d42 HC
d42 LC
cryo-EM map density
H
ApexGT2 + GT2-d42.16 Fab - aa contacts
ApexGT gp120
Fab HC
ApexGT mutations
I
ApexGT2 + GT2-d42.16 Fab - glycan contacts
N156A
N160A
ApexGT gp120
ApexGT glycans
Fab HC
Fab LC
GT2-d42.16 SHM
GT5-d42.16 SHM

Figure S3. Cryo-EM data processing workflow for ApexGT2 in complex with GT2-d42.16 Fab, related to Figure 2.

A. Raw EM micrograph and 2-D class averages of selected particles.

B. 3-D data processing workflow.

C. Fourier shell correlation and angular distribution plots.

D. Local resolution estimates.

E. Mask used for refinement and sharpening.

F. Map segmentation.

G. Isolated cryo-EM map density within a 3Å radius around Fab residues.

H. Close up of the epitope/paratope region of ApexGT2 + G2-d42.16 showing hydrogen-bonding interactions with gp120 amino acid residues.

I. Close up of the epitope/paratope region of ApexGT2 + GT2-d42.16 showing hydrogen-bonding interactions with ApexGT2 glycans.

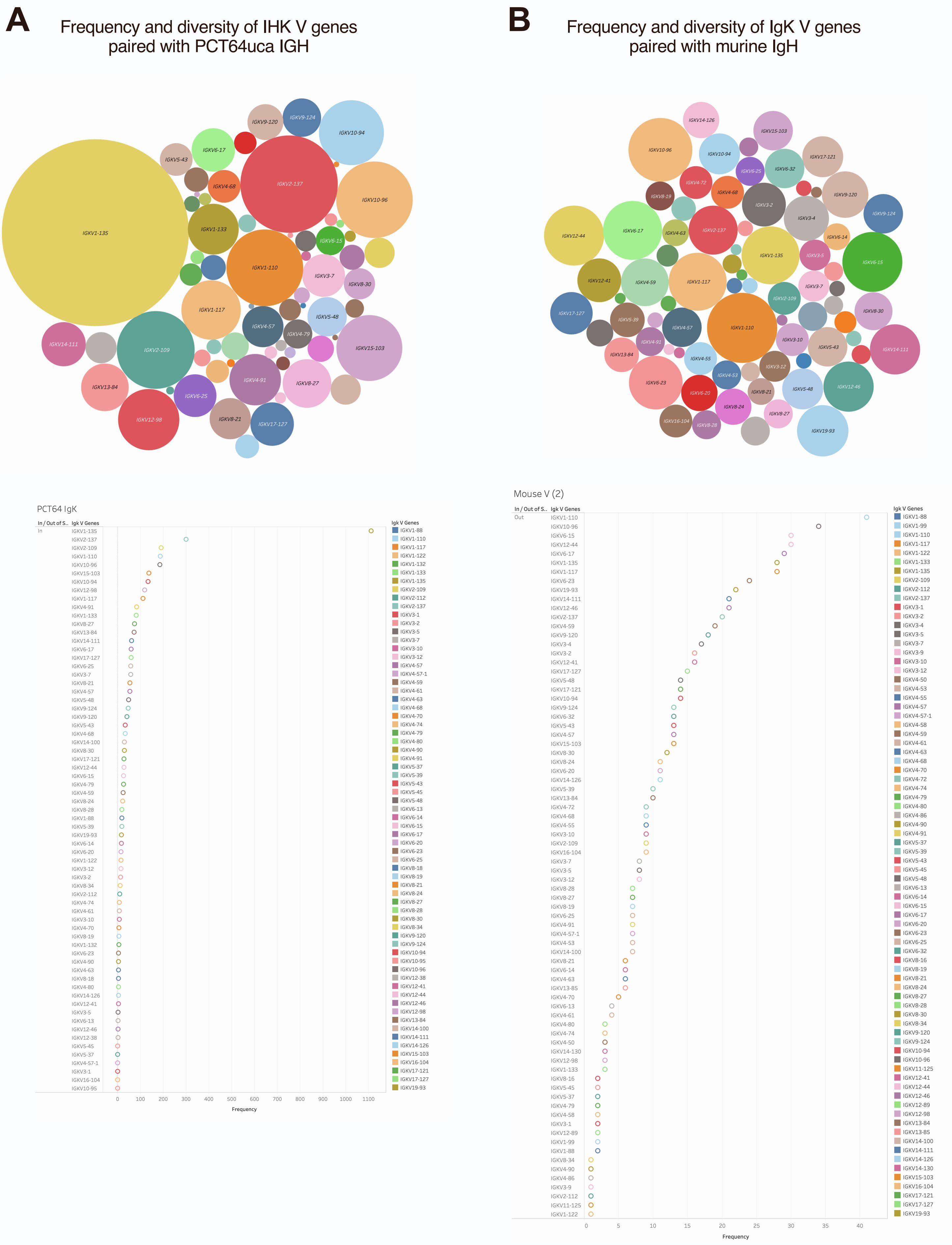
A
Frequency and diversity of IHK V genes paired with PCT64uca IGH
B
Frequency and diversity of IgK V genes paired with murine IgH
PCT64 IgK
In / Out of S.. Igk V Genes
Igk V Genes
0 100 200 300 400 500 600 700 800 900 1000 1100
Frequency
Mouse V (2)
In / Out of S.. Igk V Genes
Igk V Genes
0 5 10 15 20 25 30 35 40
Frequency

Figure S4. Light chain repertoire characterization in PCT64^{LMCA-H} mice, related to Figure 3.

A. Bubble graph representing frequency and diversity of murine IGK V genes paired with PCT64 IGH in a naïve PCT64^{LMCA-H} mouse (n=3888). Bubble size represent relative frequency, colors indicate different V gene. Below, detailed legend of all the isolated IGK V genes and relative frequency.

B. Bubble graph representing frequency and diversity of murine IGK V genes paired with murine IGH in a naïve PCT64^{LMCA-H} mouse (n=815). Bubble size represent relative frequency, colors indicate different V gene. Below, detailed legend of all the isolated IGK V genes and relative frequency.

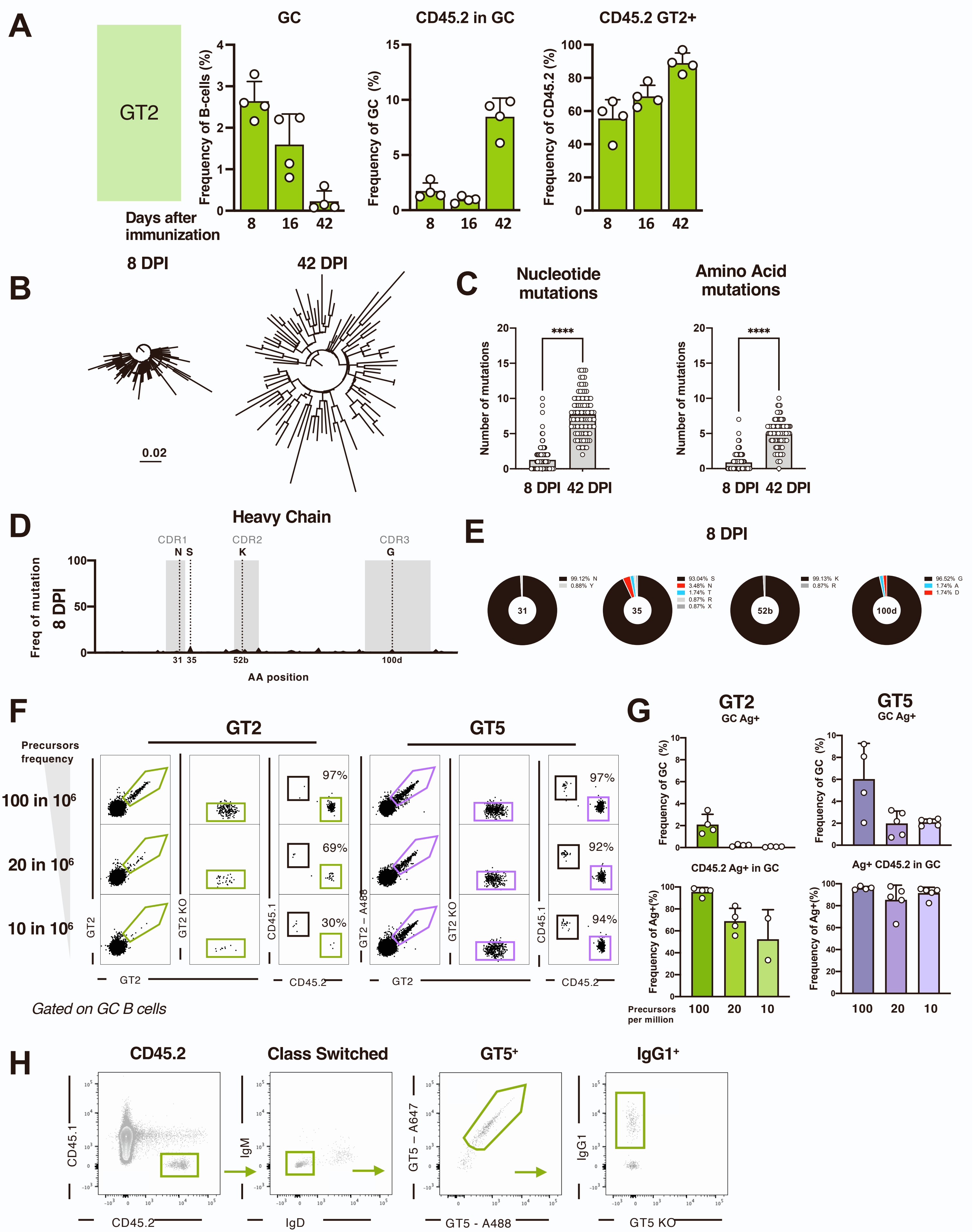
A
GT2
GC
CD45.2 in GC
CD45.2 GT2+
Frequency of B-cells (%)
Frequency of GC (%)
Frequency of CD45.2 (%)
Days after immunization
8
16
42
B
8 DPI
42 DPI
0.02
C
Nucleotide mutations
Amino Acid mutations
Number of mutations
****
8 DPI
42 DPI
D
Heavy Chain
CDR1
CDR2
CDR3
N S
K
G
Freq of mutation
8 DPI
31 35
52b
100d
AA position
E
8 DPI
31
35
52b
100d
F
GT2
GT5
Precursors frequency
100 in 10⁶
20 in 10⁶
10 in 10⁶
97%
69%
30%
97%
92%
94%
GT2
GT2 KO
CD45.1
CD45.2
GT2 – A488
Gated on GC B cells
G
GT2
GC Ag+
GT5
GC Ag+
Frequency of GC (%)
CD45.2 Ag+ in GC
Ag+ CD45.2 in GC
Frequency of Ag+(%)
Precursors per million
100
20
10
H
CD45.2
Class Switched
GT5⁺
IgG1⁺
CD45.1
IgM
GT5 – A647
IgG1
IgD
GT5 - A488
GT5 KO

Figure S5. GT2 immunization activates PCT64 heavy chain with different light chains and GC competition after GT2 and GT5 immunization, related to Figures 3, 4 and 5.

A. FACS quantification of germinal center (GC) responses, CD45.2 cells inside GC, and GT2 binding B cells at 8, 16 and 42 days after immunization with GT2. Recipient mice received 500,000 CD45.2^{+} PCT64$^{LMCA\text{-}H}$ B cells and responses were analyzed in the spleen. Experiments were performed in triplicate, one is shown (n=4/group). Data are presented as mean ± SD.

B. Phylogenetic clonal lineage trees showing diversification of the PCT64^{LMCA} IGH from day 8 to day 42 after immunization. Branch length is representative of sequence distance.

C. Number of total nucleotide and amino acid mutations acquired in PCT64$^{LMCA\text{-}H}$ heavy chain V genes (IGHV) at 8 (n=114) and 42 (n=106) DPI. P values calculated by Mann-Whitney test, ****P < 0.0001.

D. Frequencies of heavy chain AA mutations observed per residue at day 8 post-immunization in PCT64^{LMCA} heavy chain. HCDRs are highlighted in gray.

E. Distribution of selected PCT64 LMCA B cell heavy chain aa mutations in positions 31, 35, 52b and 100d at 8 dpi. Mutations present in mature PCT64 are in red, mutations present in PCT64-lineage early isolates are in blue, original LMCA amino acids are in black, other mutations are in grey.

F. Representative FACS plots of antigen-specific germinal center responses after immunization with GT2 (green) or GT5 (purple) (10 ug, adjuvanted with Sigma) in mice with 100, 20 and 10 per 10^6 PCT64^{LMCA} precursor B cells.

G. Quantification of responses in F. Experiments were performed in triplicate, one is shown (n=4, for GT5 10,20:10^6 n=5). Data are presented as mean ± SD.

H. FACS gating strategy for sorting of class switched antigen specific cells at 42 dpi.

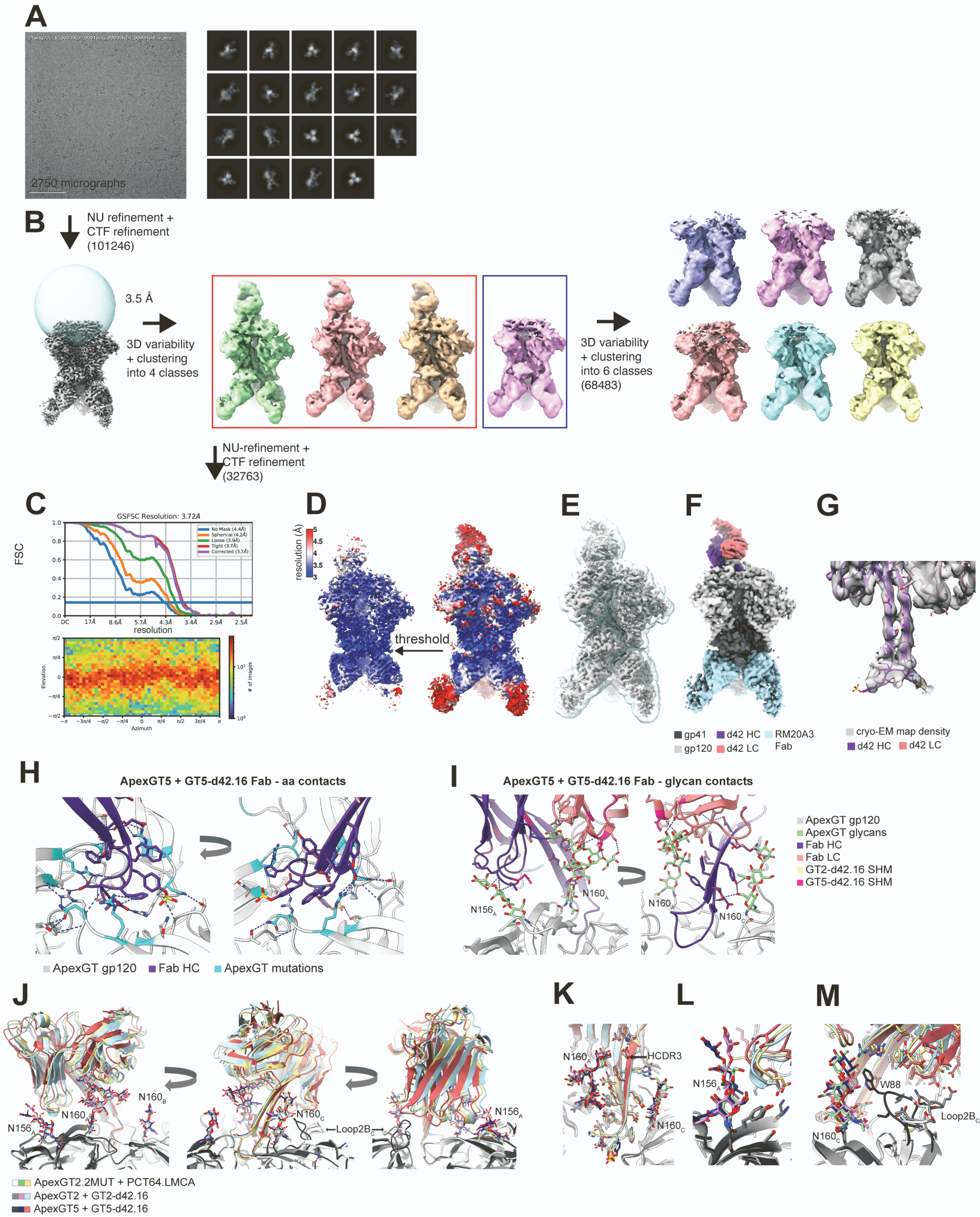

A
2750 micrographs
B
NU refinement + CTF refinement (101246)
3.5 Å
3D variability + clustering into 4 classes
3D variability + clustering into 6 classes (68483)
NU-refinement + CTF refinement (32763)
C
GSFSC Resolution: 3.72Å
FSC
No Mask (4.4Å)
Spherical (4.2Å)
Loose (3.9Å)
Tight (3.7Å)
Corrected (3.7Å)
resolution
Elevation
Azimuth
# of images
D
resolution (Å)
threshold
E
F
gp41
gp120
d42 HC
d42 LC
RM20A3 Fab
G
cryo-EM map density
d42 HC
d42 LC
H
ApexGT5 + GT5-d42.16 Fab - aa contacts
ApexGT gp120
Fab HC
ApexGT mutations
I
ApexGT5 + GT5-d42.16 Fab - glycan contacts
N156A
N160A
N160C
ApexGT gp120
ApexGT glycans
Fab HC
Fab LC
GT2-d42.16 SHM
GT5-d42.16 SHM
J
N156A
N160A
N160B
N160C
Loop2BC
ApexGT2.2MUT + PCT64.LMCA
ApexGT2 + GT2-d42.16
ApexGT5 + GT5-d42.16
K
N160A
HCDR3
N160C
L
N156A
M
W88
Loop2BC
N160C

Figure S6. Cryo-EM data processing workflow for ApexGT5 in complex with GT5-d42.16 Fab, related to Figure 5.

A. Raw EM micrograph and 2-D class averages of selected particles.

B. 3-D data processing workflow.

C. Global Fourier shell correlation and angular distribution plots.

D. Local resolution estimates.

E. Mask used for refinement and sharpening.

F. Map segmentation.

G. Isolated cryo-EM map density within a 3Å radius around Fab residues.

H. Close up of the epitope/paratope region of ApexGT5 + GT5-d42.16 showing hydrogen bonding interactions with gp120 amino acid residues.

I. Close up of the epitope/paratope region of ApexGT5 + GT5-d42.16 showing hydrogen bonding interactions with ApexGT2 glycans

J. Structures of ApexGT2.2MUT + PCT64.LMCA, ApexGT2 + GT2-d42.16, and ApexGT5 + GT5-d42.16 aligned and overlayed viewed from 3 different angles.

K. Close up of the HCDR3 domains and N160gp120A and B glycans,

L. N156gp120A glycan,

M. And loop2B of protomer C along with N160gp120A glycan.

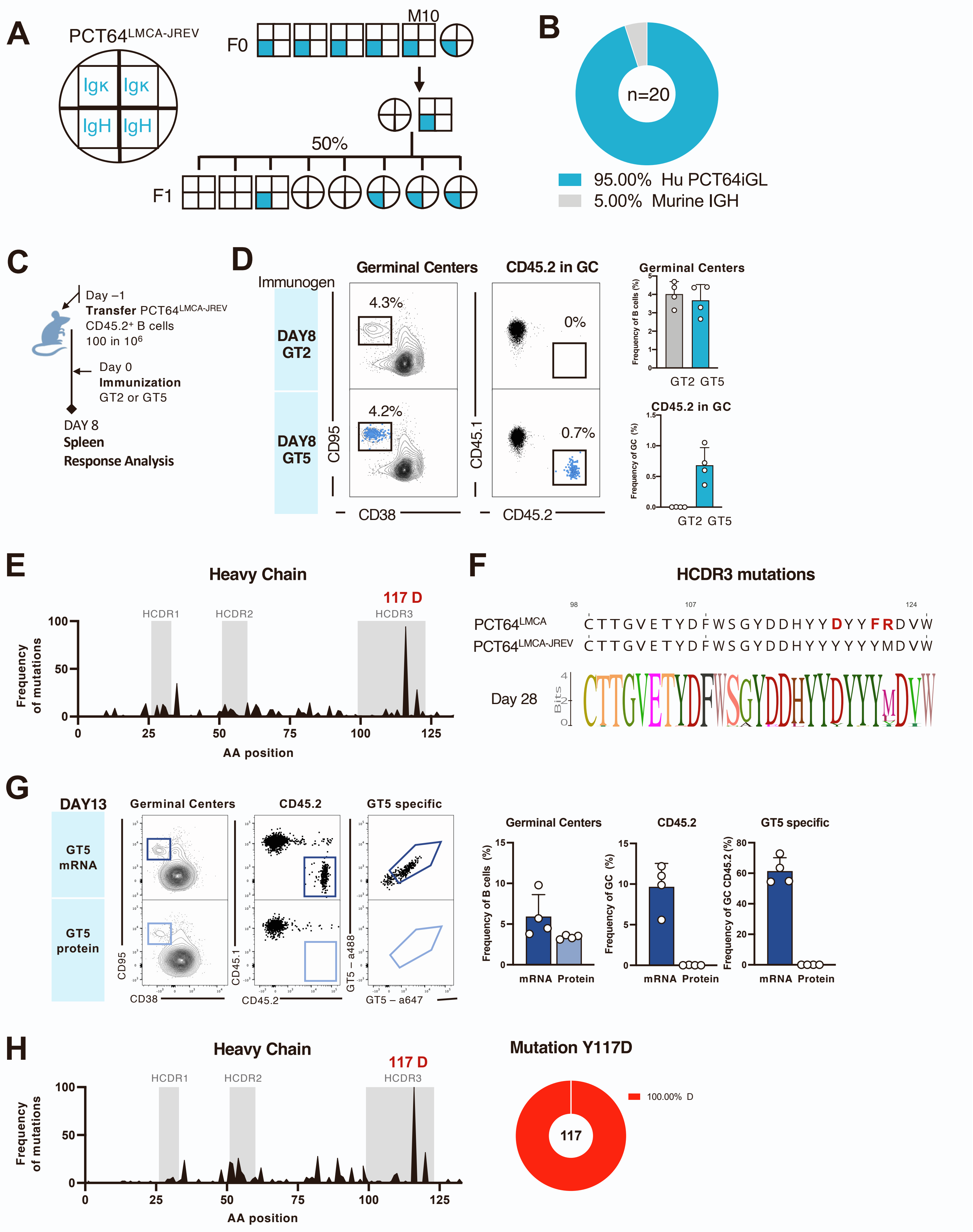

A
PCT64LMCA-JREV
Igκ
Igκ
IgH
IgH
F0
M10
50%
F1
B
n=20
95.00% Hu PCT64iGL
5.00% Murine IGH
C
Day –1
Transfer PCT64LMCA-JREV
CD45.2+ B cells
100 in 10⁶
Day 0
Immunization
GT2 or GT5
DAY 8
Spleen
Response Analysis
D
Immunogen
Germinal Centers
CD45.2 in GC
DAY8 GT2
DAY8 GT5
4.3%
4.2%
0%
0.7%
CD95
CD38
CD45.1
CD45.2
Germinal Centers
Frequency of B cells (%)
GT2 GT5
CD45.2 in GC
Frequency of GC (%)
GT2 GT5
E
Heavy Chain
117 D
HCDR1
HCDR2
HCDR3
Frequency of mutations
AA position
F
HCDR3 mutations
98
107
124
PCT64LMCA C T T G V E T Y D F W S G Y D D H Y Y D Y Y F R D V W
PCT64LMCA-JREV C T T G V E T Y D F W S G Y D D H Y Y Y Y Y M D V W
Day 28
Bits
G
DAY13
Germinal Centers
CD45.2
GT5 specific
GT5 mRNA
GT5 protein
CD95
CD38
CD45.1
CD45.2
GT5 – a488
GT5 – a647
Germinal Centers
Frequency of B cells (%)
mRNA Protein
CD45.2
Frequency of GC (%)
mRNA Protein
GT5 specific
Frequency of GC CD45.2 (%)
mRNA Protein
H
Heavy Chain
117 D
HCDR1
HCDR2
HCDR3
Frequency of mutations
AA position
Mutation Y117D
117
100.00% D

Figure S7. Generation of a PCT64 LMCA.JREV mouse and immunization strategies, related to Figure 7.

A. Breeding and transmission of the knock-in IGH PCT64$^{LMCA.JREV}$ to the progeny.

B. Pie chart showing frequency of amplified human PCT64$^{LMCA.JREV}$ heavy chain (teal) and murine heavy chain (grey), from single cell BCR sequencing of naïve B cells in a PCT64$^{LMCA.JREV}$ IGH (PCT64$^{LMCA.JREV\text{-}H}$) (n=20) mouse (M10).

C. Schematic of immunization study design. Recipient mice received 100 PCT64$^{LMCA.JREV}$ per10^6 B cells and were immunized IP with either GT2 or GT5 trimers, responses were analyzed 8 dpi.

D. FACS plots of germinal centers (GC) responses at 8 dpi after immunization with GT2 or GT5 (left). Quantification of CD45.2 responses in GC (right) (n=4). Experiments were performed in duplicate, one is shown. Data are presented as mean ± SD.

E. Localization of sites of mutation enrichment in the IGH of CD45.2 B cell isolated 42 days after GT5 protein immunization.

F. HCDR3 alignment of PCT64 LMCA, LMCA.JREV and consensus of mutations acquired 42 days after GT5 immunization.

G. FACS analysis of GC responses 13 days after IM immunization with either GT5 mRNA or GT5 soluble protein in PCT64$^{LMCA.JREV}$ recipient mice, quantification is on the right (n=4). Experiments were performed in triplicate, one is shown. Data are presented as mean ± SD.

H. Localization of sites of mutation enrichment in the IGH of CD45.2 B cell isolated 42 days after GT5 mRNA immunization (left). Pie chart with frequency of mutation Y117D (100% of 50 isolated IGH) (right).
